# Visible Photosensitizing Disinfectant Spray for Combating Multidrug‐Resistant *Candidozyma Auris* in Healthcare Settings

**DOI:** 10.1002/EXP.20250276

**Published:** 2026-03-10

**Authors:** Xiaoyu Xu, Ming‐Yu Wu, Baoping Li, Jie Li, Siyu Chen, Luojia Chen, Donghu Yu, Liupiaopiao Yang, Ziyu Hong, Huaqin Pan, Wei Xiang, Shun Feng, Jong Seung Kim, Lianrong Wang, Zhiqiang Li, Shi Chen, Meijia Gu

**Affiliations:** ^1^ Department of Neurosurgery, Zhongnan Hospital of Wuhan University Ministry of Education Key Laboratory of Combinatorial Biosynthesis and Drug Discovery School of Pharmaceutical Sciences Wuhan University Wuhan Hubei China; ^2^ College of Biomedical Engineering Sichuan University Chengdu China; ^3^ Sichuan Engineering Research Center for Biomimetic Synthesis of Natural Drugs School of Life Science and Engineering Southwest Jiaotong University Chengdu Sichuan China; ^4^ Department of Medical Intensive Care Unit Maternal and Child Health Hospital of Hubei Province Tongji Medical College Huazhong University of Science and Technology Wuhan Hubei China; ^5^ Hubei International Science and Technology Cooperation Base for Research and Clinical Techniques for Brain Glioma Diagnosis and Treatment Wuhan University Wuhan Hubei China; ^6^ Institute of Hepatobiliary Diseases of Wuhan University Transplant Center of Wuhan University Hubei Key Laboratory of Medical Technology on Transplantation Wuhan China; ^7^ Department of Critical Care Medicine Zhongnan Hospital of Wuhan University Clinical Research Center of Hubei Critical Care Medicine Wuhan China; ^8^ Department of Chemistry Korea University Seoul South Korea; ^9^ Institute of Pediatrics, Shenzhen Children’s Hospital Department of Respiratory Diseases Shenzhen China; ^10^ Department of Critical Care Medicine, Intensive Care Unit, Shenzhen Key Laboratory of Microbiology in Genomic Modification & Editing and Application, Shenzhen Institute of Translational Medicine, Shenzhen University Medical School, Shenzhen Second People’s Hospital The First Affiliated Hospital of Shenzhen University Shenzhen China

**Keywords:** *Candidozyma auris*, dual‐type AIE photosensitizer, hospital‐acquired infections, pH‐enhanced, polysaccharide

## Abstract

Developing simple and convenient strategies to eliminate drug‐resistant pathogen‐transmission routes efficiently is extremely urgent for hospital healthcare. *Candidozyma auris* (*C. auris*) is an emerging nosocomial pathogen with multidrug resistance that easily forms biofilms, aggravating the risk of spreading in public places. Herein, a novel visible light‐induced disinfectant spray with a boric‐acid‐functionalized lipophilic cation, TB, was developed. The boronic acid groups enable simultaneous targeting of polysaccharide‐rich cell walls and extracellular polymeric substances within biofilms. TB spray achieved a > 99.9% reduction in *C. auris* with light and reduced biofilm biomass by approximately 85.2%. TB's polysaccharide targeting, pH‐enhanced properties, positive charge, and particle size enable it to effectively bind to *C. auris* in acidic biofilm microenvironments, boosting photodynamic inactivation, collapsing biofilms, and preventing recurrence. It produces reactive oxygen species through Type I and II pathways, ensuring high efficacy even in hypoxic conditions, making it ideal for disinfecting high‐touch surfaces. In a rat model of ventilator‐associated pneumonia, TB markedly reduced pulmonary *C. auris* loads by over 100‐fold and significantly attenuated lung injury. Furthermore, the breakdown of polysaccharides enhanced the hydrophobicity of both abiotic and biological surfaces, as well as the increased contact angle, inhibiting biofilm adhesion. Multiomics analysis revealed that TB suppressed *C. auris* by disrupting genes associated with oxidative stress response, ergosterol biosynthesis, and biofilm maintenance. This visible light‐induced disinfectant spray holds great potential to combat outbreaks of high‐risk pathogens and could revolutionize disinfection practices in healthcare settings.

## Graphical abstract

We designed and synthesized a dual‐type AIE photosensitizer (named TB) spray, containing a cationic quaternary ammonium unit and a glycosyl‐targeted boronic acid moiety, characterized for the elimination of biofilm infections of *Candidozyma auris (C. auris)* on high‐touch surfaces. TB spray‐mediated photodynamic inactivation demonstrated an effective prevention effect in ventilator‐associated pneumonia in vivo, showing great promise in serving as the last line of defense against hospital‐acquired infections.

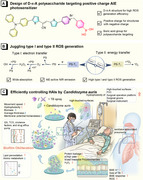

## 1 Introduction

Hospital‐acquired infections (HAIs) impose a substantial public health challenge, claiming over 140,000 lives globally each year [1, 2]. Intensive care units (ICUs) are considered high‐risk settings, with 30% of HAIs incidence [3, 4, 5]. The frequent occurrence of multidrug‐resistant (MDR) and even pan‐drug‐resistant pathogens in ICUs driven by immunocompromised patients and extensive antibiotic usage intensifies this challenge [6]. The World Health Organization declared antimicrobial drug resistance as one of the top 10 global public health threats facing humanity [7]. Biofilms are key contributors to the persistence and spread of HAIs [8, 9]. By forming protective matrices of extracellular polymeric substances (EPS), microbial communities adhere to high‐touch surfaces, such as medical devices, furniture, and implants, where they resist desiccation, disinfectants, and immune responses [10, 11]. The resilience of environmental contamination is amplified by biofilms, which confer resistance to dehydration and biocides through protective EPS [12, 13]. The IC50 for biofilms was 5–8 times higher than planktonic cells, with minimum inhibitory concentration thresholds surpassing 2000‐fold in biofilm‐associated infections [14]. Moreover, biofilm helps pathogens to overcome immune escape and even actively reprogram the host's innate immune system to favor recalcitrant infections and serious inflammation [15]. Ventilator‐associated pneumonia (VAP) is the most prevalent of ICU‐acquired infections in mechanically ventilated patients, as biofilms can easily transfer from contaminated high‐ touch surfaces to patients, which may occur in up to 50% of patients undergoing mechanical ventilation. Therefore, antifouling strategies on high‐touch surfaces have been designed as a significant alternative to combat microorganism adherence in ICUs.

Among all the pathogens causing HAIs, the fungal pathogen *Candidozyma auris* (*C. auris*, previously known as *Candida auris*), first described in 2009, undoubtedly stands out as a serious threat to global public health due to its outbreaks of ICU‐acquired infections across more than 50 countries [16]. *C. auris* fosters a cycle of acquisition, spreading, and infection, causing a staggering mortality rate of up to 60% [17]. The Centers for Disease Control and Prevention classified *C. auris* as an urgent antimicrobial‐resistant threat in March 2023, citing its escalating global health menace and treatment challenges [18, 19]. An article in the Lancet Microbe called for global high‐level attention to the super fungus *C. auris* to stress the importance of prevention [20]. Of note, the strong resistance of *C. auris* biofilms to environmental stress (including high temperature and salt concentration) allows its viability on the surface of the hospital environment for 2 weeks to 7 months [21]. As both fungi and mammalian cells are eukaryotic organisms, the similarity between them leads to a big challenge in developing new antifungal agents [22]. Known as “superbug”, *C. auris* shows high adaptability and strong resistance to most antifungal drugs and disinfectants, with up to 90% of strains resistant to fluconazole and almost all the strains showing resistance to echinocandins and polyenes [23]. This situation dramatically reduces the treatment efficacy [24]. In addition, poor immune function, recent surgery, recent use of antibiotics, and prolonged use of catheters all increase the risk of *C. auris* infection. The Joint Programming Initiative on Antimicrobial Resistance consortium has developed a comprehensive One Health framework that integrates six priority areas: environment, transmission, surveillance, diagnostics, therapeutics, and potential interventions to address antifungal resistance, which is in their “Strategic Research and Innovation Agenda on Antimicrobial Resistance” (Version 2021) [25]. Therefore, effective approaches to prevent the environmental transmission and enhance barrier precautions of *C. auris* are urgently needed, with greater clinical and practical significance than medical treatment.

Historically, the occurrence of one pandemic after another reminds us that human beings are far from well‐prepared in the face of infectious outbreaks [26, 27]. Thus, cutting off the pathogenic spreading route becomes an effective strategy for preventing HAIs [28]. Photodynamic inactivation (PDI) has emerged as a promising antimicrobial technology that utilizes light energy, non‐toxic photosensitizers (PS), and oxygen to generate reactive oxygen species (ROS) capable of eradicating microorganisms. It generates ROS via light activation, inducing oxidative damage to lipids, nucleic acids, and proteins, causing lethal cell death in pathogens, and represents a novel and effective non‐thermal inactivation approach [29, 30, 31]. Compared with conventional treatments, PDI offers distinct advantages such as non‐invasiveness, precise spatiotemporal control, negligible drug resistance, and minimized side effects [32]. PDI efficacy depends on PS type [33, 34]. Traditional aggregation‐caused quenching PSs suffer from aggregation‐induced self‐quenching, limiting imaging sensitivity and ROS generation [35]. Conversely, aggregation‐induced emission (AIE) PSs minimize nonradiative decay, narrow intersystem crossing energy gaps in aggregates, and enhance ROS output for improved antimicrobial efficacy [36, 37]. Accordingly, selectively targeting and microenvironmentally responsive PSs with abundant ROS generation show improved specificity by precision treatment and minimizing the damage to normal tissues, holding great potential for preventing and treating infections, thereby controlling pandemics [38, 39, 40, 41]. Despite great efforts devoted to developing PDI strategies for pathogenic infections, approaches for combating fungi are significantly understudied compared to bacteria [42, 43, 44, 45, 46, 47, 48]. This discrepancy may be attributed to the significant differences in composition, structure, function, and formation between fungi (eukaryotic) and bacteria (prokaryotic) [49, 50]. The cell wall containing polysaccharides ensures the morphological integrity and ecological adaptability of fungi through its multi‐faceted biochemical composition [51, 52], while the main component of the bacterial cell wall is a relatively simple structure composed of peptidoglycan [53]. What's more, the cell wall structural arrangement enhances fungal resistance to antifungals, complicating therapeutic management [54]. Besides, polysaccharides are indispensable components of biofilms, plasma membranes, and different intracellular membranes of fungal structures (such as mitochondria, Golgi bodies, ribosomes, and lysosomes), playing crucial roles in colonization, immune evasion, and signal transduction systems [55]. Based on the above, polysaccharides are ideal targets for the development of fungicidal therapeutics.

**SCHEME 1:**
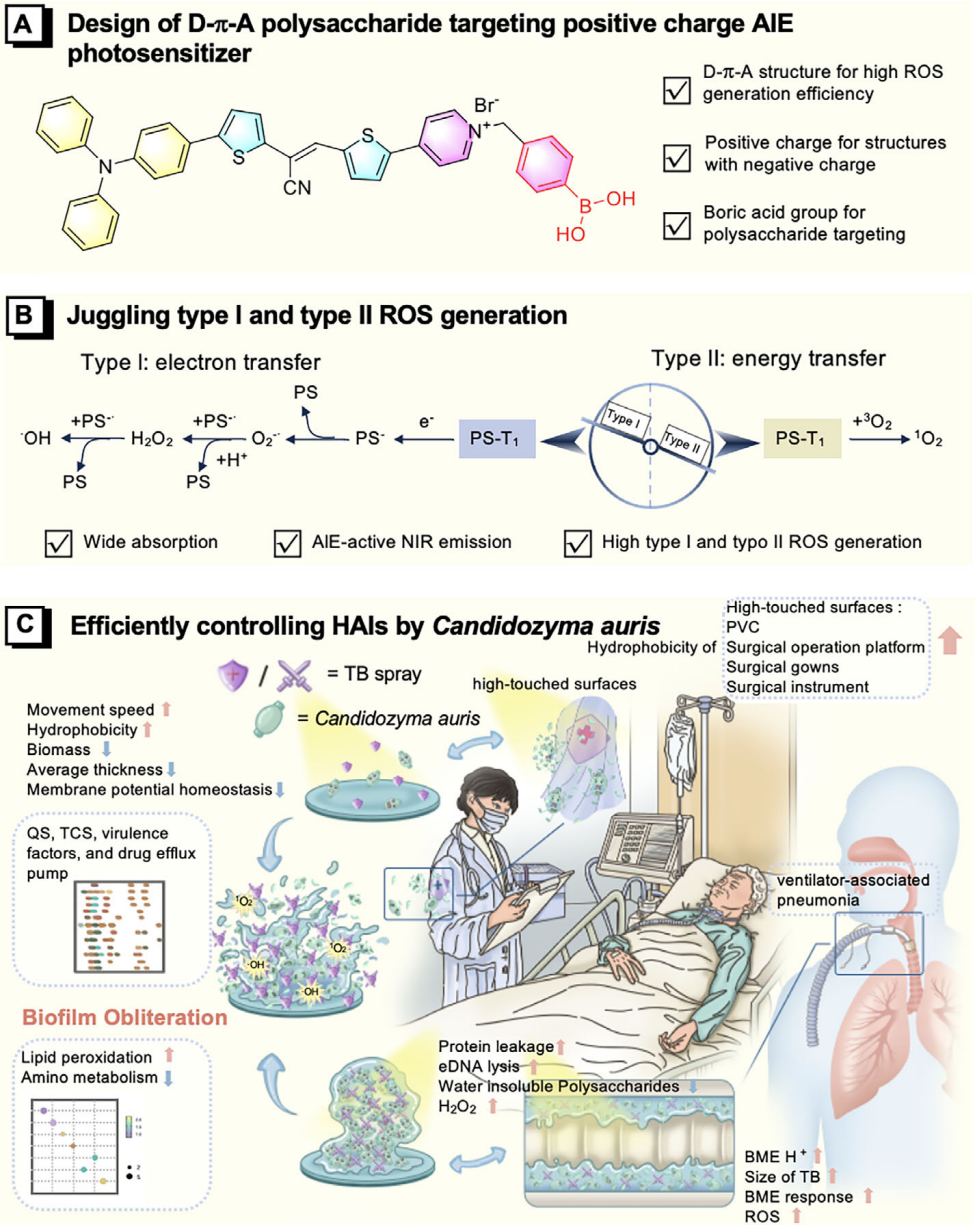
This schematic illustrates a dual‐functional AIEUnidentified photosensitizer spray designed for the precise elimination of *C*. *auris* biofilms and the prevention of ventilator‐associated pneumonia. (A) The molecular design integrates a cationic quaternary ammonium unit and a glycosyl‐targeting boronic acid moiety, enabling specific binding and penetration into fungal biofilms. (B) Upon irradiation, the agent simultaneously generates both Type‐I and Type‐II reactive oxygen species, ensuring potent antibiolfilm activity even under hypoxic conditions prevalent in biofilms. (C) Evaluated on high‐touch surfaces and in a rat ventilator‐associated pneumonia model, the spray‐mediated photodynamic inactivation effectively prevents biofilm formation and reduces pulmonary infection.

Herein, we report a novel visible light‐excited AIE PS spray (TB), exhibiting a high quantum yield and molar absorption coefficient, serving as a promising candidate for the last line of defense in the ICU. To increase the affinity of TB for obstinate negatively charged biofilms and cell walls of *C. auris*, we introduced a cationic quaternary ammonium unit and a polysaccharide‐targeting boric acid group. Additionally, under the acidic pH of the biofilm microenvironment (BME), the increase of positive charge of TB not only substantially boosts the binding affinity with negatively charged *C. auris* and biofilm but also enhances penetration and retention inside biofilms and disturbs membrane potential homeostasis, ultimately achieving destruction and extermination. TB achieves excellent highest occupied molecular orbital (HOMO) and lowest unoccupied molecular orbital (LUMO) isolation with a low energy gap (Δ*E*_S‐T_), promoting an efficient intersystem crossing (ISC) process. TB simultaneously generates strong type I and type II ROS, including hydroxyl radicals (•OH), which are highly lethal to living organisms even in the hypoxic environment, offering potential benefits in overcoming different BME challenges (Scheme 1) [56]. To explore the potential of TB spray in controlling HAIs, a series of TB pre‐coated high‐touch surfaces was conducted for the ICU simulation experiment, which demonstrated higher disinfection efficiency compared to conventional methods. In vivo experiments revealed that visible light‐mediated PDI effectively prevented VAP in a rat model. This treatment significantly protected the lungs and prevented the damage (pulmonary edema, inflammation, etc.) caused by *C. auris*, showing excellent infection‐preventing ability. Through multi‐omics analyses, we rigorously studied the molecular mechanisms of TB spray antifungal and antibiofilm properties. Transcriptomic analysis indicated that TB inhibits the quorum sensing (QS), two‐component system (TCS), biofilm virulence and adhesion, and efflux pumps, as well as adaptive multicellular morphology evolution, revealing the molecular mechanism underlying how TB spray conquers robust biofilm. Furthermore, metabolomics analysis demonstrated that TB‐mediated PDI strongly induced lipid peroxidation as well as synthesis and metabolism of essential amino acids in the mature biofilm, effectively inhibiting biofilm recurrence. What is more exciting is that, unlike ultraviolet (UV), TB spray‐mediated PDI does not cause denaturation of polyvinyl chloride (PVC), which is made into many medical devices and furniture. The high biosafety and biocompatibility of TB spray facilitate its availability in clinical practice. Since polysaccharides are ubiquitous in cell walls (both bacteria and fungi), TB spray has a promising broad‐spectrum inactivation effect on all pathogens. Meanwhile, TB spray with robust PDI can also be applied to fight against other biofilm infections among patients receiving mechanical ventilation [57]. This work not only provides an efficient strategy for clinical applications to prevent outbreaks of HAIs and serve as the last line of defense but also offers forward‐looking guidance for the rational design of next‐generation antimicrobial materials.

## 2 Results and Discussion

### 2.1 Molecular Design, Synthesis, and Photophysical Properties

**FIGURE 1:**
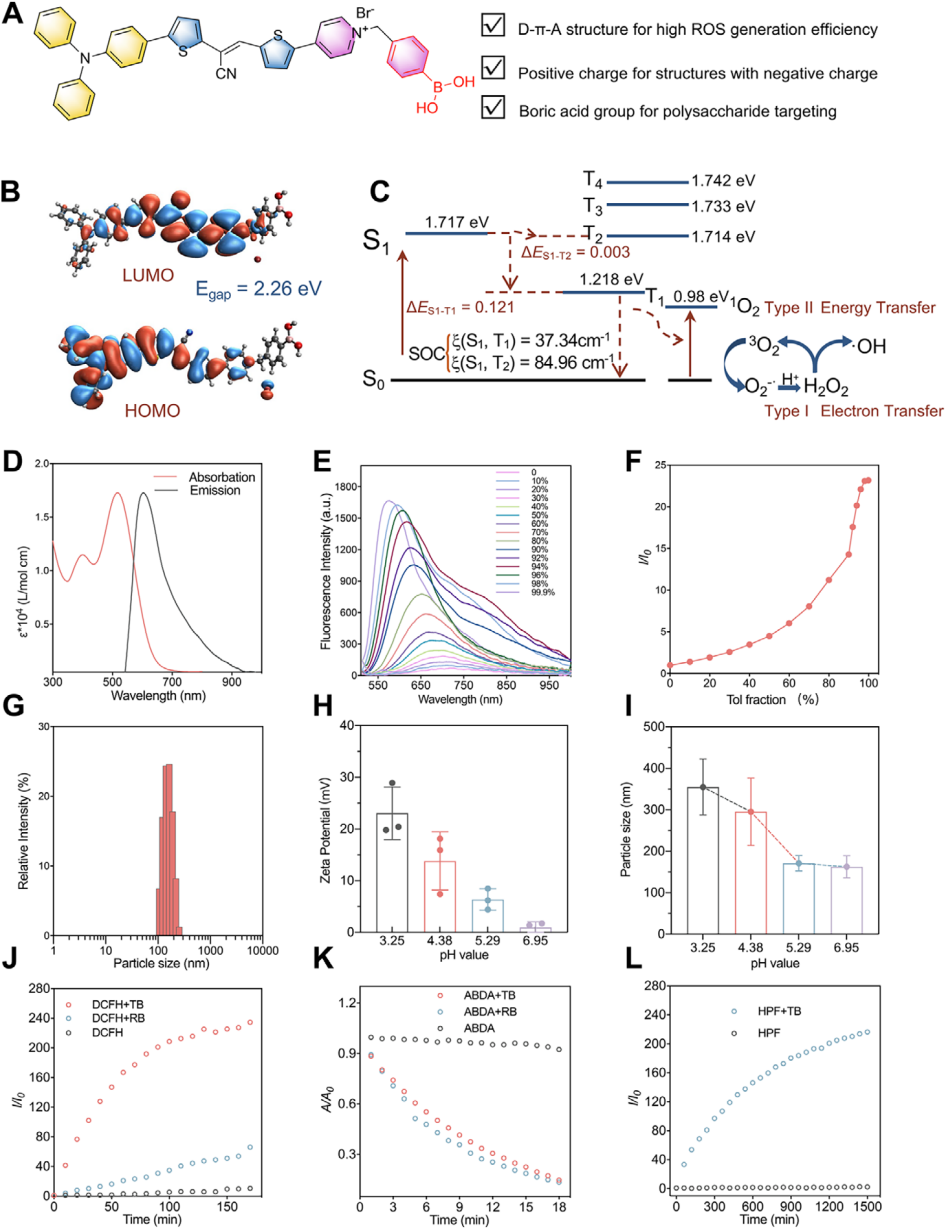
Molecular structure and photophysical properties of TB. (A) Schematic illustration of the structural characteristics of TB, (B) HOMO and LUMO distribution and Δ*E*_S‐T_ values of TB calculated by TD‐DFT at the basis set level of 6‐31G(d,p), (C) Computational result of energy levels and probable ISC pathways from S_1_ state to multiple low triplet states, (D) The molar absorption coefficient and photoluminescence spectra of TB (10 µM) in DMSO, (E) Photoluminescence spectra of TB (10 µM) in mixtures of DMSO and PhMe with different PhMe fractions, (F) Plot of the relative emission intensity of TB versus PhMe fraction. *I*_0_ and *I* are the peak photoluminescence intensity values of TB in DMSO and the DMSO/PhMe mixtures, respectively, (G) Size distribution of TB in water, (H) Zeta potential value of TB under different pH values (pH = 2.6, 4.3, 5.7, and 7.2) in H_2_O, (I) Hydrodynamic size distributions of TB under different pH values (pH = 2.6, 4.3, 5.7, and 7.2) in H_2_O, (J) Determination of ROS generation by TB upon white light illumination. Relative changes in fluorescence intensity (*I*/*I*_0_) of DCFH with or without TB in PBS at 534 nm under white light illumination at different time points, (K) Decomposition rates of ABDA in the presence or absence of TB or RB under light illumination (20 mW cm^−2^), where *A*_0_ and *A* are the initial and final absorbance of ABDA at 378 nm, respectively, (L) Determination of •OH generation by TB upon white light illumination. Relative changes in fluorescence intensity (*I*/*I*_0_) of HPF with or without TB in PBS at 515 nm under white light illumination at different time points.

To design optimal strategies to control HAIs caused by *C. auris*, we focused on the properties of BME: hypoxia, acidity, richness in polysaccharides, and negative charge [58]. In our previous work, we developed an AIE‐active plasma membrane‐targeted near‐infrared fluorescent probe, TBTCP [59, 60]. The dual‐functional amphiphilic structure's twisted D‐π‐A non‐planar geometry mimics membrane‐bound phospholipids, boosting electronic delocalization for higher ROS efficiency and targeted membrane interactions. The pre‐designed positive charge of TBTCP also shows a tendency to bind to negatively charged cell membranes and biofilms. As the markedly thickened mannan layer of *C. auris*, an additional boric acid group was added to the pyridinium group to further increase the binding ability of AIEgens to EPSs of *C. auris* biofilms, which is named TBTCP‐BOH. For convenience, we simply refer to TB as TBTCP‐BOH throughout the article (Figure 1A). It is well‐known that the boric acid group was highly efficient in binding to the cis‐diol groups present in the glycans abundant in cell walls, EPS, and glycoproteins in the plasma membrane. Synthetic procedures for TB (Scheme S1) and its characterization via ^1^H NMR, ^13^C NMR, and HRMS are provided in the Supporting Information (Supplementary Figures S1–S3).

As shown in Figure 1B and Supplementary Figure S4, the electronic distribution of the HOMOs for TB is predominantly located in triphenylamine and thiophene groups. The electronic distribution of the LUMOs for TB is mainly delocalized on pyridine moieties. The separated distribution of HOMO and LUMO proves the charge transfer characteristics of TB. Further, we calculated the singlet and triplet excited states of TB using the b3lyp/6‐31G (d, p) basis set (Figure 1C). Our calculations revealed a notably small energy gap between S_1_‐T_2_ Δ*E*_S‐T_ (0.003 eV) of TB, which facilitates the strong intersystem conversion over fluorescence emission. Besides, the energy level of the T_1_ state was calculated as 1.218 eV. According to the previously reported literature, the type II PDI required energy transfer to triplet oxygen, so the proximity between the T state of PSs and ^3^O_2_ is necessary [61]. According to our calculation, the T_1_ energy of TB was determined to be 1.218 eV (0.098 eV for ^3^O_2_), indicating a possible pathway of ^1^O_2_ generation. Therefore, TB is a promising dual‐type AIE photosensitizer. The photophysical properties of TB were characterized by ultraviolet‐visible spectrophotometry and photoluminescence spectrometry. In dimethyl sulfoxide (DMSO), TB showed a wide range of absorption from 300 nm to 800 nm, with a maximum value of about 516 nm, and emission ranging from 640 nm to more than 900 nm, with a peak value of about 603 nm, which can effectively reduce the interference of biological autofluorescence (Figure 1D).

The AIE behavior of TB was systematically investigated using DMSO/toluene (PhMe) solvent mixtures with varying PhMe fractions (fT). As shown in Figures 1E and F, pure DMSO exhibited negligible emission (λ_e__m_ = 721 nm, quantum yield (QY) = 0.13%). Upon increasing fT, dual AIE characteristics emerged: a blueshifted emission maximum, accompanied by a 23‐fold fluorescence enhancement (QY = 13.73%) in 99% toluene. This blueshift is attributed to suppressed twisted intramolecular charge transfer (TICT) quenching, while the enhancement results from aggregation‐triggered restriction of intramolecular motion (RIM). Dynamic light scattering (DLS) further confirmed TB's colloidal stability in aqueous media, displaying a 158 nm hydrodynamic diameter (Figure 1G). These data demonstrated the typical AIE properties of TB. To improve the antifungal effectiveness, the design of TB focuses on targeting the acidic microenvironment within the biofilm. The development of pH‐enhanced PSs can increase their accumulation in acidic biofilm. The zeta potential and particle size of TB were measured at different pH values. The zeta potential of TB aggregation gradually increased from 0.95 ± 1.08 mV at pH = 6.95 to 23.03 ± 5.09 mV at pH = 3.25 in Figure 1H, and the particle size increased from 162.63 ± 26.42 nm to 354.93 ± 67.38 nm in ddH_2_O in Figure 1I, and the trend of zeta potential and particle size of TB in PBS under different pH was similar to that in ddH_2_O (Supplementary Figure S5). The enhancement of zeta potential may be due to the aggregation of H^+^ around TB in an acidic solution, which increases the positive potential. This obvious increase in positive charge facilitates the binding affinity of TB to the negatively charged biofilms under acidic conditions, enhancing its penetration and retention inside biofilms. TB can aggregate into larger particles in more acidic environments, which may be triggered by hydrogen bond formation caused by acid‐induced protonation. Moreover, the enhanced particle size amplifies the AIE properties of TB, leading to stronger fluorescence emission and increased generation of ROS [62]. Therefore, the fungicidal effect of pH‐enhanced AIEgen TB may be further intensified by the acidic microenvironment of the biofilm, contributing to the eradication of mature biofilms.

### 2.2 ROS Generation of TB

The dual‐type AIE PS TB demonstrated exceptional ROS efficacy, outperforming conventional type II PSs. Figure 1J illustrates that TB induced a 235‐fold fluorescence enhancement of DCFH (525 nm) under light irradiation, contrasting with only 71‐fold for rose bengal (RB). Systematic evaluation of TB's ROS generation profile revealed dual type I/II activity. ABDA degradation kinetics (Figure 1K and Supplementary Figure S6) confirmed type II (^1^O_2_) production comparable to RB (The experiment with the probe SOSG also confirmed this result in Supplementary Figure S7) [63]. HPF oxidation (Figure 1L) demonstrated type I (•OH) generation, with a 216‐fold fluorescence increase attributed to TB's electron‐rich structure, enhancing electron transfer. Electron paramagnetic resonance spectroscopy is the gold standard technique for clearly identifying and distinguishing specific ROS. Using TEMP as a trap, the characteristic three‐line signal of TEMPO was detected, confirming the simultaneous production of ^1^O_2_. Furthermore, by using DMPO as a spin well [64], we observed a characteristic quartet signal characterizing the DMPO‐•OH adduct, which provides direct evidence for the generation of •OH (Supplementary Figure S8). This synergistic ROS generation capability enables TB to combat hypoxic biofilm pathogens through oxygen‐independent and oxygen‐dependent PDI pathways [65].

### 2.3 TB‐Mediated PDI to Inactivate Planktonic *C. auris*

**FIGURE 2:**
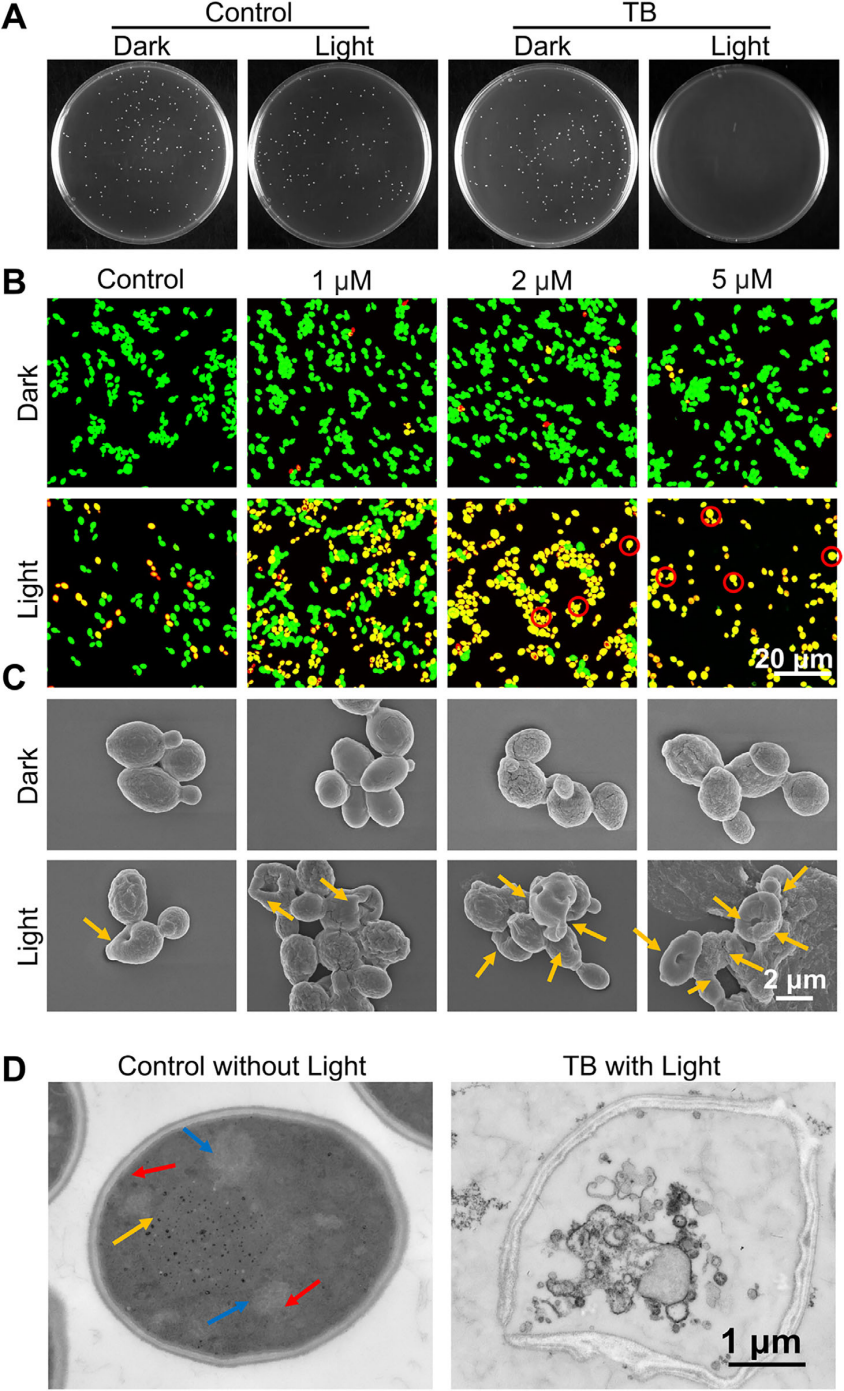
Photodynamic antifungal effect of TB. (A) Representative images of YPD agar plates employed to quantify *C. auris* viability. *C. auris was* treated with or without 5 µM TB, followed by storage in the dark or under white light irradiation (80 mW cm^−2^) for 20 min, (B) CLSM images of *C. auris* after applying a Live & Dead^TM^ Viability/Cytotoxicity assay kit and treatment with various concentrations of TB under white light irradiation (80 mW cm^−2^) or in the dark for 20 min. A 488 nm laser with a 515–550 nm emission filter (green channel) and a 561 nm laser with a 620–720 nm emission filter (red channel) were used for imaging. Scale bar: 20 µm. Red circles represent the multicellular aggregative phenotype of *C. auris*, (C) FESEM morphological images of *C. auris* incubated with different concentrations of TB with or without light irradiation for 20 min (80 mW cm^−2^). The yellow arrows indicate deformed or broken fungal structures. Scale bar: 2 µm, (D) TEM images of *C. auris* incubated with 5 µM TB under light irradiation for 20 min (80 mW cm^−2^). *C. auris* without treatment was the control. The yellow arrow indicates the cell nucleus, the red arrows show mitochondria, and the blue arrows show vesicles. Scale bar: 1 µm.

As one of the dominant pathogenic fungi responsible for HAIs, the “superbug” *C. auris* has recently emerged as a global public health threat [66]. Given that TB can generate significant amounts of ROS, we further investigate whether it could inactivate planktonic *C. auris* in vitro. The plate spot assay showed that after 20 min of exposure to white light (80 mW cm^−2^) in PBS, the viability of *C. auris* decreased to 91.67% (Figure 2A and Supplementary Figure S9), indicating a degree of sensitivity to white light alone. In the Live/Dead staining assay, all *C. auris* cells were stained with NucGreen (emitting green fluorescence), whereas dead cells were stained with PI (emitting red fluorescence) due to membrane and cell wall damage. Under white light exposure, enhanced red fluorescence was observed with increasing concentrations of TB, indicating dose‐dependent killing of planktonic *C. auris*. In addition, the representative multicellular aggregative phenotype of *C. auris* can be observed under TB and white light irradiation (red circle), suggesting attenuated virulence [67]. Both the plate spot and Live/Dead staining assays indicated that 5 µM TB achieved excellent PDI in vitro, resulting in the death of nearly all *C. auris* cells (Figure 2B and Supplementary Figure S10).

Destruction of the cell wall is a known effective strategy for killing fungi, as exemplified by echinocandins [68]. Polysaccharides, including glycoproteins, are ubiquitously distributed in microbial systems and play key roles in adhesion, immune evasion, and biofilm maturation. Beyond structural functions, these molecules regulate quorum sensing (QS) and two‐component systems (TCS) while enhancing multidrug efflux machinery [69]. Both TCS and QS are important signal transduction mechanisms that promote the production of virulence factors and biofilm formation [70]. Meanwhile, ζ‐potential analysis was used to investigate the binding ability of the TB to microbial surfaces. TB neutralized the ζ‐potential of *Staphylococcus aureus* (*S. aureus*, from −21.5 mV to −9.2 mV) and *C. auris* (from −4.20 to −0.74 mV) (Supplementary Figure S11). In contrast, no significant change was observed for *Pseudomonas aeruginosa* (*P. aeruginosa*). These results indicate that TB binds effectively to Gram‐positive bacteria and fungi with thick cell walls but less so to Gram‐negative bacteria with outer membranes, thereby disrupting membrane potential homeostasis.

Field emission scanning electron microscopy (FESEM) was used to examine the morphological changes on the surface of *C. auris* following TB treatment (Figure 2C). Untreated *C. auris* cells displayed round or oval shapes with continuous and intact cell walls and membranes. Cells treated with TB in the dark remained intact, whereas those exposed to TB and light showed obvious morphological distortions, collapse, and surface roughening, possibly due to targeted adhesion and damage by TB. Minor indentations were also observed under white light treatment alone, consistent with previous findings. At 5 µM TB under light, nearly all fungal cells exhibited pronounced indentations (yellow arrows) and substantial extracellular material release, indicating a severe disruptive effect (Figure 2C). Besides, transmission electron microscopy (TEM) was further employed to investigate the intracellular impact of TB‐mediated PDI. As shown in Figure 2D, in the control group (without any treatment), organelles such as the nucleus (yellow arrow), mitochondria (red arrows), and vesicles (blue arrows) were visible. After treatment with TB spray and white light (80 mW cm^−2^), the cell wall detached from the cell membrane, and the cytoplasm became heterogeneous, including a fragmented nucleus, dissolved organelles, and numerous vacuoles. It is hypothesized that the extracellular ROS disrupts the cell wall and cell membrane, leading to the efflux of intracellular contents and organelle destruction. These observations unambiguously revealed the potent destroying effect of ROS sensitized by TB. Owing to the polysaccharide targeting and high ROS sensitizing efficiency of cationic AIE PS TB, it demonstrated satisfying antifungal performance.

### 2.4 TB Spray Exhibits High Efficiency in Eradicating Biofilm

**FIGURE 3:**
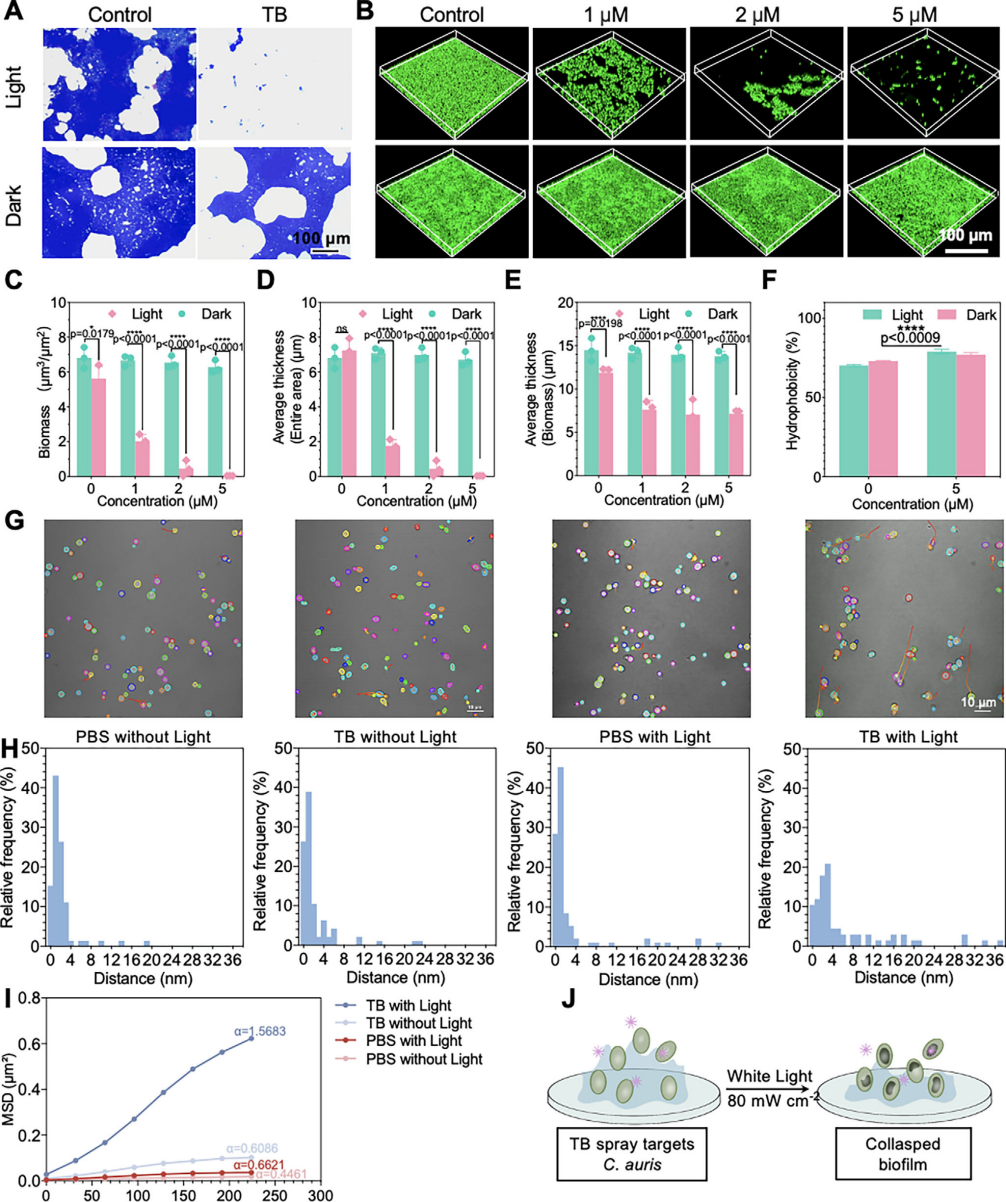
Photodynamic inhibition of *C. auris* biofilm formation with TB spray. (A–B) Representative crystal violet staining images (A) and (B) confocal images of *C. auris* biofilms after treatment with TB spray in the dark or under white light irradiation (80 mW cm^−2^) for 20 min (Scale bar: 100 µm), (C–E) The value of biomass (C), average thickness (entire area) (D) and average thickness (biomass) (E) of B), (F) The hydrophobic activity value of 0 and 5 µM TB spray under white light irradiation and in the dark, (G) *C. auris* movement visualization images with different treatments (Scale bar: 10 µm), (H) The trajectory distance frequency distribution histograms of *C. auris* with different treatments, (I) The MSD versus time interval of *C. auris* in the treatment groups, and (J) Schematic illustration of the inhibition of biofilm by TB spray. Data are presented as the mean ± SD of at least 3 replicates. Statistical significance between every two groups was calculated via one‐way ANOVA. ****p < 0.0001, ***p < 0.05, **p < 0.01, and *p < 0.001; **Abbreviation**: ns, not significant.

Microorganisms have inhabited everywhere in the biosphere and adapt to the environment with a variety of mechanisms, among which biofilm formation is one of the most important survival strategies [71]. Different from planktonic cells with efflux pumps or gene mutations as the mainstay of antifungal drugs, robust biofilm formation is the most important factor contributing to *C. auris* being an emerging fungal pathogen of increasing concern, with high drug resistance and mortality rates [72]. To evaluate the potential of TB spray to inhibit biofilm formation in *C. auris*, we employed a crystal violet staining assay and Confocal Laser Scanning Microscopy (CLSM) for biofilm quantification and 3D structural reconstruction. Crystal violet staining targets all biofilm substances, including fungi, extracellular polysaccharides, and eDNA, appearing in a blue color. As shown in Figure 3A and Supplementary Figure S12, there was no obvious change in biofilms after treatment with different concentrations of TB in the dark. However, once exposed to light, biofilm became loosely packed even at low TB spray concentrations (1 µM). Increasing TB levels led to dose‐dependent fragmentation of the biofilm. At a concentration of 2 µM, the biofilm appeared discontinuous with sporadic fungal clusters, and at 5 µM TB, biofilms were completely eradicated with no residual staining. Similar findings were obtained in the reconstruction of 3D biofilm using CLSM (Figure 3B and Supplementary Figure S13). Quantitative analysis of 3D biofilm architecture was performed using COMSTAT software (version 2.0), including measurements of average total thickness, average biofilm thickness, and biomass. As illustrated in Figure 3C, biofilms treated with light alone exhibited a multilayered structure, indicating active fungal proliferation and substantial biofilm formation. In contrast, pretreatment with TB spray under white light irradiation reduced the biofilm to a monolayer structure. Concentration of TB above 1 µM significantly altered the average thickness of the biofilm region, suggesting that ≤ 1 µM TB is sufficient to prevent vertical growth of the *C. auris* biofilm, restricting it to a monolayer. The average thickness and biomass of the entire observed region decreased from 7.25 µm and 5.62 µm^3^ µm^−2^ to 0.04 µm and 0.04 µm^3^ µm^−2^ with increasing TB concentration from 0 to 5 µM under white light irradiation (Figures 3D, E). Different from those in the *Candida* species, *C. auris* can form a multicellular aggregating phenotype, with increased adhesion and biofilm‐forming capacity [67]. As shown in Supplementary Figure S14, TB spray‐mediated therapy significantly increased *C. auris* autoaggregation, which might have contributed to the limitation of biofilm formation by TB spray.

Biofilm formation begins with the adhesion of microbial cells to the surface, which is a complex physicochemical process. In the initial stage of biofilm formation, microorganisms adhere to a surface, migrate across it, and become anchored. Then they release adhesins and EPS to reinforce attachment. The process is controlled by many factors, including microbial species, phenotypes, environment and surface properties (including roughness, surface charge, surface wettability, etc.) [73, 74]. Microbial cell surface hydrophobicity is a key determinant of the ability to adhere to inert surfaces [75]. Preventing pathogen adhesion on antifouling surfaces is often achieved by superhydrophobic effects [76]. Given that *C. auris* is sticky and can form biofilms on both living and abiotic surfaces, we next carried out the effect of TB spray on the surface hydrophobicity of *C. auris* using the microbial adhesion to hydrocarbons (MATH) method [77]. As shown in Figure 3F and Supplementary Videos S1 to S4, although the hydrophobicity of *C. auris* was slightly decreased by 20 min of 80 mW cm^−2^ light irradiation, the hydrophobicity was significantly enhanced by the synergistic treatment of TB spray and light irradiation, which implies that TB spray‐mediated PDI can also inhibit biofilm formation by reducing *C. auris* adhesion rate through the enhancement of hydrophobicity. Motility is crucial for many biological functions of *C. auris*, including biofilm formation and virulence [78]. The effect of TB spray‐mediated PDI on the motility of *C. auris* and the abiotic surface was also analyzed by tracking the movement path. *C. auris* in the logarithmic growth phase was treated with TB spray, and the control group was given PBS. The different groups of *C. auris* were placed in the light or dark for 20 min. The treated fungi were added to a confocal dish, and time‐lapse films of fungal motions were taken under a confocal microscope for 5 min. Videos were analyzed using the NIS‐Elements software's tracking plug‐in, which tracks individual fungi's movement and calculates the associated movement parameters to determine the adhesion of the fungi to the surface. The movement tracks of *C. auris* were converted into images. As shown in Figure 3G, slight swinging of a few *C. auris* was seen in the PBS‐only (no light) group (Supplementary Video S1), with similar behavior observed in the TB spray without light (Supplementary Video S2) and PBS with light group (Supplementary Video S3). In contrast, *C. auris* in the TB spray with the light group showed stronger swinging and longer movement distances. Analysis of movement distance distribution revealed that TB spray with light treatment resulted in longer displacements and a higher percentage of cells with large movement ranges, indicating that this treatment promotes fungal motility and inhibits surface adhesion (Supplementary Video S4). The movement trajectory of *C. auris* with different treatments is shown in Figure 3H. The relationship mean‐square displacement (MSD) = 4D(Δt)^α^ was used to calculate the α‐value (anomalous diffusion exponent) of the fungus under each treatment, where D denotes the distance that the fungi moved, and Δt denotes the time interval of each frame in the video [79]. The MSD curves versus time intervals are shown in Figure 3I. Among them, the movement of *C. auris* with TB spray under white light was super‐diffusion (α > 1, 1.5683), while the group treated with PBS without light was sub‐diffusion (α < 1, 0.4461) [80]. In summary, TB spray‐mediated photodynamic disinfection significantly inhibited biofilm formation integrity and thickness, enhanced hydrophobicity, and further suppressed the adhesion of *C. auris* (Figure 3J).

**FIGURE 4:**
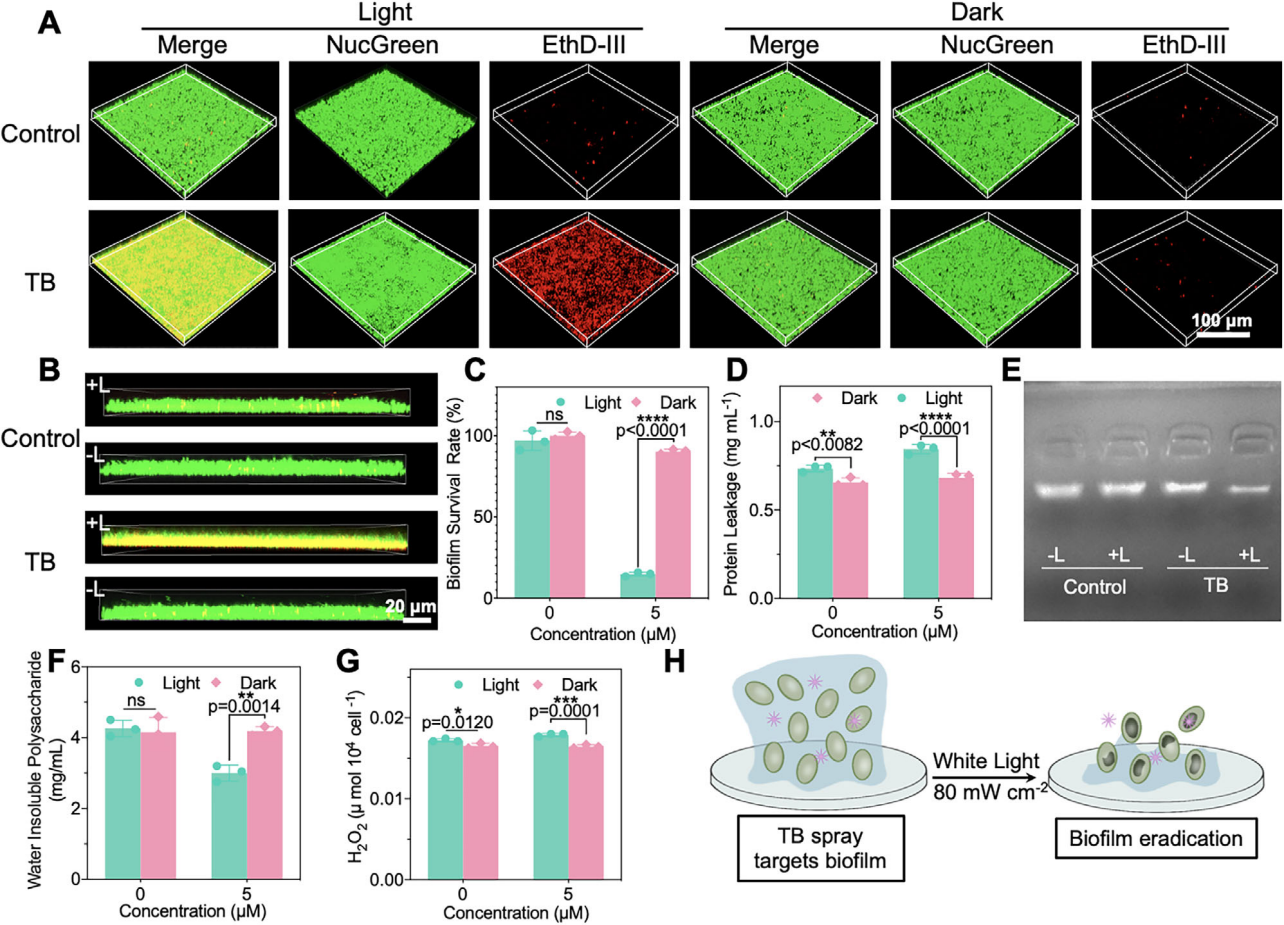
Photodynamic eradication of *C. auris* biofilms with TB spray. (A–B) Representative 3D images (A) and corresponding z‐stack images (B) of *C. auris* biofilms. Twenty‐four‐hour‐old mature biofilms were cocultured with 5 µM TB spray in the dark or irradiated with white light for 20 min (80 mW cm^−2^), followed by staining with a Live & Dead^TM^ Viability/Cytotoxicity assay kit. A 488 nm laser and a 515–550 nm emission filter were employed for the green channel, and a 561 nm laser and a 570–620 nm emission filter were used for the red channel (Scale bar: 100 µm and 20 µm), (C) Biofilm survival rate of A), (D) Protein leakage of biofilm after different treatments, (E) Agarose gel electrophoresis result of *C. auris* gDNA after different treatments, (F) The concentration of water‐insoluble polysaccharides in biofilm after different treatments, (G) The concentration of H_2_O_2_ in biofilm after different treatments, and (H) Schematic illustration of the eradication of biofilm by TB spray. Data are presented as the mean ± SD of at least 3 replicates. Statistical significance between every two groups was calculated via one‐way ANOVA. ****p < 0.0001, ***p < 0.05, **p < 0.01, and *p < 0.001; **Abbreviation**: ns, not significant.

Different from planktonic fungi, mature biofilms provide much stronger protection for embedded microorganisms through the 3D shield. Therefore, we next investigated the capability of TB spray to eliminate the mature biofilm. To evaluate the binding ability of TB spray and biofilm, we co‐incubated the biofilm with 5 µM of TB spray for 20 min. As shown in Supplementary Figure S15 and Supplementary Video S5, the red fluorescence of TB was uniformly distributed throughout the biofilm without a notable binding preference towards the fungi. Since *C. auris* biofilms contain abundant extracellular insoluble polysaccharides, we speculate that TB accumulates primarily on these polysaccharide matrices, resulting in widespread fluorescence. Additionally, we examined the intracellular and extracellular production of ROS in the biofilm to identify the binding site of TB within the biofilm. Results revealed that the extracellular ROS levels were significantly stronger than intracellular following incubation with 5 µM of TB spray and white light exposure, reaching a value 6.15 times greater than that of intracellular ROS (Supplementary Figures S16, 17). This supports the hypothesis that TB predominantly binds to components of the biofilm. What's more, the process of transitioning from planktonic microorganisms to biofilm is divided into three phases: the first phase is aggregation and attachment, the second phase is growth and accumulation, and the third phase is disaggregation and detachment [81]. Biofilms serve as a continuous reservoir for microorganisms due to the ongoing occurrence of the third phase. Detached cells can infect the host, and the persistent risk of biofilm dispersal underscores the importance of biofilm eradication as a promising therapeutic intervention. We next evaluated the efficacy of TB spray against mature biofilm. CLSM images clearly showed that the confocal dishes without TB or white light treatment were covered with a dense, viable biofilm (Figures 4A, B). As shown in Figure 4C, a large number of fungi died after being treated with TB with light (80 mW cm^−2^). Immediately afterward, a large portion of cytoplasmic leakage was observed (Figure 4D). As EPS plays important roles in cell adhesion, cell signaling, ligand binding, and as a carbon source, the different component changes were then studied [58]. It was provenowns that eDNA contributes a lot to aggregate formation [82]. Meanwhile, eDNA is also responsible for the formation and stability of the biofilm and may be a reservoir of genes for horizontal gene transfer [83]. The origin of eDNA seems to be genomic DNA and mostly lysis‐related [84], suggesting that the gDNA was extracted and selected as the substrate after various treatments to investigate the mechanism of biofilm disruption by TB [85]. TB‐mediated PDI significantly degraded genomic DNA (gDNA), as evidenced by the markedly reduced band intensity compared to the control groups (Figure 4E). Given TB's specific targeting of polysaccharides, we investigated its effect on insoluble polysaccharides in *C. auris* biofilms. As shown in Figure 4F, when *C. auris* biofilms are treated with TB under white light irradiation, the number of water‐insoluble polysaccharides was reduced, presumably because the ROS produced by TB broke the long chains of insoluble polysaccharides, which in turn may have been converted to soluble polysaccharides. The BME plays a crucial role in maintaining drug resistance and structural stability [58]. Microorganisms in deeper, quiescent regions of biofilm often experience nutrient and oxygen limitation, leading to slow metabolism, anaerobic activity, and the formation of an acidic BME. [86] *C. auris* and other pathogens embedded within EPS and produce highly acidic microenvironments with pH values as low as 4.5 [87]. As depicted in Supplementary Figure S18, we demonstrated a significant decrease in pH within the biofilm region compared to the acidic supernatant. This acidic BME may enhance the antibiofilm activity of TB, owing to its pH‐responsive AIE characteristics. These persistent microbial communities exhibit strong resistance to conventional antibiotics [88]. In addition, the hypoxic conditions within biofilm stimulate the production of reducing agents such as L‐Glutathione (GSH), which further impairs the activity of the antibiotics that enter the microorganisms [89]. Although there was no evident change in the levels of GSH (Supplementary Figure S19), the content of H_2_O_2_ increased significantly after TB with light treatment, demonstrating that TB disrupted the H_2_O_2_ balance of the biofilm, thus impairing the biofilm (Figure 4G). In summary, TB acts as a pH/polysaccharide dual response system, effectively disrupting the biofilm microenvironment through ROS generation and leading to comprehensive biofilm elimination (Figure 4H).

### 2.5 TB Spray‐Mediated PDI for Disinfections on Clinical High‐Touch Surfaces

**FIGURE 5:**
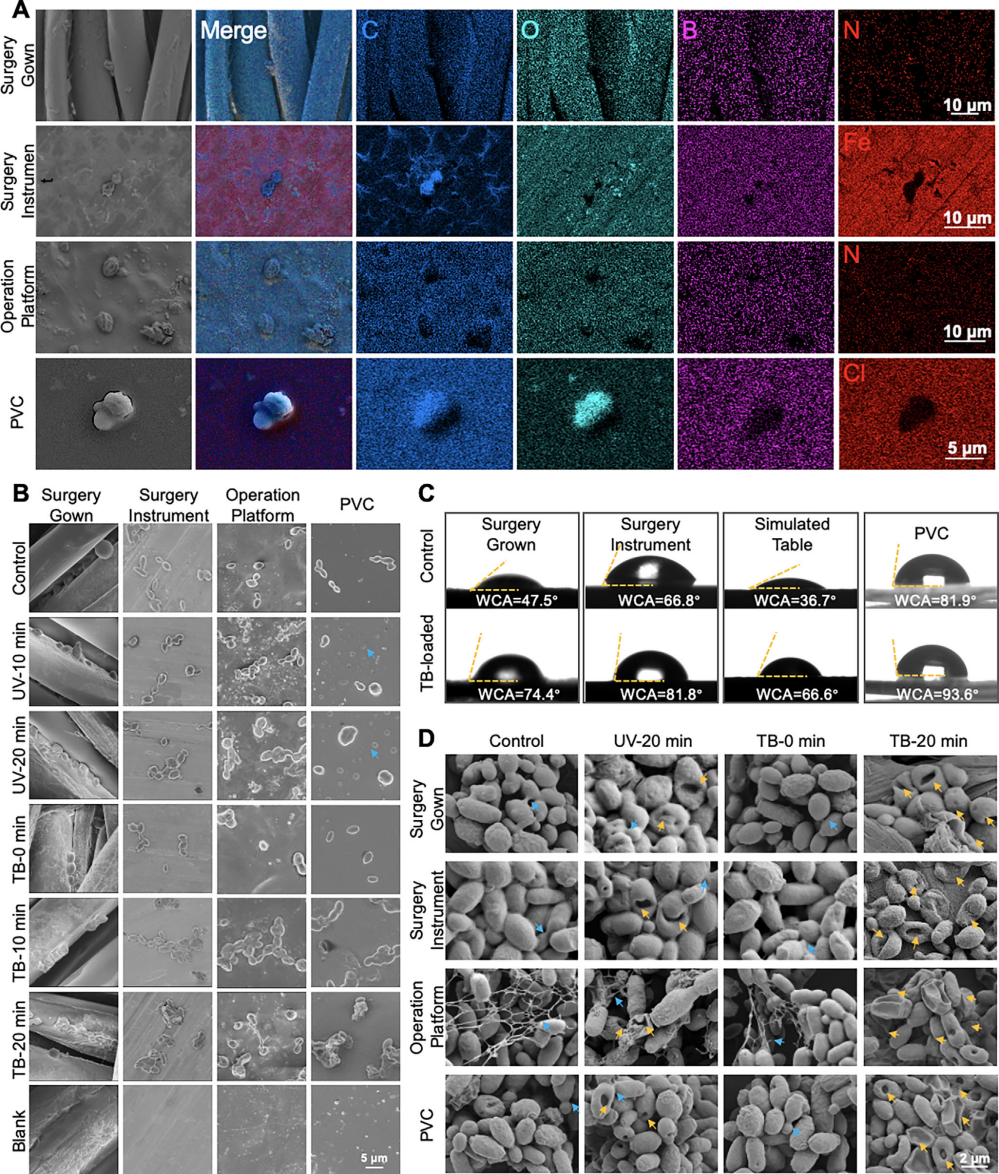
Photodynamic disinfection of clinical high‐touch surfaces of TB spray. (A) Element mapping of TB on ICU‐associated high‐touch surfaces, including surgery gowns, surgical instruments, operation platforms, and PVC (Scale bar: 10 µm and 5 µm), (B) Representative FESEM images of *C. auris* on diverse TB‐coated material (surgery gown, surgical instruments, operation platforms, and PVC) after treatment with TB spray (80 mW cm^−2^) and UV (200 µW cm^−2^) for 10 min and 20 min. Bare materials without *C. auris*, bare materials with *C. auris*, and TB‐coated materials without *C. auris* as controls (Scale bar: 5 µm). Blue arrows represent the small bubble‐like patterns formed in the PVC material following UV irradiation, (C) The static WCA comparisons of the four bare materials and TB‐coated materials. The WCAs were measured by dropping 5 µL of distilled water onto the materials, and the images were captured 1.0 s after the water was dropped, and (D) Representative FESEM images of *C. auris* biofilms on TB spray‐coated materials (surgery grown, surgical instruments, operation platform, and PVC) treated with 10 µM TB spray in the dark or irradiated with white light (80 mW cm^−2^) or UV (200 µW cm^−2^) for 20 min (Scale bar: 2 µm). Yellow arrows represent broken fungi, and blue arrows represent EPS.

In ICUs, common inanimate surfaces can become contaminated, leading to cross‐transmission of pathogens among patients with serious illness [90, 91]. Such contamination can occur through the transmission from the hands of healthcare workers or direct contact with the immediate environment. Once contaminated, these surfaces act as reservoirs for pathogenic organisms, increasing the risk of infection. As an emerging pathogenic fungus in the ICU, *C. auris* can survive and reproduce in extreme conditions due to its resistance to high temperatures and salinity [92]. and persist for extended periods on various surfaces, including wet and dry surfaces, metals, beds, and contaminated linens [93]. To take advantage of the potent biofilm eradication properties of TB, we functionalized high‐touch ICU surfaces, such as surgical gowns, instruments, operating tables, and PVC, with TB spray, endowing them with self‐sterilizing capabilities. Figure 5A demonstrates the successful loading of TB spray onto these materials. Elemental mapping confirmed the uniform distribution of B atoms (from TB) across different material surfaces and under fungal cells, similar to Fe atoms but distinct from C atoms. To assess the PDI ability of the TB‐coated materials, we then conducted experiments with light irradiation at 80 mW cm^−2^ for 10 and 20 min. UV radiation is commonly used for disinfection in hospitals; therefore, we exposed materials to UV light at 200 µW cm^−2^, a commonly used sterilization intensity, for the same duration as the control. The results, shown in Figure 5B and Figure S20, indicate that the TB‐coated materials exhibit significantly improved fungicidal ability compared to UV treatment. In addition, the PDI effect of TB‐coated surgical instruments was particularly effective, probably due to the lower specific heat capacity of titanium, and the additional elevated temperature under white light combined to act as a sterilizing agent. Notably, UV irradiation induced visible surface alteration in PVC, forming bubble‐like structures (blue arrows), likely resulting from photodecomposition and release of volatile gases [94]. Prolonged UV exposure is known to reduce PVC flexibility and usability. However, no significant difference was observed in the surface morphology of TB‐coated PVC, indicating that the presence of TB preserves the properties of PVC during disinfection, thereby extending its lifespan. Additionally, it usually takes a few hours for UV sterilization. Exposure to UV irradiation is a leading cause of cataracts and effects on the skin stratum corneum. The UV irradiation can further affect the skin of operators and patients, damage DNA, and cause permanent genetic mutations [95, 96]. Besides, when a patient is hospitalized, UV disinfection cannot be performed simultaneously. These results demonstrated the potential of employing TB to prevent the spread of infectious fungi in ICU‐acquired infections.

The first step of the biofilm formation process is aggregation and attachment, and it is crucial in preventing biofilm formation, as this step is often reversible [97]. Thus, reducing initial fungal adhesion can effectively discourage biofilm maturation. In addition to the investigated hydrophobic interactions between fungi (Figure 3F), the adhesion of *C. auris* and abiotic surfaces (Figure 3G), and the content of extracellular insoluble polysaccharides (Figure 4F), the hydrophobicity of abiotic surfaces is another important manifestation of adhesion. A hydrophobic surface or modifying hydrophilic functional groups could hinder microorganisms’ adhesion. The nature of surface hydrophobicity is closely linked with the water contact angle (WCA) [98]. Therefore, we also measured the WCA values of various materials before and after TB spray functionalization. A higher WCA means a stronger waterproof property of the materials. Notably, PVC changed from hydrophilic to hydrophobic after TB spray treatment (Figure 5C). Moreover, all TB‐coated materials exhibited a significant increase in contact angles, with the most pronounced change observed in surgical gowns and simulated tables, showing an increase of approximately 30°. The enhanced hydrophobic interactions on high‐touch surfaces, together with increased intercellular hydrophobicity of *C. auris* and reduced insoluble polysaccharides, collectively lead to diminished adhesion of *C. auris* to these surfaces. As a result, microbial persistence on ICU surfaces is shortened, thereby lowering the risk of infection. These findings are consistent with the results of the previous studies on *C. auris* hydrophobicity and distribution frequency [69].

Biofilm detection in the ICUs includes fungal and bacterial attachments on various surfaces such as chairs, nurses' station sinks, curtains, glove compartments, nightstands, and lockers [10]. Topographical features of the attached surfaces could alter microbial adherence [99]. We evaluated the efficacy of TB spray against biofilm on different materials. As shown in Figure 5D and Supplementary Figure S21, biofilm morphology varied across surfaces: thick and fiber‐aligned on surgery gowns, dense and homogeneous on the surgery instrument, and irregular and loose on the operating table and PVC. UV treatment showed limited biofilm eradication, whereas TB spray‐mediated PDI resulted in increased fungi rupture (yellow arrows), more lysates, and reduced EPS (blue arrows). Specifically, in the operating table group, an ordered spider web‐like structure of extracellular insoluble polysaccharides was observed, likely influenced by the non‐mono‐component properties of the operating table, which change biofilm morphology. Wang et al. have reported that an AIE molecule can inactivate coronaviruses on fabrics, which focuses on personal protective equipment (PPE) for various fiber products that are accessible in daily life [100], whereas our study looks at the lethal pathogenic fungus on various accessible surfaces in ICU wards, surgical gowns, stainless steel, operating tables, PVC, and so on. It has been reported that there is a positive correlation between surface roughness and bacterial/fungal attachment. Rough surfaces provide more and deeper areas for the attachment of bacteria and fungi, which results in difficult complete eradication of the pathogens [101]. Besides, bacteria and fungi are subjected to reduced shear force during wiping, as during cleaning [102]. In contrast, TB spray, as a new disinfectant, offers a simple, convenient, and effective light irradiation method instead of wiping to achieve excellent disinfection, even on rough surfaces like surgical gowns, hospital bed sheets, and linens.

### 2.6 TB Spray‐Mediated PDI Prevents VAP

Besides infections transmitted via high‐touch surfaces, VAP, bloodstream infection (BSI), and catheter‐related infection are among the most frequently reported HAIs, causing a more direct threat, especially for severe or critical cases [103]. Mechanically ventilated ICU patients face elevated infection risks from tracheostomies, reintubations, and central lines, which mediate *C. auris* transmission and VAP onset, causing most ICU‐acquired infections [57]. Despite progress in antimicrobial therapy, improved ICU guidelines, and better supportive care modalities, VAPs remain the leading causes of morbidity and mortality [104]. Improper extubation, intubation, and a prolonged period of ventilator dependence are all potential risks of drug‐resistant pathogen infections. Common clinical disinfection methods, such as UV and ultra‐high temperature sterilization, are unable to disinfect indwelling catheters again, which can easily lead to chronic incurable infection. Microbial biofilms form on implanted endotracheal tubes (ETTs) in VAP patients, shielding against host immunity and causing drug‐resistant chronic infections. Within 7 days of insertion, over 90% of ETTs are found to have biofilms, with the inner layer of the ETT providing an optimal growth medium: suitable temperature and high humidity, along with a continuous flow of nutrients in the form of mucus, cellular debris, and blood from the lower respiratory tract, facilitating pathogenic growth. However, the removal of accretion and biofilm from the ETT may only reduce the duration of mechanical ventilation and the incidence of pneumonia. Bacteria and fungi are firmly attached to the ETT by the surrounding protective insoluble polysaccharides, and the resulting biofilm is difficult to eliminate from the interior of the ETT by mechanical debridement or ultrasound at present. In this environment, the ETT could be considered an incubator for drug‐resistant species, and bacteria and fungi would continuously reach the distal airways through mechanical ventilation [105]. To make things worse, biofilms located on ETT are inaccessible to the immune system, unaffected by systemic antibiotic administration, and can only be eliminated by in vitro sterilization.

**FIGURE 6:**
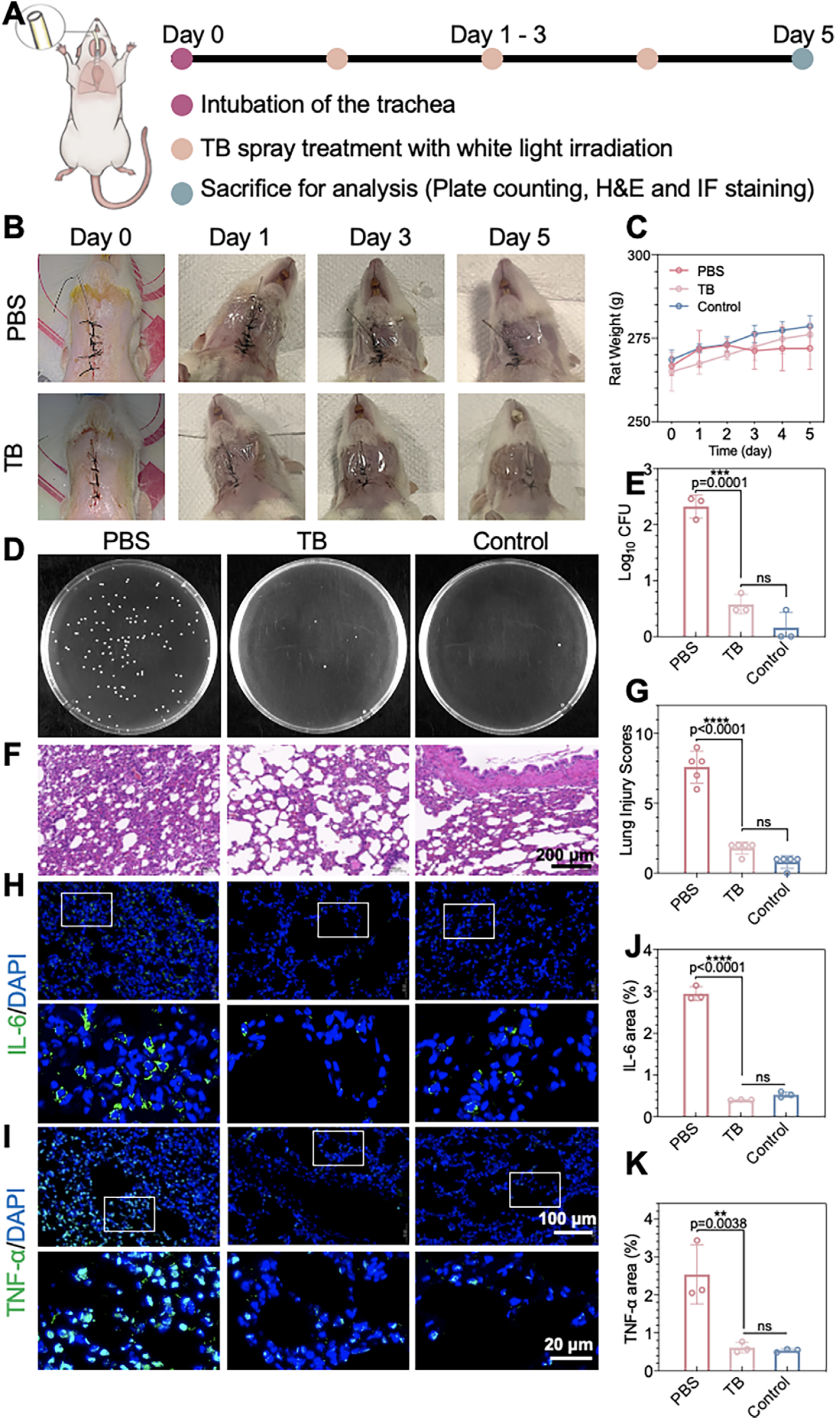
Sterilization effect of TB spray on biofilm‐attached ETTs in vivo. (A) Scheme of TB spray on rats intubated with biofilm‐attached ETTs followed by persistent white light irradiation (80 mW cm^−2^), (B) Respective photographs of the rats' necks in different groups (*n* = 3), (C) Body weight monitoring of rats intubated with biofilm‐attached ETTs after being treated with 30 µL of PBS solution containing 30 µM TB spray or 30 µL of PBS. Data represent mean ± SD (*n* = 3), (D) Respective agar plate images of *C. auris* in lung tissue in different groups, (E) The corresponding colony forming unit (CFU) in the lung culture in different groups. F) Images of HE‐stained rat lung slices (Scale bar: 200 µm) in different groups (Scale bar: 100 µm). (G) The corresponding lung injury scores in different groups. Data represent mean ± SD (*n* = 3). (H–I) Respective immunofluorescence staining images of IL‐6 (H) and TNF‐α (I) in lung tissues after different treatments on Day 5. (Scale bar: 100 µm and 20 µm) (J–K) Percentages of IL‐6 (J) and TNF‐α (K) areas in lung tissues (*n* = 3). Data are presented as the mean ± SD. Data are presented as the mean ± SD of at least 3 replicates. Statistical significance between every two groups was calculated via one‐way ANOVA. ****p < 0.0001, ***p < 0.05, **p < 0.01, and *p < 0.001; **Abbreviation**: ns, not significant.

Capitalizing on the polysaccharide‐targeting ability of TB, we further explored its role in the later stages of biofilm formation in ETT to verify whether TB could have a sterilizing effect in vivo. Using a 12‐week‐old rat model, ETT biofilm infection experiments were conducted (Figure 6A). Respective photographs of the rats' necks were documented by a digital camera (Figure 6B). Rats intubated with biofilm‐attached ETT were randomly divided into two groups and received the following treatments: (1) PBS and (2) TB spray with light irradiation. Moreover, healthy rats without ETT were used as controls. As shown in Figure 6C, in the PBS group, significant weight loss was observed by the third day, potentially attributed to the fungi dissemination from the biofilm entering the lungs and causing an infection. Conversely, the weight of the TB spray‐treated group remained unchanged, indicating a potential infection prevention. As shown in Figures 6D and E, the rats in the PBS group were significantly more colonized in the lung lavage fluid, which indicated that biofilm fragments adhering to the ETT disperse into the lungs and cause *C. auris* lung infections. Histological analysis in Figure 6F revealed narrower alveolar intervals in the TB spray group compared to the PBS group. No significant difference in lung morphology was found between the TB spray group and the control group, suggesting the disinfecting efficacy of TB on PVC catheters. Lung injury scores demonstrated a lower degree of damage in the TB spray group compared to the PBS group (Figure 6G). IL‐6 is a commonly detected cytokine and a key inflammatory marker induced during pneumonia [106]. The β‐glucan component present in the *C. auris* cell wall has been demonstrated to elicit an immune response and activate the release of inflammatory markers such as IL‐1 and TNF‐α [107]. Immunofluorescence staining showed that the lungs in the PBS treatment group had a significant inflammatory response due to infection with a large number of *C. auris* and a large expression of the pneumonia‐related inflammatory factors IL‐6 and TNF‐α. In contrast, the expression of IL‐6 and TNF‐α was lower in the TB spray treatment group and was not significantly different from the control group (Figure 6H–K). These in vivo results suggested that the effective disinfection of intubated medical devices by TB prevented the invasion of *C. auris* into the lung, implying this type of disinfection may apply to more generalized and extensive disinfection of ICU‐associated high‐touch surfaces.

### 2.7 Mechanism Study of the Antifungal Effect of TB Spray by Multi‐Omics Analysis

#### 2.7.1 Transcriptomics Analysis

**FIGURE 7:**
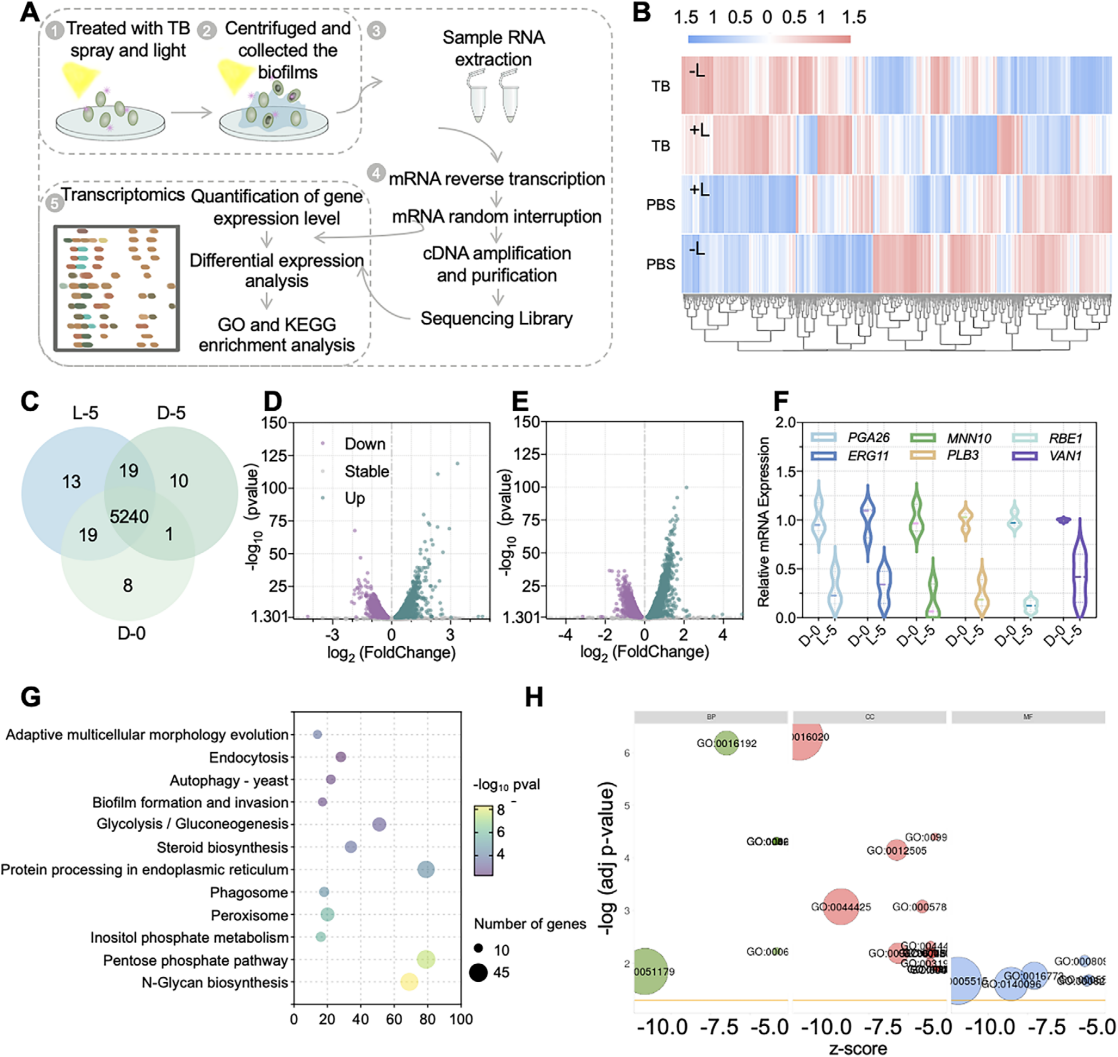
The molecular mechanism of TB spray against biofilm formation. (A) Schematic diagram of transcriptomics to study the anti‐biofilm mechanism of TB spray under white light irradiation, (B) The hierarchical clustering heat map of DEGs after different treatments, (C) The Venn diagram displays the coexpression genes of the three groups, (D–E) The bubble plots of TB spray with light versus PBS without light (D) and TB spray without light versus PBS without light, (E) Green dots represent up‐regulated genes, whereas purple dots represent down‐regulated genes (|log_2_FoldChange| ≥ 0, ‐log_10_pvalue ≥ 1.301), (F) RT‐qPCR results of biofilm regulation and basic metabolism, including *PGA26*, *MNN10*, *RBE1*, *ERG11*, *PLB3*, and *VAN1*, and (G–H) The bubble plots of the top DEGs enriched in the KEGG (G) and GO (H) pathways.

Biofilm formation is a highly dynamic process tightly regulated by genetic programs. Transcriptomics is ideal for detecting rapid changes in gene expression profiles [108]. To further explore the mechanism of action of TB spray‐based photodynamic disinfection, we performed gene transcriptome sequencing to analyze the whole genetic expression of *C. auris* under different treatments (Figure 7A and Supplementary Figures S22 and 23). Differentially expressed genes (DEGs) were identified by |log_2_FoldChange| ≥ 0 and *p*‐value ≤ 0.05. The hierarchical clustering heat map of DEGs after different treatments is shown in Figure 7B. Gene expression profiles of cells treated with PBS without light irradiation, PBS with light irradiation, TB spray without light irradiation, and TB spray with light irradiation were analyzed. The DEGs of three of these groups are displayed in the Venn diagram (Figure 7C). The volcano plots of DEGs are in Figures 7D, E, and Supplementary Figure S24. Specifically, 251 genes were up‐regulated and 91 genes were down‐regulated after treatment of *C. auris* with TB spray under white light irradiation compared to the control group. To further illustrate the effects of TB spray with white light on biofilm and metabolism, heatmap analysis of enriched biofilm and basic metabolite‐related DEGs was performed (Supplementary Figure S25). One gene of interest is *MAE1*, which encodes a malic enzyme involved in the fungal redox state and the pyruvate metabolic pathway [109]. The overexpression of *MAE1* in the TB spray with the white light group suggests that ROS alters the redox homeostasis. Furthermore, PDI significantly affected the biofilm formation and adhesion of *C. auris*. We observed significant down‐regulation of the *CYR1* gene, which regulates the QS involved in biofilm formation, as well as down‐regulation of genes (*RBE1*/*VAN1*/*PLB3*/*PGA26/IFF9/HYR1/ACE2/SNF5/MNN10*) that promote biofilm formation, adhesions, and invasion [110]. Sticky *C. auris* encodes a vast range of adhesion, including but not limited to *PGA26/IFF9/HYR1/ACE2/SNF5*. The *IFF*/*HYR* adhesion family has been shown to mediate cellular and mucosal/abiotic substrate adhesion as well as cell‐cell cohesion and regulation of the mycelial development, respectively [111]. *PGA26* has been studied for its potential role within cellular adhesion, and *ACE2/SNF5* was required for adherence to polystyrene surfaces [112]. Additionally, *RBE1* is strongly associated with *Candida* pathogenicity [113, 114], and *VAN1*/*PLB3* and *MNN10* mediated the outer cell wall mannan [115], lysophospholipases, and α‐1,6‐mannosyltransferase, respectively, to regulate biofilm invasiveness [116]. Biofilms are believed to play a crucial role in the establishment of drug‐resistant strains of microorganisms in the environment. This is due to the high cell density of coexisting antibiotic‐resistant and sensitive microbes in mixed biofilms, coupled with accumulated mobile genetic elements, leading to efficient horizontal gene transfer of antibiotic resistance genes [113, 117]. To our surprise, *Erg11* and *FCR1* showed significant down‐regulation and up‐regulation, respectively. In particular, an *Erg11* mutation contributes to azole resistance in *Erg11* [118], and *FCR1* negatively modulates *Candida* resistance to FCZ, ketoconazole, brefeldin A, fluphenazine, and itraconazole drugs [33]. Interestingly, the expression of the *FCR1* gene was already up‐regulated with TB spray treatment alone, but the addition of ROS generated by light further increased its expression. This suggests that there may be an additive or synergistic effect of TB and ROS on the up‐regulation of *FCR1* expression. To further validate that TB does not induce drug resistance, *C. auris* was subjected to 10 cycles of TB‐mediated PDI treatment to investigate the potential development of new resistance [60]. As shown in Supplementary Figures S26A–C, TB‐mediated PDI maintained its antifungal activity even after 10 treatment cycles. Furthermore, the expression level of *ERG11* hardly increased (Supplementary Figure S26d), and *C. auris* remained highly susceptible to TB‐mediated PDI at 5 µM, indicating no induction of resistance. qRT‐PCR (Figure 7F) further verified the expression of the above typical genes, which gave similar results to the transcriptome DEGs by using the *GPD1* gene (encoding Glycerol‐3‐Phosphate Dehydrogenase 1) as an internal reference. The primer sequences used are shown in Supplementary Table S1, Kyoto Encyclopedia of Gene Genomes (KEGG), and Gene Ontology (GO) classification annotation analysis revealed that PDI‐induced cell death in *C. auris* treated with TB spray and white light may be primarily caused by the biosynthesis of secondary metabolites (Figures 7G, H, and Supplementary Table S2). In addition, KEGG also enriched the adaptive multicellular morphology evolution pathway, which is mainly concentrated in cell wall and integrity (*Van1*, *Vrg4*, *Chs1*), cytoskeleton and polarity (*Apl2*, *Apl4, Apm1*, *Arc19*, *Pan1*, *Laa1)*, and the Regulation of *Ace2* Morphogenesis (RAM) pathway (*Ace2*), all of which showed a down‐regulation trend.

Moreover, we analyzed the top 200 down‐regulated genes of TB spray with light versus PBS without light by protein‐protein interaction networks (PPIs), to thoroughly explore the roles of these differentially varied genes in various biological processes. We employed the Markov Cluster method to segment the entire network into distinct clusters in Supplementary Figure S27 and Supplementary Table S3. Due to the lack of protein studies on *C. auris* in the database, more uncharacterized proteins appeared in the PPIs and these proteins without interactions were manually removed. These established intensive clusters are essential for understanding the anti‐biofilm mechanism, encompassing processes such as regulation of mitotic cell cycle phase transition and cell division, oxidative stress, TCS, ergosterol biosynthesis, multidrug efflux, carboxylic acid metabolism, and the QS system. The combined therapy of TB spray and white light significantly induces cell death mainly by affecting the biosynthesis of secondary metabolites (ergosterol, etc.), oxidative stress, and inhibition of cell division. It also prevents the formation of intact biofilms by regulating cellular communication within biofilms and inhibiting genes such as the TCS, multidrug efflux and QS, biofilm invasion (*VAN1*, *PLB3*, *MNN1*), and adhesion (*PGA26, IFF9, HYR1, ACE2, SNF5*).

#### 2.7.2 Untargeted Metabolomics Analysis

**FIGURE 8:**
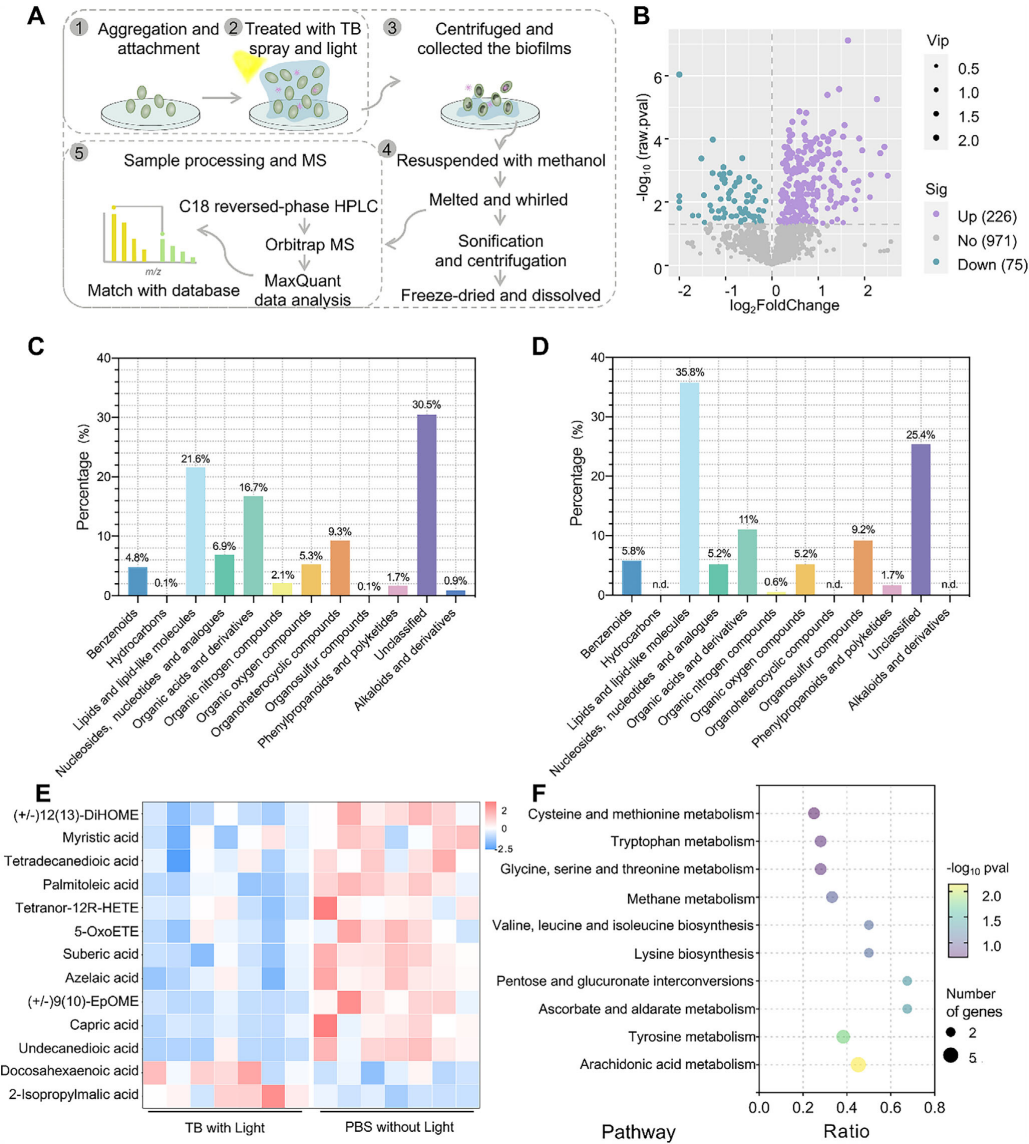
The molecular mechanism of TB spray eradicated mature biofilm. (A) Schematic diagram of metabolomics to study the anti‐biofilm mechanism of TB spray under white light irradiation, (B) The volcano plot of PBS without light versus TB spray with light. Green dots represent down‐regulated genes, whereas purple dots represent up‐regulated genes (|log_2_FoldChange| ≥ 0, ‐log_10_pvalue ≥ 1.301), (C) The classification of all detected substances from the metabolic analysis, (D) The classification of DAMs from the metabolic analysis, (E) The hierarchical clustering heat map of differentially altered fatty acids and conjugates after different treatments, and (F) The bubble plots of the top DEGs enriched in the KEGG pathway.

Biofilms have been defined as ‘aggregates of microorganisms in which cells are frequently embedded in a self‐produced matrix of EPS that are adherent to each other and/or a surface’ [58]. Their complexity is reflected in both species composition and phenotypic diversity [119]. In addition, the acidic and hypoxic conditions within biofilms often lead to microbial persistence, impairing the efficacy of antibiotics that typically target metabolic activity [120]. Microorganisms within mature biofilms often exhibit slow metabolism. Metabolomics provides a real‐time “snapshot” of the functional state of the biofilm [121]. To explore metabolic variations in *C. auris* BME following TB spray‐mediated PDI, the expression levels of metabolites in 14 samples (7 samples per group) were evaluated and analyzed in Figure 8A. The partial least squares‐discriminant analysis (PLS‐DA) results showed clear separation between the control and treatment groups (Supplementary Figures S28, 29). Among 1272 metabolites detected, 301 showed significant changes after treatment: 226 were up‐regulated and 75 down‐regulated in the TB spray with the light group compared to PBS in the dark (Figure 8B and Supplementary Figures S30, 31). Differentially altered metabolites (DAMs) were identified by |log_2_FoldChange| ≥ 0 and *p*‐value ≤ 0.05, along with variable importance in projection (VIP) scores ≥ 1. The classification of all detected metabolites is shown in Figure 8C, with differential metabolites categorized in Figure 8D. Lipids and lipid‐like molecules represented the class with the highest number of significantly altered metabolites. Among 64 differentially expressed lipids, 58 were down‐regulated and only 6 up‐regulated, the latter primarily comprising saturated lipid‐like molecules (Supplementary Figure S32). Further analysis of lipid subclasses revealed that 84.6% of detected fatty acids and conjugates were significantly down‐regulated (Supplementary Figure S33). Although eukaryotic organisms such as fungi have well‐established antioxidant mechanisms, the widespread down‐regulation of a large number of fatty acids suggests collapse of these systems in *C. auris* following TB spray application, leading to lipid peroxidation (Figure 8E) [122]. Consistent with this, we observed a significant increase in malondialdehyde (MDA) (a toxic byproduct of lipid peroxidation) in the TB‐treated biofilms (Supplementary Figure S34). In addition, down‐regulated lipids and lipid‐like molecules were enriched in arachidonic acid metabolism. Since arachidonic acid, the sole carbon source for growth and morphogenesis during *Candida* infection, is also known to affect *C. auris* and *P. aeruginosa* biofilm interactions [123], TB spray could affect *C. auris* growth by downregulating arachidonic acid expression. In addition to lipid changes, KEGG pathways analysis showed significant down‐regulation in the synthesis and metabolism of essential amino acids (including lysine, glycine, serine, threonine, tryptophan, cysteine, and methionine metabolism). This implies a severe inhibition of overall fungal growth and viability within the biofilm. Even if residual biofilms remain, their metabolic activity and capacity for recurrence are substantially compromised (Figure 8F and Supplementary Figure S35).

In summary, these observations by employing transcriptomics and metabolomics analysis data highlight the importance of identifying *C. auris* biofilm characteristics after TB spray‐mediated‐PDI on genetic and molecular levels. TB spray‐mediated PDI prevents the formation of intact biofilms through down‐regulating gene expression such as the TCS, multidrug efflux (*ERG11, FCR1*), oxidative stress (*MAE*), QS, biofilm invasion (*VAN1*, *PLB3*, *MNN1*), and adhesion (*PGA26, IFF9, HYR1, ACE2, SNF5*). This visible‐light‐induced therapy also efficiently eliminates biofilms by promoting lipid peroxidation of mature biofilms, inhibiting the biosynthesis and metabolism of essential amino acids, and suppressing the recurrence of biofilms.

### 2.8 Biocompatibility Evaluation of TB Spray

**FIGURE 9:**
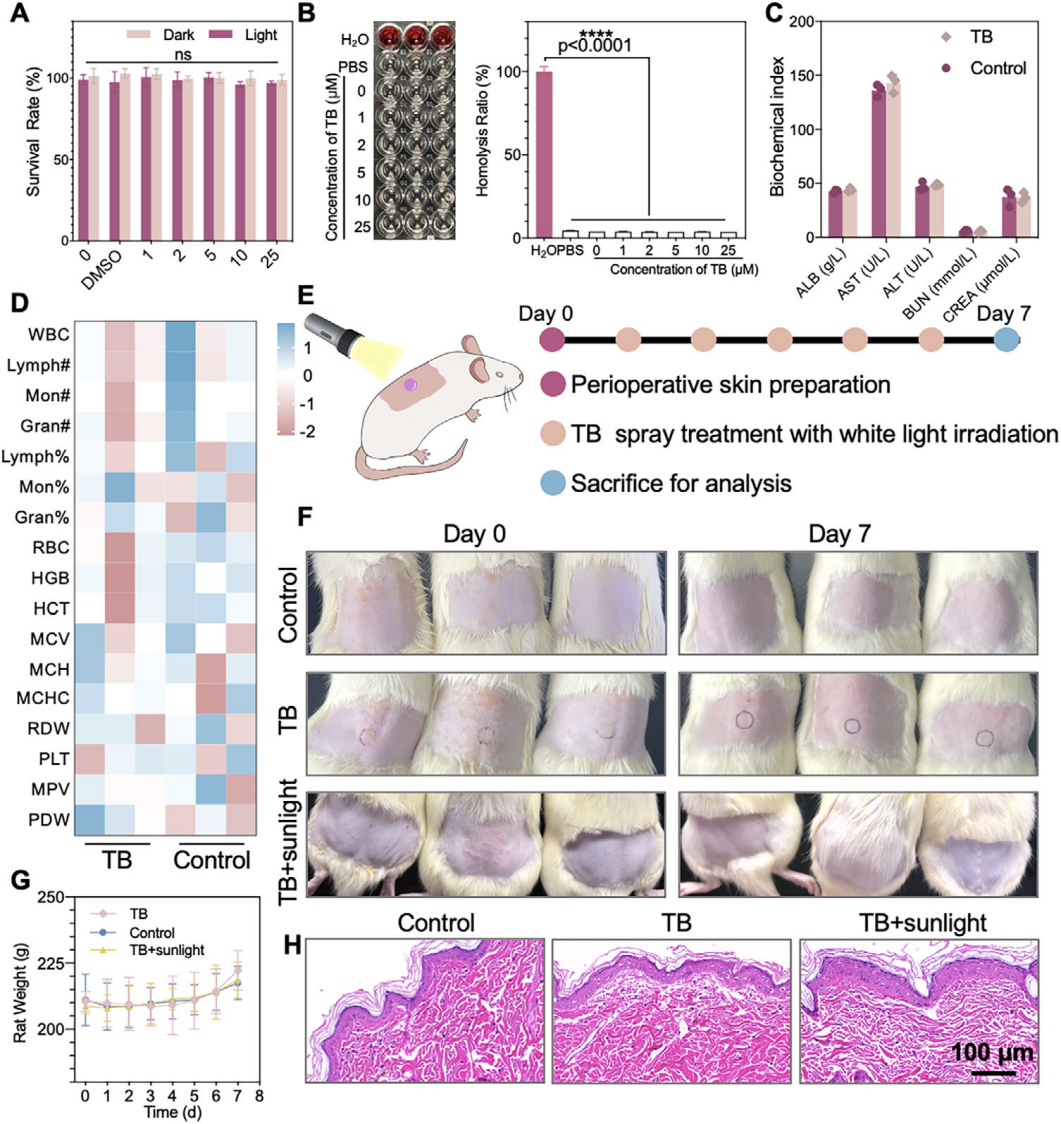
Biocompatibility evaluation of TB spray. (A) Cell viability of mammalian normal cell COS‐7 after coincubation with different doses of TB spray for 20 min white light eradication (CCK‐8 as the indicator), (B) Evaluation of hemolysis induced by water, PBS, and TB spray at different concentrations, (C) Blood biochemical data of healthy rat after intravenous injection of 30 µL of TB spray (30 µM in PBS) or 30 µL of PBS, (D) Heat map of hematologic analysis of healthy rat 3 days after intravenous injection of 30 µL of TB spray (30 µM in PBS) or 30 µL of PBS, (E) Scheme of TB spray on rat skin followed by persistent white light irradiation (80 mW cm^−2^), (F) Images of rat skins after daily smearing of PBS or 30 µM TB spray for different days, (G) Body weight monitoring of healthy rat after the skin was treated with 30 µL of PBS solution containing 30 µM TB spray or 30 µL of PBS, and (H) Images of HE‐stained rat skin slices after TB spray treatment followed by white light irradiation (80 mW cm^−2^) and sunlight for different time durations (Scale bar: 100 µm). Data are presented as the mean ± SD of at least 3 replicates. Statistical significance between every two groups was calculated via one‐way ANOVA. ****p < 0.0001, ***p < 0.001, **p < 0.01, and *p < 0.05; **Abbreviation**: ns, not significant.

Currently, antifungal agents represented by three classes of azoles, polyenes, and echinocandins are the first‐line method [124]. Nevertheless, as a result of the similarity between fungi and humans, potent antifungal agents are prone to having potential adverse events that may compromise safety or adherence. Subsequently, we systematically evaluated the in vivo and in vitro toxicity and biocompatibility of TB spray. First, the toxicity of TB to mammalian normal cell COS‐7 cells was measured using the CCK‐8 assay. Cell viability was assessed after co‐incubation with different concentrations of TB followed by 80 mW cm^−2^ illumination for 20 min. The results showed cell viability remained unaffected at all tested drug concentrations, demonstrating promising biocompatibility between TB and normal mammalian cells (Figure 9A). Additionally, Live/Dead staining also demonstrated that even high concentrations of TB (up to 25 µM) showed excellent biocompatibility and had no obvious change in the morphology of COS‐7 (Supplementary Figure S36). In addition, HaCaT cells also showed similar results (Supplementary Figure S37). Next, the hemolysis experiment was conducted, in which different concentrations of TB were incubated with rat erythrocytes for 30 min to observe whether there was obvious hemolysis. The results showed that no obvious hemolysis was observed even when the concentration of TB was as high as 25 µM, which indicated excellent compatibility between TB and blood (Figure 9B). To evaluate the in vivo toxicity of TB spray, healthy rat were injected intravenously with a high concentration of TB (30 µL, 30 µM) and then analyzed for blood biochemical indices and organ damage. The change of weight was recorded every day in Supplementary Figure S38. Figure 9C shows biochemical markers (albumin, ALB; aspartate aminotransferase, AST; alanine aminotransferase, ALT; blood urea nitrogen, BUN; and urea nitrogen, UREA) remained stable post‐TB treatment versus PBS controls, indicating no hepatorenal toxicity in treated rat. In addition, hemoglobin‐eosin (HE) staining results (Figure S39) and hematologic function analyses (Figure 9D and Supplementary Figure S40) showed that TB spray treatment did not cause significant pathological changes in various organs of healthy rat. Considering that TB spray has the potential to be sprayed for controlling HAIs, the toxicity of normal skin was also included in the biocompatibility evaluation system. A high concentration of TB spray (20 µL, 30 µM) with white light irradiation (80 mW cm^−2^) was applied to the skin surface of normal mice once a day (Figure 9E and Supplementary Table S4). Rats treated with TB spray with daylight exposure were also observed. The skin appearance was examined every day, and the HE staining of the skin was performed after 5 days of treatment (Figure 9F). As demonstrated in Figure 9G, the body weight of the rats increased steadily during the administration period. As shown in Figure 9H, according to the H&E staining results observed under the light microscope, the cuticle of the skin treated with TB spray showed a homogeneous banding on the active epidermis. It was tightly attached to the epidermis without cuticle exfoliation or dissociation, both in the TB spray with the white light group and with the daylight group, which was not significantly different from that of normal skin without any treatment. These results indicate that TB spray is a safe biomaterial that can be applied to the skin surface and acts as a catheter follow‐up disinfectant.

## 3 Conclusion

In summary, we designed and synthesized a dual‐type AIE PS spray, the PS containing a cationic quaternary ammonium unit and a glycosyl‐targeted boronic acid moiety, characterized for the elimination of biofilm infections of *C. auris*. This AIE small molecule has a high molecular absorption rate, a wide absorption band, and excellent ROS generation efficiency, which can photodynamically eliminate *C. auris* and biofilm and cut off the transport pathway of HAIs. The biofilm penetration of TB spray is facilitated by its targeting of polysaccharides. Furthermore, the acidic BME amplifies the surface positive charge and particle size of TB, which further enhances its affinity and eradication capability with *C. auris* biofilm, as well as membrane potential homeostasis disturbance. TB achieves excellent HOMO‐LUMO isolation with low Δ*E*_S‐T_, promoting an efficient ISC process. TB combats hypoxic BME by simultaneously generating type I and type II ROS, disrupting membrane potential equilibrium, and averting recurrence. In addition, the smart AIE spray also showed excellent anti‐adhesion effects on *C. auris* biofilms at all stages of adhesion in the ICU, including cell autoaggregation, hydrophobicity, adhesion between cells and abiotic surfaces, and hydrophobicity of high‐touch surfaces. In an ICU setting, TB spray has demonstrated favorable PDI effects on high‐touch surfaces. What's more, TB spray‐mediated PDI has shown an effective prevention effect on VAP in rats, which significantly protected the lungs and prevented the damage (pulmonary edema, inflammation, etc.) in vivo. Multi‐omics analysis showed that TB spray‐mediated PDI could not only reduce virulence factors (biofilm invasion, *VAN1*, *PLB3*, *MNN1*; adhesion, *PGA26, IFF9, HYR1, ACE2, SNF5*), but also inhibit QS and TCS and reduce the activity of drug efflux pump (*ERG11, FCR1*) by regulating related genes. The simple therapy also effectively eliminates biofilms by promoting lipid peroxidation of mature biofilms, inhibiting biosynthesis and metabolism of essential amino acids, and inhibiting the recurrence of biofilms. However, a more detailed analysis of the long‐term stability of the TB spray coating under extreme conditions and any potential minimal leaching will be an essential component of future development stages on the path to clinical application. This visible‐induced strategy offers a convenient and all‐embracing approach to controlling HAIs and defending against outbreaks of fungal pathogens and holds great promise as the last line of defense in the real world.

## 4 Experimental Section

### 4.1 Computational Details

All calculations were performed using the Gaussian 16 software. The functional PBE0 and basis set 6–31G (d, p) were used for geometry optimization and frequency calculation. Geometry optimizations and vibrational frequency analyses incorporated Grimme's GD3BJ dispersion correction, proving that all structures have no imaginary frequency. The calculations were carried out in a dichloromethane solution with an SMD solvation model. Additionally, spin‐orbit coupling matrix elements were derived from time‐dependent DFT calculations executed in ORCA.

### 4.2 ROS Generation Efficiency Measurement

In the dark, 10 µM of H2DCF was well blended with 10 µM of TB in PBS. Subsequently, the resulting solution was illuminated with light for a duration between 0 and 170 s. The fluorescence intensity at 525 nm was then measured using a fluorescence spectrometer with an excitation wavelength of 488 nm.

For the monitoring of ^1^O_2_, 9,10‐anthracenediyl‐bis(methylene) dimalonic acid (ABDA) was employed, with rose bengal red (RB) serving as the control. In water, 50 µM of ABDA was mixed with either 5 µM of TB or 5 µM of RB and then irradiated under white light for 0 to 18 min. The decay rate was calculated by recording the absorbance of ABDA at 378 nm for various light exposure times.

To detect the generation of hydroxyl radicals (•OH), hydroxyphenyl fluorescein (HPF) was utilized as an indicator. A 5 µM solution of HPF was added to a PBS solution containing 10 µM of TB and then exposed to white light. The fluorescence intensity at 515 nm was recorded to evaluate the efficiency of •OH production.

### 4.3 Cell and Fungal Strain Culture

*C. auris* was cultivated in a PDB liquid culture medium at 28°C for one night. The fungal concentration was ascertained by measuring the optical density at 600 nm (*OD*_600_). The *C. auris* strain CCTCC PY 2019061 was generously donated by Prof. Chao Shen from the China Center for Type Culture Collection.

COS‐7 cells, sourced from the China Center for Type Culture Collection, were cultured in DMEM. The culture medium was supplemented with 10% heat‐inactivated FBS and 1% antibiotic‐antimycotic (P/S). The cells were incubated in a 5% CO_2_ humidified environment at 37°C.

### 4.4 Biofilm Culture

For the growth of biofilms, confocal dishes and plates were pretreated with FBS overnight. The following day, the FBS was removed. Yeast cells obtained from overnight cultures were washed in sterile PBS. Subsequently, the optical density at 600 nm (*OD*_600_) of the yeast cells was adjusted, and they were resuspended to 0.1 in Spider medium (comprising 10 g of nutrient broth, 10 g of mannitol, and 2 g of K_2_HPO_4_ per 1 L; the pH was 7.2 after autoclaving). Finally, the dishes and plates were incubated at 37°C for 24–36 h to facilitate the formation of biofilms.

### 4.5 Zeta Potential and Particle Size Measurements

The zeta potential and particle size of TB (5 µM, in PBS and ddH_2_O) were measured at 25°C from a minimum of three samples (maximum 100 runs each) using a Zetasizer Nano ZS instrument (Malvern, UK). The cuvettes were always rinsed twice with PBS or ddH_2_O between each measurement.

### 4.6 Photodynamic Inactivation of Planktonic Fungi

*C. auris* was cultivated overnight in YPD medium at 28°C. Subsequently, the cells were collected, diluted with PBS to an OD_600_ of 0.1, and readied for use. Then, *C. auris* was incubated with varying concentrations of TB (0, 1, 2, 5 µM) at 37°C for 15 min, followed by irradiation with or without white light (80 mW cm^−2^) for 20 min. To assess the viability of the fungi, multiple methods were employed, including fluorescent staining, plate counting, and FESEM and TEM imaging.

### 4.7 MATH Method

Characterization of hydrophobicity was carried out as described in previous reports. Briefly, *C. auris* was incubated overnight at 28°C in YPD media, harvested, and diluted with PBS to an *OD*_600_ value of 0.6 before use. *C. auris* cells were treated with PBS containing 0 and 5 µM TB; the *OD*_600_ at this time was recorded as *A*_0_. Then, *C. auris* cells were irradiated with or without white light (80 mW cm^−2^, 20 min) and equal amounts of sample and equal amounts of xylene were mixed in an Eppendorf tube. After 10 min incubation at room temperature, the mixtures were vortexed at the highest speed for 90 s. After vortexing, mixtures were rested for 15 min at room temperature to allow complete phase separation. Next, the lower aqueous phase was used to measure the *OD*_600_ values, which were recorded as *A*_1_. The hydrophobic activity was calculated as (*A*_0_ − *A*_1_)/*A*_0_ × 100%.

### 4.8 Evaluation of TB on Cellular Autoaggregation Capacity

*C. auris* was incubated overnight at 28°C in YPD media, harvested, and diluted with PBS to an *OD*_600_ value of about 0.6 before use, and the *OD*_600_ of the suspension was recorded as *A*_0_. After being incubated with different concentrations of TB (0, 5 µM) at 37°C for 15 min, *C. auris* was irradiated with/without white light (80 mW cm^−2^) for 20 min. 2 mL of *C. auris* suspension was mixed thoroughly and incubated at 28°C for another 5 h. Then, 200 µL of the suspension was aspirated from the surface and kept at rest. The *OD*_600_ of the suspension was recorded as *A*_t_. The experiment was repeated three times. The capacity of cell autoaggregation (%) was calculated as follows: Auto‐aggregation (%) = (1 − *A*_t_/*A*_0_) × 100%.

### 4.9 Fungus Motility Analysis

The confocal dishes were pretreated with FBS overnight. The next day, *C. auris* cells were resuspended at *OD*_600_ = 0.1 with Spider medium. Next, TB was added to a final concentration at 0 µM and 2 µM in the confocal dish at 37°C for 15 min. After being irradiated with/without white light (80 mW cm^−2^) for 20 min, *C. auris* was immediately mixed and filmed on time‐lapse with no delay for 5 min. The Tracking plug‐in of NIS‐Elements was used to process the fungus motion movie and track the motion trajectory of individual fungi to calculate the fungus movement distances and related motion parameters for characterizing fungus motility.

### 4.10 Biofilm Imaging

Mature biofilms were formed as above. After removing the culture medium and washing twice with PBS, the biofilms were incubated with 1 mL PBS containing 5 µM TB for 30 min and then rinsed 3 times with PBS. The biofilm images were recorded with CLSM using 60× oil immersion objective lenses and processed using NIS‐Elements AR software. Excitation wavelength: 561 nm, emission wavelength: 620–720 nm.

### 4.11 Measurement of ROS in Biofilms

Mature biofilms were generated using the previously described method. The Reactive Oxygen Species Assay Kit (S0033, Beyotime) was utilized to indicate the total amount of ROS.

To detect extracellular ROS, DCFH‐DA, a commercially available compound, was first transformed into DCFH. This was achieved by combining 1 µL of a DCFH‐DA stock solution in ethanol with 49 µL of 0.01 M NaOH in water and letting the mixture stand at room temperature for 30 min. First, the biofilm was washed with PBS. Then, it was incubated in the dark at 37°C for 15 min with 100 µL of 5 µM DCFH. After that, the biofilm was washed again with PBS. Subsequently, 100 µL of either 5 µM TB or PBS was added to the biofilm, and it was incubated for an additional 15 min at 37°C. A biofilm treated with TB but not irradiated served as the control.

For the detection of intracellular ROS, the biofilm was washed with PBS and then incubated in the dark at 37°C for 15 min with 100 µL of 5 µM DCFH‐DA. After incubation, it was washed once more with PBS. Next, 100 µL of either 5 µM TB or PBS was added to the biofilm, followed by another 15‐min incubation at 37°C. Similar to the extracellular ROS detection, a non‐irradiated biofilm treated with TB was used as the control.

The fluorescence of the biofilm in confocal dishes was visualized using a CLSM. In 96‐well black plates, the fluorescence emission intensity of the mixed solutions at 525 nm (with an excitation wavelength of 488 nm and a bandwidth of 2 nm) was measured using a Molecular Devices SpectraMax i3x multimode microplate detection system.

### 4.12 In Vitro Anti‐Biofilm Assay by Live/Dead Staining

The Live & Dead Viability/Cytotoxicity Assay Kit was used to study the *C. auris* biofilm eradication effect of TB. Mature biofilms were formed as above. The biofilm was incubated with 5 µM TB and PBS for 15 min at 37°C and then irradiated with white light (80 mW cm^−2^) for 20 min or in the dark. After being washed three times with PBS, the biofilms were processed using the Live & Dead Viability/Cytotoxicity assay kit in accordance with the standard protocol. Additionally, some biofilms were prepared for FESEM imaging using the method described earlier.

### 4.13 Zeta Potential Measurements of *C. auris*

When *C. auris* entered the log phase of growth, zeta potential measurements were conducted. The *C. auris* cells were treated with 5 µM TB for 10 min. To assess the impact of TB, the zeta potential of at least 3 samples (with up to 100 runs per sample) was measured both before and after the TB treatment at 25°C. Between each measurement, the cuvettes were thoroughly rinsed twice with ddH_2_O.

### 4.14 Protein Leakage Assay

Mature biofilms were formed as above. The biofilm was incubated with 5 µM TB and PBS for 15 min at 37°C and then irradiated with white light (80 mW cm^−2^) for 20 min or in the dark. Then, the supernatants were collected, and the concentration of intracellular protein leaked from biofilms was evaluated using an enhanced BCA protein assay kit (Beyotime).

### 4.15 The Integrity of the gDNA Experiment

To study the degradation of eDNA, *C. auris* genomic DNA was extracted by a genomic DNA kit. Fungal genomic DNA (gDNA) was extracted using the VAMNE Magnetic Pathogen DNA Kit (DM202, Vazyme). After treatment with PBS (with or without white light irradiation, 80 mW cm^−2^), TB (with or without white light irradiation, 80 mW cm^−2^), gDNA was collected by centrifugation. gDNA cleavage products were finally detected by isolation on a 1.0% TAE agarose gel, stained with NA‐Red, and imaged on a gel using the FX Imaging System (Bio‐Rad, USA).

### 4.16 Statistical Analysis

Each experiment was carried out with a minimum of three replicates. Statistical analysis of all data was conducted using GraphPad Prism 8 software. ImageJ was employed to process and analyze 2D images as well as fluorescence intensities. For 3D images, NIS‐Elements AR software (Nikon) and Comstat2 were utilized for processing. Unless otherwise specified, all data are presented as the mean ± standard deviation (SD). To compare multiple groups, one‐way analysis of variance (ANOVA) was followed by Tukey's post hoc test. When comparing different groups, untreated samples served as controls to calculate significant differences, with ****p < 0.0001, ***p < 0.001, **p < 0.01, and *p < 0.05; ns, not significance.

## Author Contributions

**Xiaoyu Xu**: writing – original draft, visualization, validation, methodology, investigation, formal analysis, data curation, and conceptualization. **Ming‐Yu Wu**: validation and methodology. **Baoping Li**: validation and methodology. **Jie Li**: validation and methodology. **Siyu Chen**: validation and methodology. **Luojia Chen**: validation and methodology. **Donghu Yu**: validation and methodology. **Liupiaopiao Yang**: validation and methodology. **Ziyu Hong**: validation. **Huaqin Pan**: validation. **Wei Xiang**: review. **Shun Feng**: validation. **Lianrong Wang**: data analysis, writing – review and editing. **Jong Seung Kim**: review. **Zhiqiang Li**: writing –review and editing. **Shi Chen**: data analysis, writing – review and editing. **Meijia Gu**: writing – review and editing, visualization, supervision, resources, project administration, funding acquisition, and conceptualization.

## Ethics Statement

All animal procedures were approved by the Animal Ethics Committee of Wuhan University (No. WP20220020).

## Conflicts of Interest

The authors declare no conflicts of interest.

## Supporting information




**Supporting Information file 1**: exp270139‐sup‐0001‐SuppMat.docx

**Supporting Information file 2**: exp270139‐sup‐0002‐VideoS1.mp4

**Supporting Information file 3**: exp270139‐sup‐0003‐VideoS2.mp4

**Supporting Information file 4**: exp270139‐sup‐0004‐VideoS3.mp4

**Supporting Information file 5**: exp270139‐sup‐0005‐VideoS4.mp4

**Supporting Information file 6**: exp270139‐sup‐0006‐VideoS5.mp4

## Data Availability

All data needed to evaluate the conclusions in the paper are present in the paper and/or the Supplementary Materials.
