# Supplementary material for: Visible Photosensitizing Disinfectant Spray for Combating Multidrug‐Resistant Candidozyma Auris in Healthcare Settings
Source: Exploration (Beijing). 2026 Mar 10;6(2):20250276. doi: 10.1002/EXP.20250276 (PMC13094532; doi:10.1002/EXP.20250276)
Supplement: Supplementary file 1 — Supporting Information file 1: exp270139‐sup‐0001‐SuppMat.docx [file EXP2-6-20250276-s004.docx]

Supporting Information

**Visible Photosensitizing Disinfectant Spray for Combating Multidrug-Resistant *Candidozyma auris* in Healthcare Settings**

Xiaoyu Xu^a,#^, Ming-Yu Wu^b,c,#^, Baoping Li^a,#^, Jie Li^a,d,e,#^, Siyu Chen^a^, Luojia Chen^a^, Donghu Yu^a,e^, Liupiaopiao Yang^a^, Ziyu Hong^a^, Huaqin Pan^f,g^, Wei Xiang^d^, Shun Feng^c^, Jong Seung Kim^h^, Lianrong Wang^a,*^, Zhiqiang Li^a,e,*^, Shi Chen^a,*^, and Meijia Gu^a,e,*^

^a^Brain Center, Department of Neurosurgery, Ministry of Education Key Laboratory of Combinatorial Biosynthesis and Drug Discovery, TaiKang Center for Life and Medical Sciences, Zhongnan Hospital of Wuhan University, School of Pharmaceutical Sciences, Wuhan University, Wuhan, Hubei, 430071, China.

^b^College of Biomedical Engineering, Sichuan University, Chengdu 610065, China

^c^Sichuan Engineering Research Center for Biomimetic Synthesis of Natural Drugs, School of Life Science and Engineering, Southwest Jiaotong University, Chengdu, Sichuan, 610031, China.

^d^Department of Medical Intensive Care Unit, Maternal and Child Health Hospital of Hubei Province, Tongji Medical College, Huazhong University of Science and Technology, Wuhan, 430070, Hubei, China.

^e^Hubei International Science and Technology Cooperation Base for Research and Clinical Techniques for Brain Glioma Diagnosis and Treatment, Wuhan University, Wuhan, Hubei, 430071, China.

^f^Zhongnan Hospital of Wuhan University, Institute of Hepatobiliary Diseases of Wuhan University, Transplantation Intensive Care Unit, Transplant Center of Wuhan University, Hubei Key Laboratory of Medical Technology on Transplantation, Wuhan, 430071, China.

^g^Department of Critical Care Medicine, Zhongnan Hospital of Wuhan University, Clinical Research Center of Hubei Critical Care Medicine, Wuhan 430071, China.

^h^Department of Chemistry, Korea University, Seoul 02841, Korea

^#^ X. X, M-Y. W, B. L and J. L contributed equally to this work.

*To whom correspondence may be addressed. Email: mjgu@whu.edu.cn, shichen@whu.edu.cn, lizhiqiang@whu.edu.cn, lianrong@whu.edu.cn

**Materials and Methods**

**Materials and Instruments**

All chemical reagents were obtained from J&K Scientific and used without further purification. Dulbecco's Modified Eagle Medium (DMEM, 11965092), Phosphate Buffered Saline (PBS, 10010031), Penicillin-Streptomycin (P/S, 15070063), heat-inactivated Fetal Bovine Serum (FBS, 10100147), 0.25% trypsin-EDTA (252000056) were purchased from Gibco, Thermo Fisher Scientific, Waltham, USA. Nutrient Broth (NB, HB0108) and yeast extract peptone dextrose medium (YPD, HB5193) were purchased from Qingdao Hope Bio-Technology Co., Ltd., Qingdao, China. The Live & Dead^TM^ Viability/Cytotoxicity Assay Kit (NucGreen/EthD-III, L6060L), Live & Dead^TM^ Animal Cells Viability/Cytotoxicity and Assay Kit (Calcein AM/PI, L6037L) were purchased from UElandy, Suzhou, China. Mannitol (M813424), The H&E staining kit (S191003) was from Pinuofei, Wuhan, China. Milli-Q water was supplied by the Milli-Q Plus System (Millipore, MA, USA). An OPPLE LED lamp was employed as the source of white light. The luminescence power was adjusted by tuning the height of the white light source and measured by a Compact Power and Energy Meter Console (PM100D, Thorlabs) together with a Microscope Slide Power Meter Sensor Head (S170C, Thorlabs). Multiska GO microplate spectrophotometer (Thermo Scientific, MA, USA) was employed to measure the photoluminescence spectra in bulk solutions, and a SpectraMax i3x Multi-Mode Microplate Detection System (Molecular Devices, CA, USA) was used to measure the photoluminescence spectra in 96-well plates. The Field Emission Scanning Electron Microscope (FESEM, Sigma 300, Zeiss, Germany) and the Transmission Electron Microscope (TEM, HT7800, HITACHI, Japan) were used to collect the morphology images. Fluorescence images were collected with a Confocal Laser Scanning Microscope (CLSM, Eclipse Ti-S, Nikon, Japan) and analyzed by using NIS-Elements AR software. The zeta potential and particle size were measured using a Zetasizer Nano ZS instrument (Malvern, UK). NMR spectra were recorded using a Bruker AMX-400 or Bruker Avance Neo-600. Chemical shifts were given in *δ* relative to the internal reference with DMSO-*d*_6_ as the internal standard. Antibodies used in this study are presented in Table S4, Supporting Information.

**Synthesis and Characterization of TB**

**Scheme S1.** Synthetic route to TB.

50 mg of TBTCP (0.093 mmol) was dissolved in 30 mL of acetonitrile. Then, 100 mg of 4-(bromomethyl)phenylboronic acid (0.465 mmol) was added. The reaction mixture was heated to 78 ℃ under N_2_ protection for 24 h. After cooling to room temperature, the mixture was concentrated under reduced pressure to remove the solvent. The crude product was purified using silica gel column chromatography with DCM/MeOH/trifluoroacetic acid (*V:V:V* = 15:1:0.05) as the eluent. This resulted in the isolation of 53.4 mg of a dark red solid, yielding 76.3%. ^1^H NMR (600 MHz, DMSO-*d*_6_) *δ*. 9.10 (d, *J* = 6.7 Hz, 2H), 8.41 (d, *J* = 6.7 Hz, 2H), 8.31 (d, *J* = 4.1 Hz, 1H), 8.08 (s, 1H), 7.84 (d, *J* = 7.9 Hz, 2H), 7.59 (d, *J* = 8.6 Hz, 2H), 7.53-7.39 (m, 5H), 7.34 (d, *J* = 7.6 Hz, 4H), 7.16-7.01 (m, 8H), 6.97 (d, *J* = 8.4 Hz, 2H), 5.79 (s, 2H). ^13^C NMR (150 MHz, DMSO-*d*_6_) *δ.* 146.55, 145.78, 144.89, 143.61, 139.51, 136.03, 135.86, 135.72, 134.83, 132.59, 130.68, 129.73, 127.45, 126.63, 124.74, 123.88, 123.13, 122.21, 116.27, 104.62, 62.38. HRMS (ESI): m/z [M-Br^-^]^+^ calcd for C_41_H_31_BN_3_O_2_S_2_: 672.6480; found 672.1951.

**ROS Generation Efficiency Measurement (SOSG as detector)**

For the monitoring of ^1^O_2_, 9,10-anthracenediyl-bis(methylene)dimalonic acid (ABDA) was employed, with rose bengal red (RB) serving as the control. In water, 50 μM of SOSG was mixed with either 5 μM of TB, and then irradiated under white light for 0 to 1170 s. The ROS generation efficiency was calculated by recording the fluorescence of SOSG at 525 nm for various exposure times to light.

***In situ* EPR Spectrum of TB**

The spin-trapping agents 2,2,6,6-tetramethylpiperidine (TEMP) and 5,5-dimethyl-1- pyrroline-*N*-oxide (DMPO) were explored to detect ^1^O_2_ or radicals in PBS by EPR, respectively. In the experiment, the spin-trapping agents were mixed with **TB** at 5 μM and irradiated with an 808 nm laser at 1.0 W cm^−2^ for 4 min, then the EPR spectra were recorded within 10 min.

**Inhibition of Biofilm Formation**

Confocal dishes and a 96-well microtiter plate were pre-treated with FBS overnight. The next day, *C. auris* cells were resuspended at OD_600_ = 0.1 with Spider medium. Next, TB was added to a final concentration ranging from 0 μM to 5 μM in confocal dishes and a 96-well microtiter plate, incubated for 15 min at 37°C, and finally irradiated with/without white light (80 mW cm^-2^) for 20 min. After incubation for 24 h, the planktonic fungi were gently decanted with PBS 3 times. Photographs of Biofilms in the dishes were then taken using a digital device. The biofilms in the 96-well microtiter plate were first fixed with methanol for 15 minutes. Then, they were stained with 0.1% crystal violet (CV71012314, Sinopharm) for 5 minutes. The excess dye was washed away with PBS, and the plate was left to dry. Subsequently, the biofilms were decolorized with 30% acetic acid at 37°C for 15 minutes to ensure uniform dissolution. Finally, the absorbance was measured using the Molecular Devices SpectraMax i3x Multi-Mode Microplate Detection System.

**Evaluation of Biofilm Microenvironment**

Mature biofilms were formed as previously described. The biofilm was incubated with 5 μM TB and PBS for 15 min at 37°C and then irradiated with white light (80 mW cm^-2^) for 20 min or in the dark. The pH values of mature biofilms were recorded using a pH meter. In addition, the supernatants were collected, and the concentration of H_2_O_2_ and GSH of biofilms was evaluated using a micro H_2_O_2_ assay kit and a GSH assay kit (Solarbio).

**Detection of Water-Insoluble Polysaccharides**

Pretreatment of biofilms was processed as previously described. Then, the biofilms were washed by ddH_2_O 3 times to remove the water-soluble polysaccharides and water-insoluble polysaccharides were dissolved in 0.5 M NaOH. Polysaccharide quantitation experiments were performed following an anthrone-sulfuric acid method. Briefly, 3 mL of anthrone-concentrated sulfuric acid (2 mg mL^-1^) was added to 1 mL of glucose solution of differing concentrations (0, 5, 10, 20, 30, 40, or 50 μg mL^-1^), and samples. After 10 min in a boiling water bath, the absorbance at OD_620_ of the samples was measured on a microplate reader.

**Preparation of TB Functional Materials**

The solvent evaporation method was used to prepare TB-coated materials. Various materials functionalized with TB were obtained by adding 400 uL of 5 μM TB (dissolved in anhydrous ethanol) to 1 cm diameter discs of different materials (surgical gowns, surgical instruments, operating tables, and polyvinyl chloride (PVC)), which were naturally air-dried at room temperature and then subjected to freeze-drying. The final concentration of TB was 2.5 μmol cm^-2^.

**TB Functional Materials Characterization**

FESEM was employed to characterize the scanning transmission electron microscopy-energy dispersive X-ray spectroscopy (STEM-EDX) elemental distribution of TB functional materials.

**Water Contact Angle (WCA) Measurement of the Diversity of Materials**

The hydrophobicity of all materials before and after TB-coated were measured using Drop Shape Analyzer (DSA25S, KRUSS, Germany) in terms of WCA by sessile drop method at room temperature in air. Materials were placed on the sample holder and the liquid drop (double distilled water) with a volume of approximately 6 µL was carefully dropped on the surface of the materials followed by immediate capture of images of the drop. WCA was calculated by measuring the angle between the water drop and different materials using software ADVANCED 1.13.

**Anti-Fungal Effect of TB Functional Materials**

*C. auris* was incubated overnight at 28°C in YPD media, harvested and diluted with PBS to an OD_600_ value of 0.1 before use. 400 µL of *C. auris* dilution was added to the TB-coated and uncoated materials. The TB-coated material was irradiated with white light at 80 mW cm^-2^ for 0 min, 10 min and 20 min, and the uncoated material was exposed to UV irradiation at 200 µmW cm^-2^ for 10 min and 20 min. The unirradiated material served as a control.

For plate counting experiments, materials with the fungal solution were placed into centrifuge tubes with 2 mL of PBS and vortexed at the highest speed for 2 min to separate as many fungal cells as possible adhering to the material. Then, the dilutions were used for plate counting assay and SEM imaging as previously described.

**Assessment Biofilm Eradication Ability of TB on High-Touch Surfaces**

Mature biofilms were formed as previously described. on surgical gowns, surgical instruments, operating tables, and PVC. In the TB group, 20 μL PBS solution containing a high concentration of TB (5 μM) was added and irradiated (80 mW/cm^-2^) for 20 min. The UV group was irradiated with UV for 20 min. The control group was treated with PBS. The adhesion and formation of biofilm were imaged by FESEM.

**RNA Isolation and Quantitative Real-Time PCR (****qRT-PCR)**

To further investigate the effects of TB with/without white light irradiation on biofilm formation, the total RNA of *C. auris* treated with different concentrations of TB (0 and 5 μM) was immediately pretreated with FastPrep®-24 5G (MP Biomedicals) and then extracted with TRIzol reagent (15596-026, Life Technologies). RNA was reverse transcribed using a Hifair II 1st Strand cDNA Synthesis Kit (11119ES60, Yeasen). First, 1 μg of RNA, 1 μL of Oligo (dT)_18_ and 13 μL of H_2_O were denatured at 65°C for 5 min. After cooling immediately on ice, RNA was reverse transcribed using the following reaction mixture composed of 4 μL of 5× reaction buffer and 2 μL Hifair Enzyme Mix. According to the manufacturer, Amplification conditions were 5 min at 25°C, followed by 42°C for 30 min and deactivation at 85°C for 5 min. Each 20 μL qRT-PCR mixture contained 10 μL of Taq Pro Universal SYBR qPCR Master Mix (Q712-02, Vazyme), 5 μL of cDNA, 0.5 μL of each primer (25 μM) and 4 μL of RNase-free water. The amplification reaction was performed according to the manufacturer’s instructions as follows: 3 min of activation at 95°C, followed by 40 cycles at 95°C for 5 s, and a final extension cycle at 60°C for 30 s. A CFX96^TM^ Real-Time PCR System (Bio-Rad, USA) was used for qRT-PCR, according to the manufacturer’s protocol. *C. auris ACT1* was used to normalize gene expression. The 2^−ΔΔCt^ method was used to determine the relative mRNA expression levels of target genes from 3 independent replicates of each sample performed in triplicate.

**Antimicrobial Photodynamic Inactivation Resistance Testing**

To investigate the development of resistance of TB to antimicrobial photodynamic inactivation (PDI) treatment, 10 cycles of PDI were carried out. The *C. auris* was incubated overnight at 28 °C in YPD media, harvested, and diluted with PBS containing 1 µM TB to an OD_600_ value of 0.1 before use. Each cycle had a total irradiation time of 20 minutes. After plating the surviving fungi from the previous PDI cycle on YPD agar and incubating for 18 h at 28 °C, single colonies were isolated, and a new set of fungal cultures was prepared and illuminated. PDI treatments were repeated under similar conditions. After treatment of every cycle, samples were used for fluorescent staining. Three independent experiments were carried out. After ten cycles of PDI, the cells were repeatedly incubated overnight and diluted with PBS containing 5 μM TB to an OD_600_ value of 0.1. Then, the samples were irradiated with white light for 20 min (80 mW cm^−2^) for fluorescent staining and qRT‐PCR. For fluorescent staining, cultures were stained with the Live & Dead Viability/Cytotoxicity Assay Kit (UElandy) and imaged with CLSM. A 488 nm laser and a 515–550 nm emission filter were used for the green channel, while a 561 nm laser and a 570–620 nm emission filter were used for the red channel. To examine the effect of PDI treatment on the expression of *ERG11*, untreated samples after ten cycles of PDI were incubated overnight at 28 °C. The isolation of RNA and the process of qRT‐PCR were conducted as mentioned above (section RNA Isolation and qRT‐PCR).

**Measurement of** **Malondialdehyde (MDA)**

Mature biofilms were formed as previously described. The biofilm was incubated with 5 μM TB and PBS for 15 min at 37°C and then irradiated with white light (80 mW cm^-2^) for 20 min or in the dark. The production of MDA (a toxic byproduct of Lipid Peroxidation, LPO) was evaluated with MDA Content Assay Kit (Solarbio) according to the manufacturer’s protocols.

**Experimental Model of Simulating Ventilator Infections**

Preparation of PVC tubes: the PVC is pre-incubated with biofilm as shown above.

Twelve-week-old SD rats (200–250 g) were used in intubation experiments to simulate ventilator infections, and the rats were randomly divided into 3 groups of 3 rats each. Before the simulated ventilator pneumonia infection, rats (except controls) were injected intraperitoneally with 25 mg dexamethasone per rat twice a week for a month to develop an immunosuppressed state. After intraperitoneal injection of sodium pentobarbital (2%, 0.2 mL100 g^-1^), the skin was prepared routinely from the mandible to just above the chest, and a small incision of about 1.5 cm in length was cut from the submandibular region to the chest. The superficial fascia was cut to expose and bluntly separate the muscles until the trachea was exposed. Briefly, to minimize tracheal trauma, the needle is inserted into the trachea, a PVC catheter is inserted inside the needle, and the needle is subsequently withdrawn, leaving one end of the catheter inside the main trachea. The other air-exposed side of the catheter was the biofilm attachment side. Subsequently, the catheter was fixed, and the subcutaneous tissue and skin were sutured and sterilized with iodophor and routinely reared.

During the intubation period, the PBS group was treated with 20 µL of PBS per day, and the TB group was treated with 20 µL of TB (5 µM) per day and exposed to light for 20 min (80 mW cm^-2^). In addition, the group without intubation was the control group. The rats were executed on Day 7 of intubation, and the lungs were taken for HE staining and tissue immunofluorescence. A semiquantitative scoring system for the lung injury score was adopted according to the previous publication.

***In Vitro* Hemolysis Evaluation**

An *in vitro* hemolysis test was employed to assess the hemocompatibility of TB. Fresh rat erythrocytes were resuspended in PBS (20% v/v). 100 μL of erythrocyte suspension and 100 μL of PBS solution with different concentrations of TB (0, 1, 2, 5, 10, 25 μM) were incubated at 37°C for 30 min, and then centrifuged at 2000 rpm for 10 min. The OD of the supernatant was measured by a microplate reader at 540 nm. Water represented the positive control and PBS represented negative control. Hemolytic quantification was done as follows: Hemolysis (%) = Ap/At×100%, wherein Ap is the supernatant OD of the dosing group and At is the supernatant OD of the positive control.

***In Vitro* Cell Viability Evaluation**

Cell viability was determined by the standard WST-8 (2-(2-methoxy-4-nitrophenyl)-3-(4-nitrophenyl)-5-(2,4-disulfophenyl)-2H-tetrazolium, monosodium salt) Cell Counting Kit-8 assay (CCK-8, C0040, Beyotime). COS-7 cells were seeded at a density of 5 × 10^3^ cells per well in 96-well microplates with 100 μL of culture medium and cultured overnight to reach 70–80% confluence. Then, the medium was replaced with 100 μL of fresh medium containing different concentrations of TB (0, 1, 2, 5, 10, and 25 μM) and incubated for 30 min at 37°C. 0.1% DMSO was used as a vehicle control. After irradiation under white light for 20 min (80 mW cm^-2^), 10 μL of 12 mM CCK-8 stock solution mixed with 90 μL of PBS was added to each well for an additional 2.5 h of incubation. The absorbance was measured at 450 nm using a SpectraMax M2 microplate reader (Molecular Devices). Cell viability (%) was calculated as follows: (OD_450_ test/OD_450_ control) × 100%. The detection method for HaCaT cells was consistent with the above.

***In Vitro* Cell Live/Dead Staining**

CLSM was adopted to observe the viability of COS-7 cells. COS-7 cells were treated in the same manner as those used for sample preparation for CCK-8 assay and stained with the Live & Dead^TM^ Animal Cell Viability/Cytotoxicity Assay Kit (Calcein AM, PI) following the instructions. Dead cells were stained red, while live cells were stained green. Cells were imaged by CLSM, with a 488 nm laser and a 515–550 nm emission filter for the green channel, a 561 nm laser, and a 570–620 nm emission filter for the red channel. The detection method for HaCaT cells was consistent with the above.

***In Vivo* Toxicity Study**

The toxicity of TB *in vivo* was evaluated in healthy rats. The 6 rats were randomly divided into 2 groups (3 in each group, named the TB group and the control group). To evaluate the toxicity of TB on normal skin, 10 μL PBS solution containing a high concentration of TB (30 μM) was applied to the skin of rats in the TB group every day for the first five days of treatment. Rats treated with TB spray with daylight exposure were also observed. The skin appearance of each rat was recorded by a camera without treatment in the control group. On the seventh day, jugular vein blood was taken from each rat, a whole blood routine blood test was performed, and serum was separated for physiological and biochemical tests. One rat in the TB group and the control group was randomly selected to separate the heart, lung, liver, spleen, kidney, and skin for pathological evaluation.

***In Vivo* Long-term toxicity test**

Female SD rats (200–250 g) rats (12 weeks, n = 3) were injected intravenously (i.v.) with 200 μL of TB and PBS (TB = 30 μM). Body weight was constantly monitored for 30 days, and then rats were sacrificed. Blood samples were obtained for hematologic and clinical chemistry analysis. Each group's major organs (heart, liver, spleen, lung and kidney) were isolated and placed in 4% paraformaldehyde for 24 h, then sectioned and H&E stained for histological examination. Blood samples were also harvested from the above rats to measure the levels of albumin (ALB), aspartate aminotransferase (AST), alanine aminotransferase (ALT), blood urea nitrogen (BUN) and urea nitrogen (UREA).

**Animal Experiment Ethics**

All animal experiments were approved by the Animal Ethics Committee of Wuhan University (No. WP20220020). All surgical procedures followed the standard guidelines.

**Supplemental Figures**


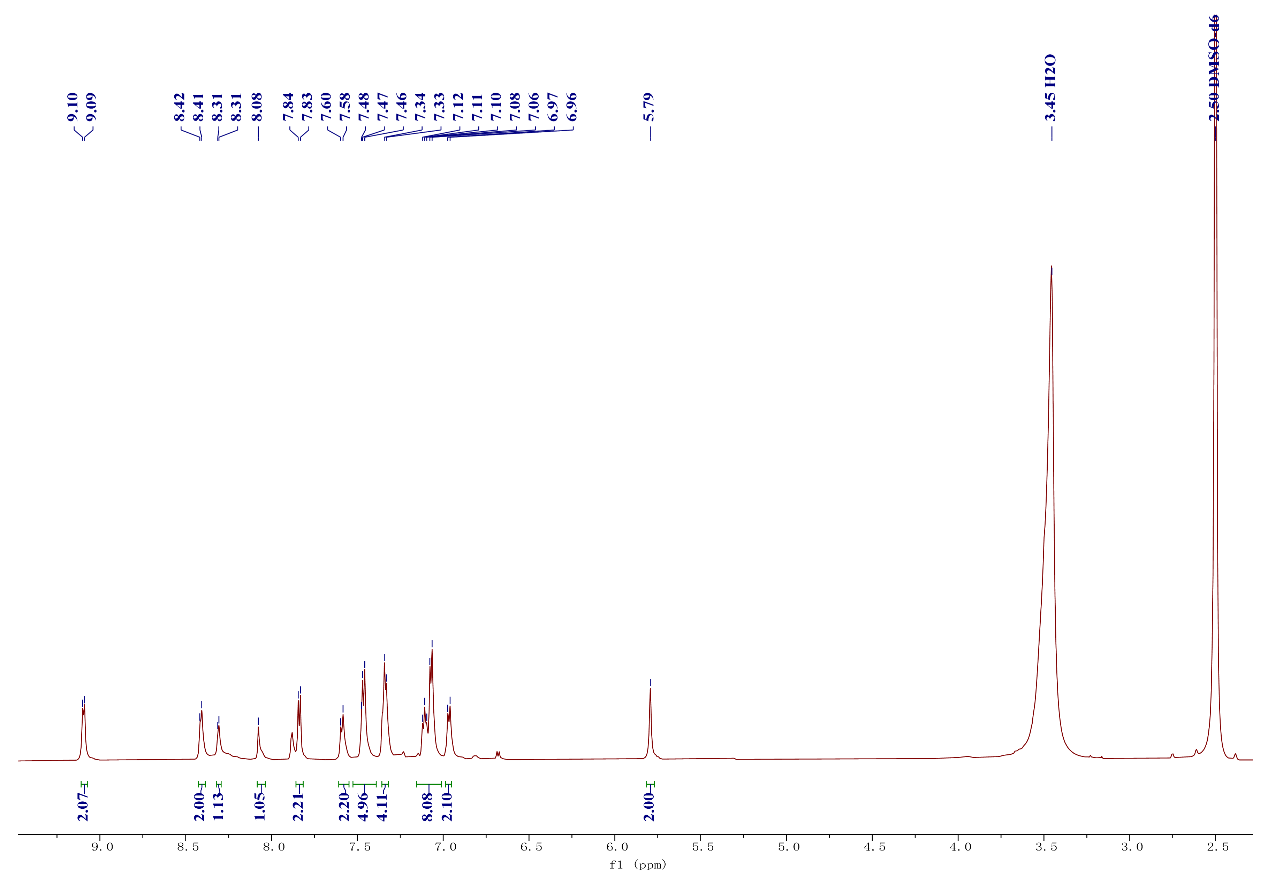


**Figure S1.** ^1^H NMR of TB in DMSO-*d*_6_.


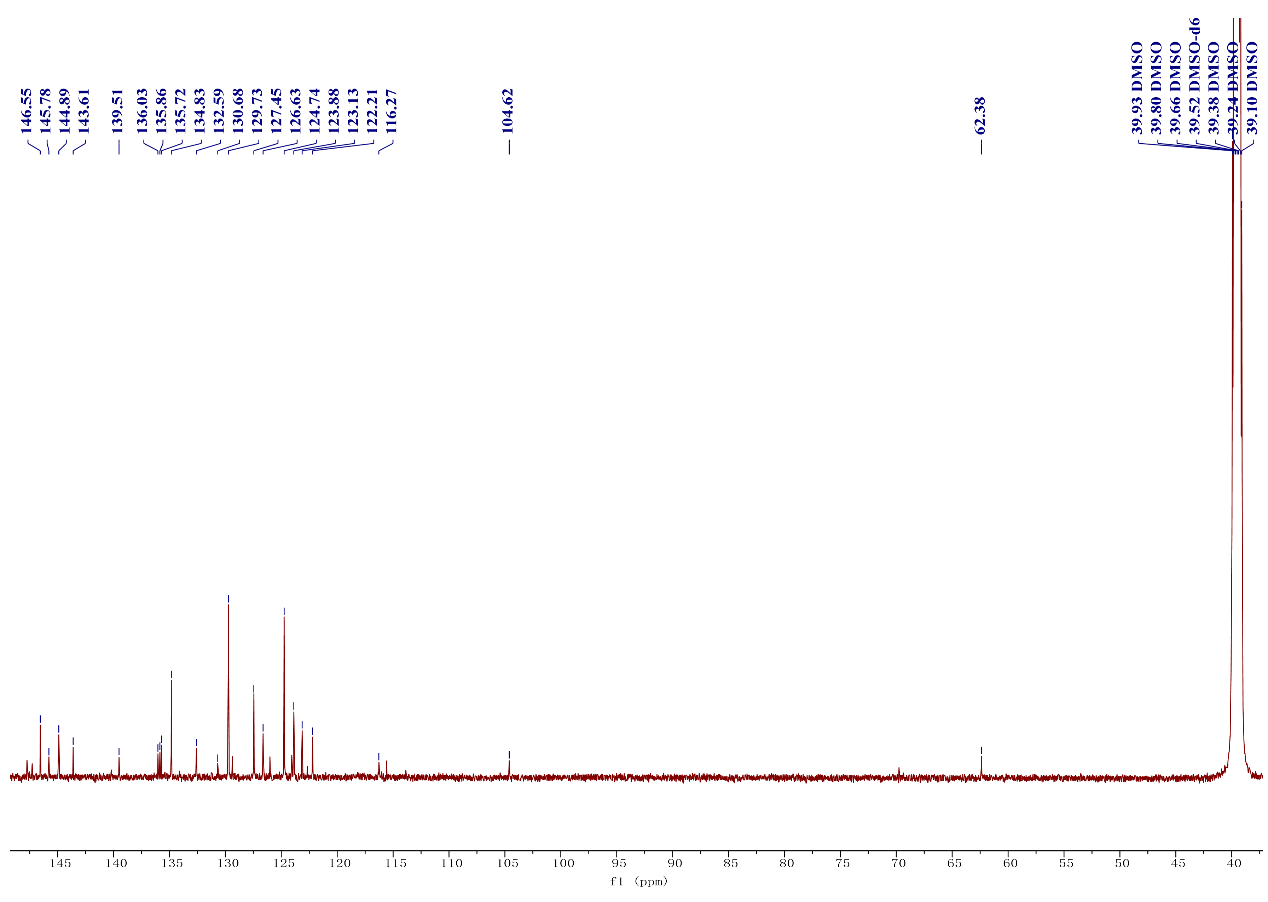


**Figure S2.** ^13^C NMR of TB in DMSO-*d*_6_.


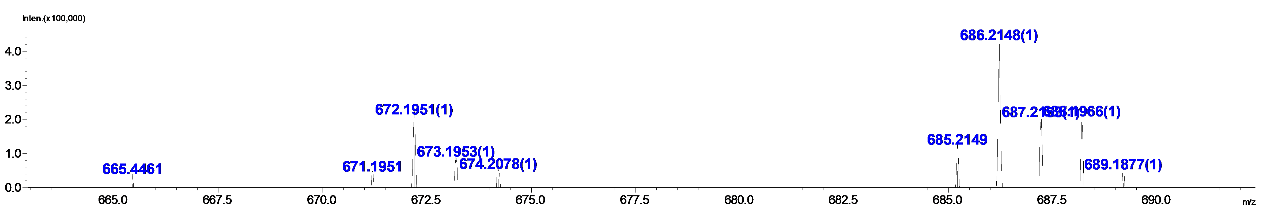


**Figure S3.** HRMS of TB.


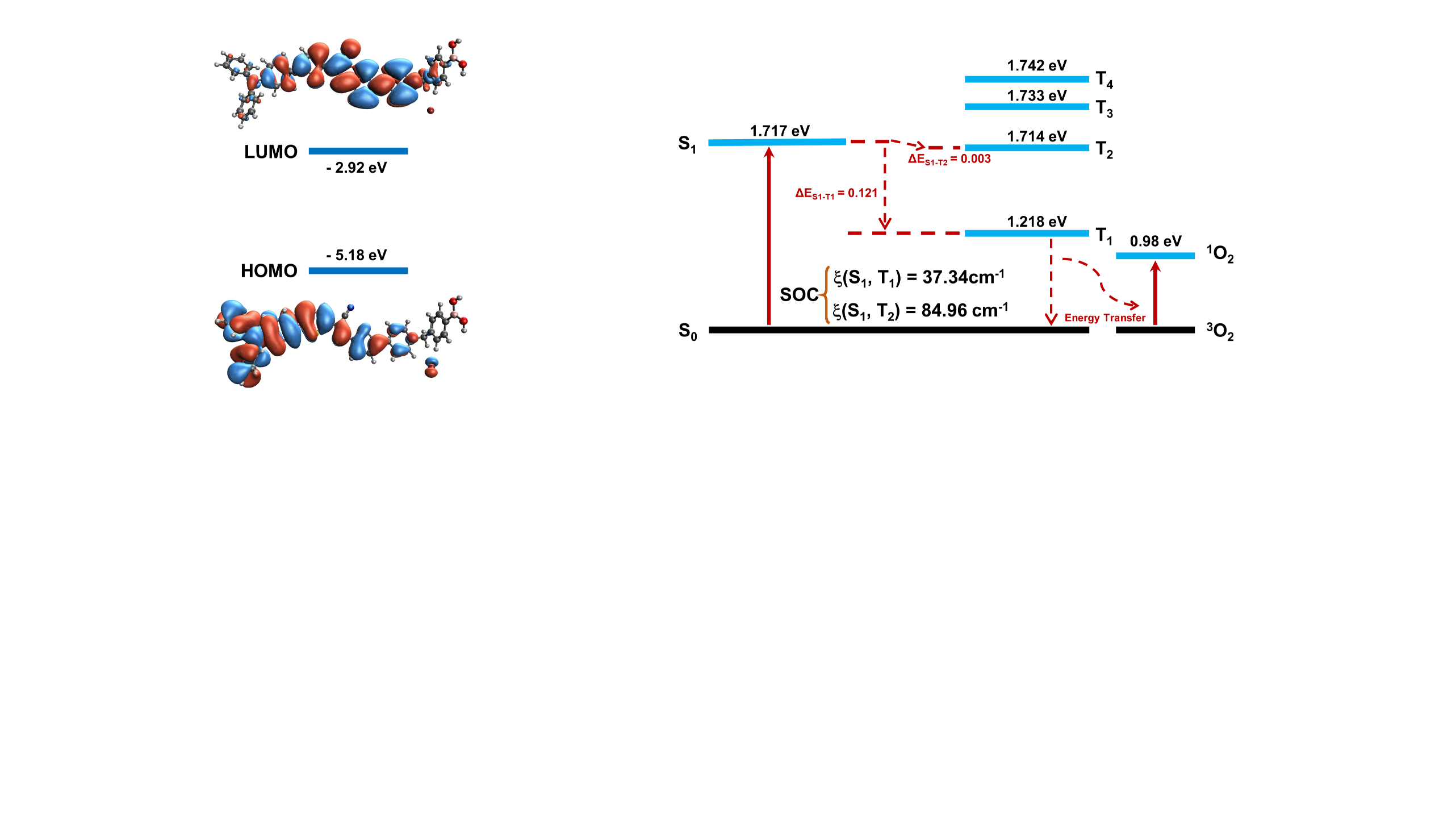


**Figure S4.** HOMO and LUMO distribution, Δ*E*_S-T_ values of TB calculated by functional PBE0 and basis set 6-31G (d, p).


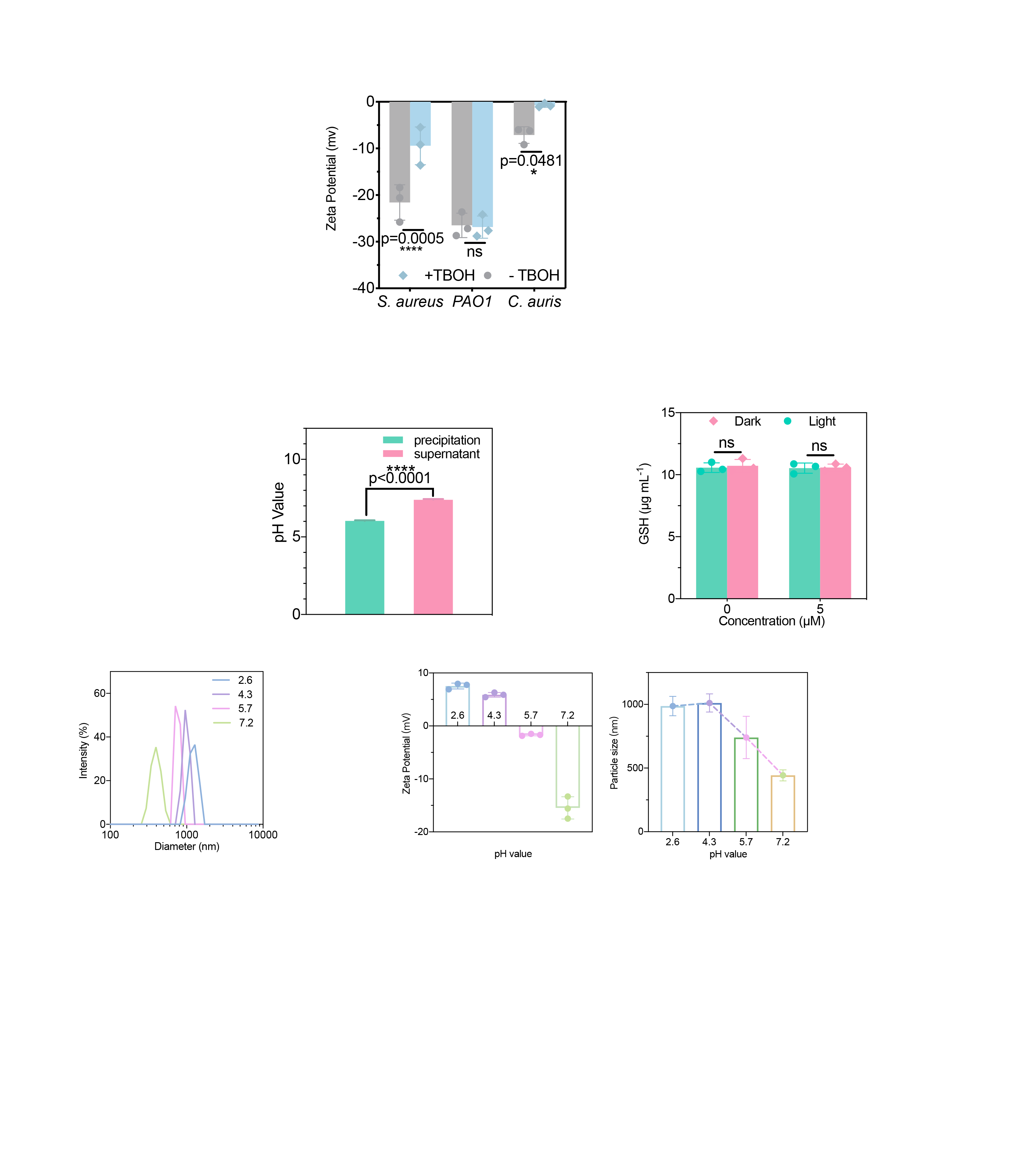


**Figure S5.** Zeta potential value and hydrodynamic size distributions of TB under different pH values (pH=2.6 4.3, 5.7 and 7.2) in PBS.


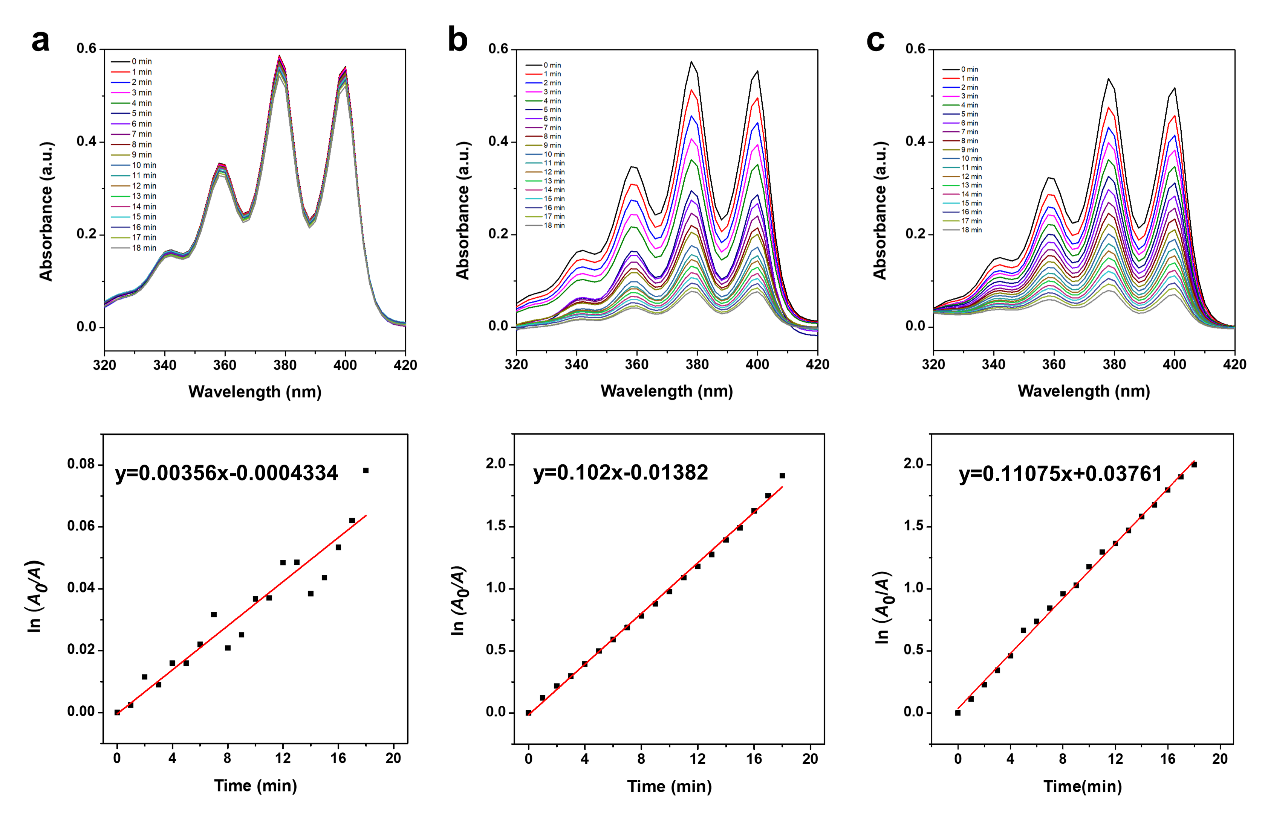


**Figure S6.** Absorption spectra of ABDA (50 μM) in water (a) or in the presence of 5 μM TB (b), and Rose Bengal (c) under white light irradiation.


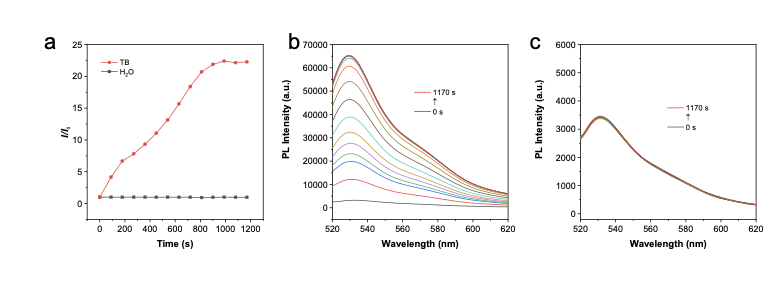


**Figure S7.** The relative changes in fluorescence intensity and photoluminescence spectra of SOSG at 515 nm in each sample under white light irradiation from 0 to 1170 seconds.


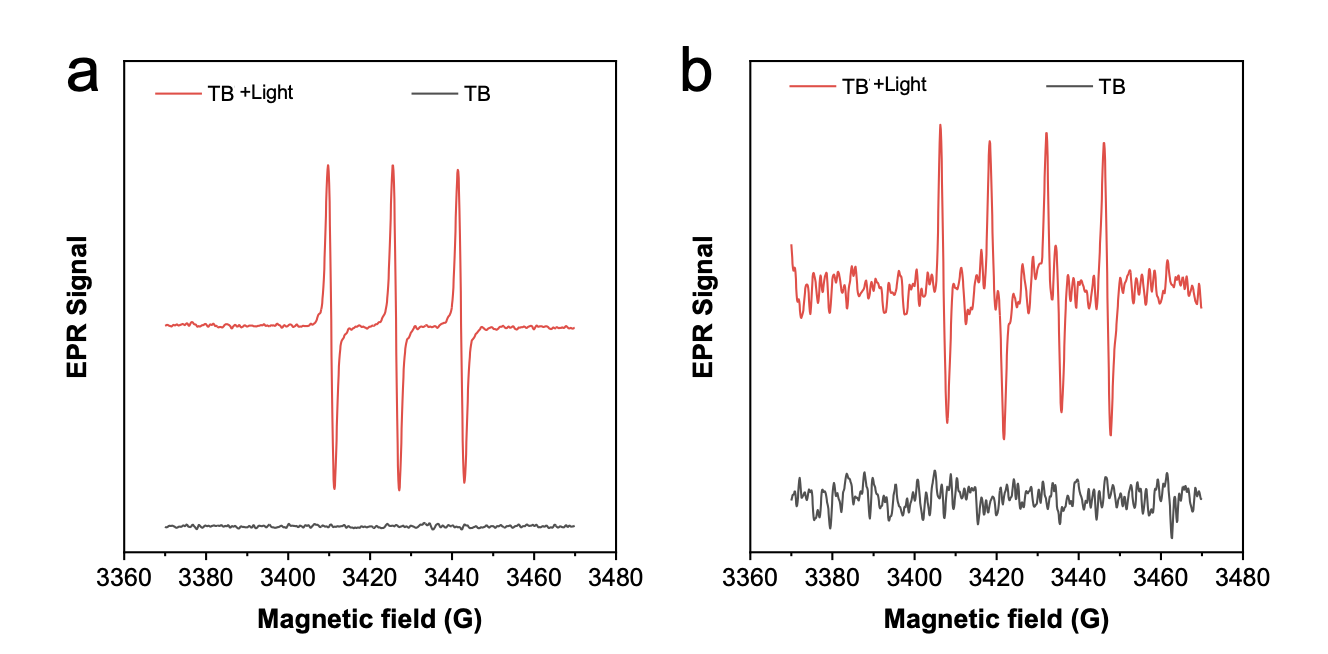


**Figure S8.** EPR signals of TB+light with TEMP (a) and DEMP (b).


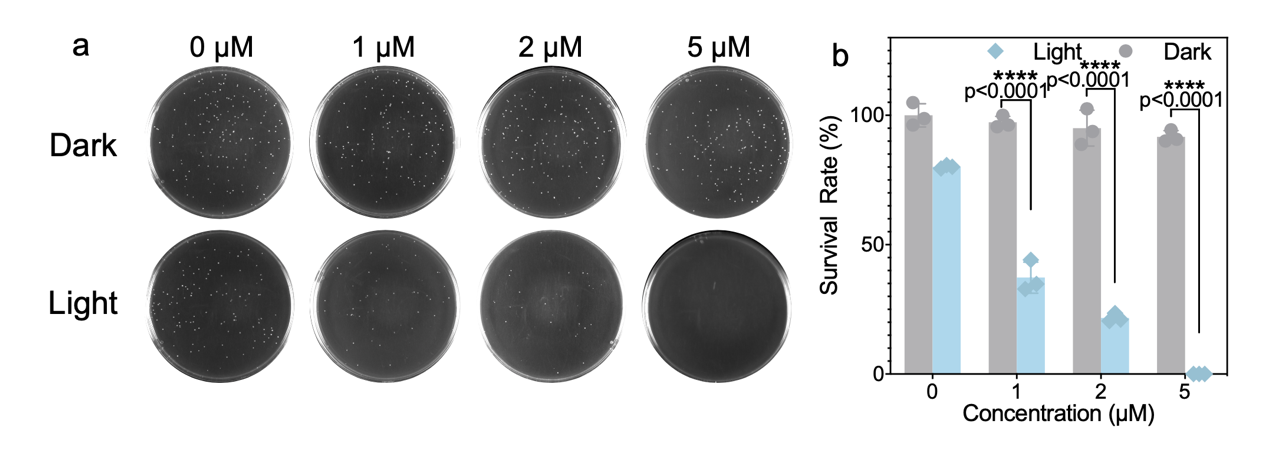


**Figure S9.** Photodynamic antifungal effect of TB. a) Representative images of YPD agar plates employed to quantify *C. auris* viability. b) *C. auris* survival rate evaluated by the serial dilution test on YPD agar.


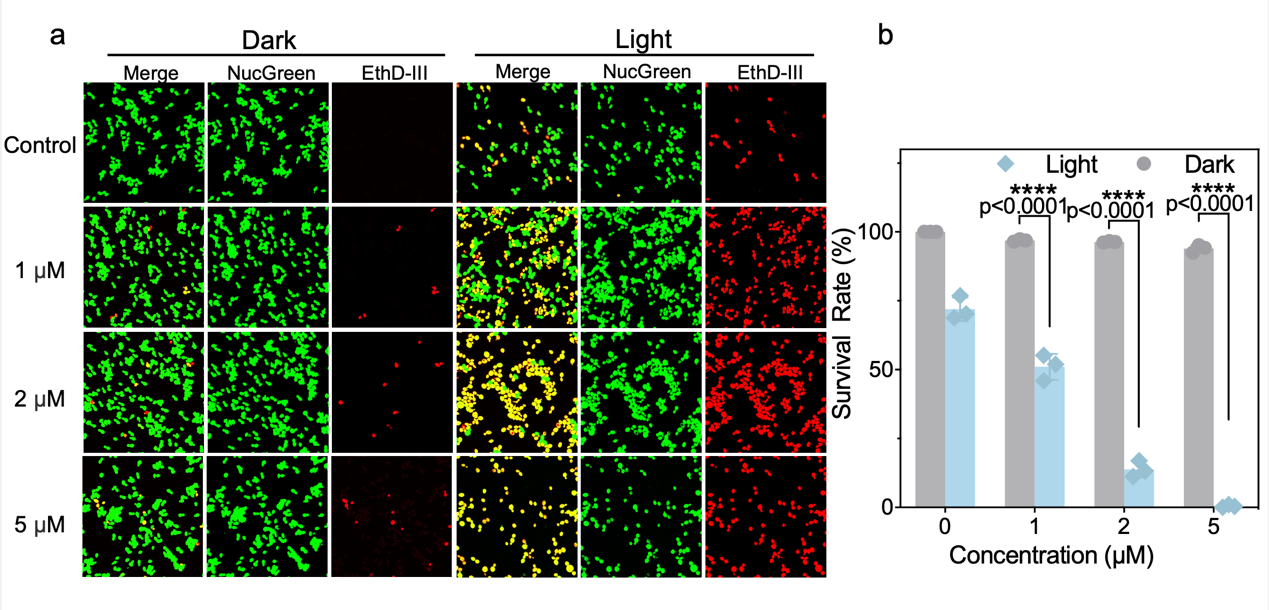


**Figure S10.** Photodynamic antifungal effect of TB. a) CLSM images of *C. auris* after applying a Live & Dead^TM^ Viability/Cytotoxicity Assay Kit and treatment with various concentrations of TB under white light irradiation (80 mW cm^-2^) or in the dark for 20 min. A 488 nm laser with a 515–550 nm emission filter (green channel) and a 561 nm laser with a 620–720 nm emission filter (red channel) were used for imaging. b) *C. auris* survival rate evaluated by the Live/Dead staining assay.

**
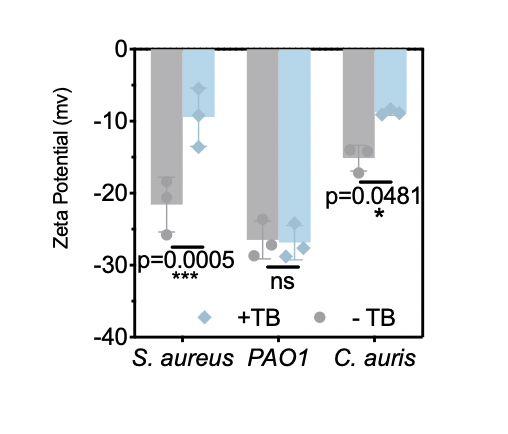
**


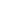


**Figure S11.** Zeta potential results of *S. aureus,* PAO1 and *C. auris* in ddH_2_O solution pre-treated with or without 5 μM TB.


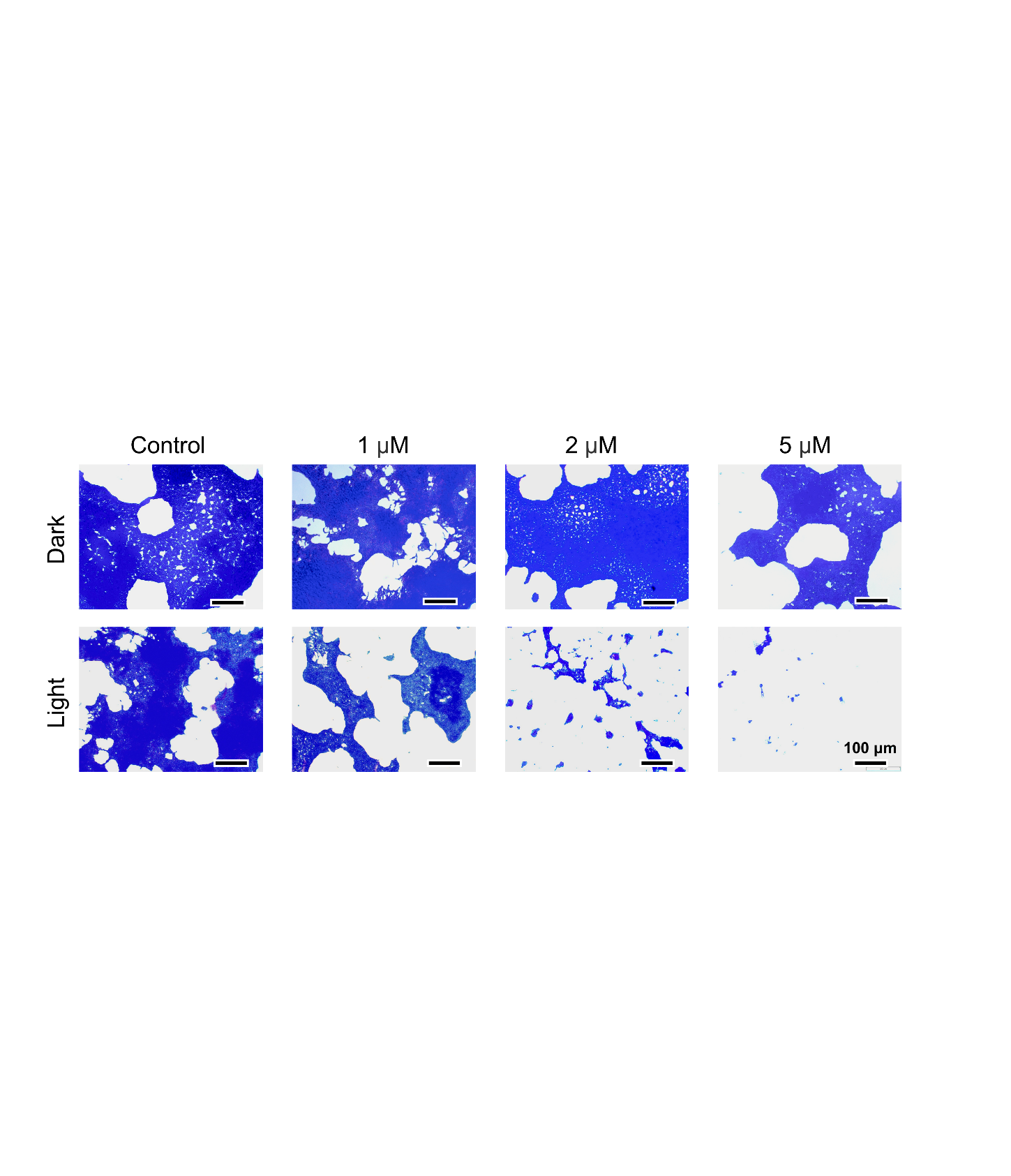


**Figure S12.** Representative crystal violet staining images of *C. auris* after treatment with TB in the dark or under white light irradiation (80 mW cm^-2^) for 20 min (scale bar: 100 μm).


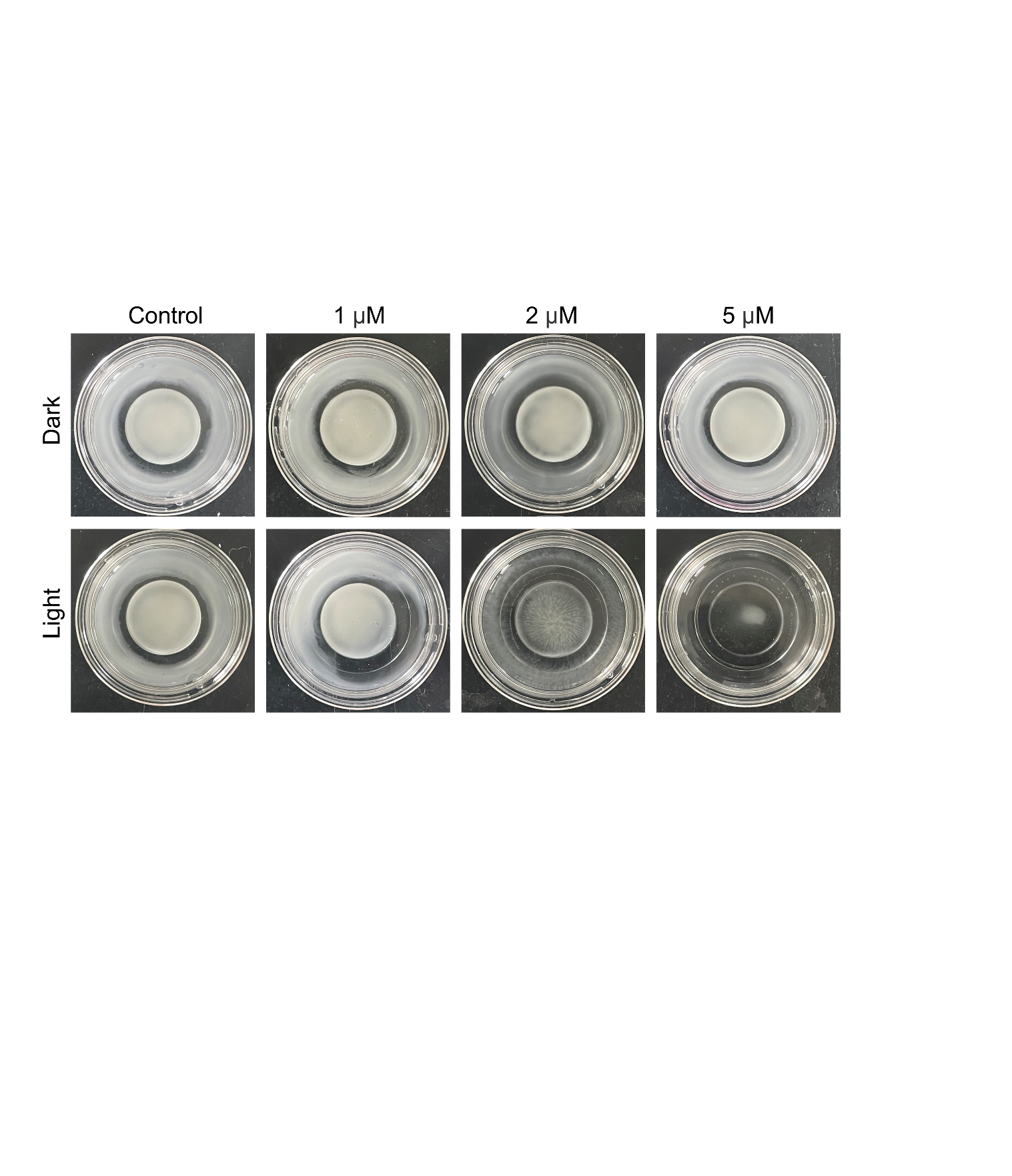


**Figure S13.** Representative confocal dish images of *C. auris* after treatment with TB in the dark or under white light irradiation (80 mW cm^-2^) for 20 min.


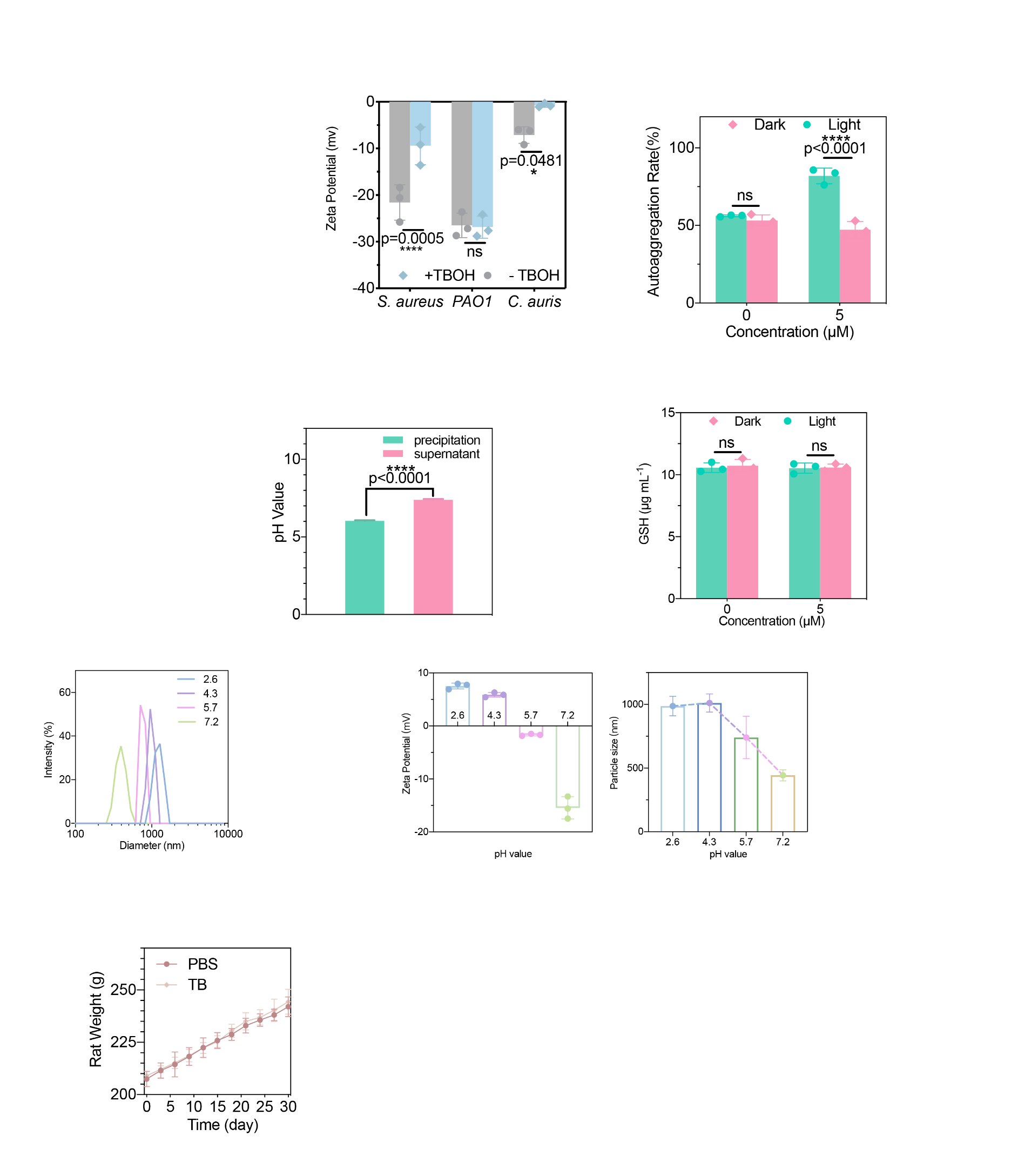


**Figure S14.** The autoaggregation rate of *C. auris* after different treatments.


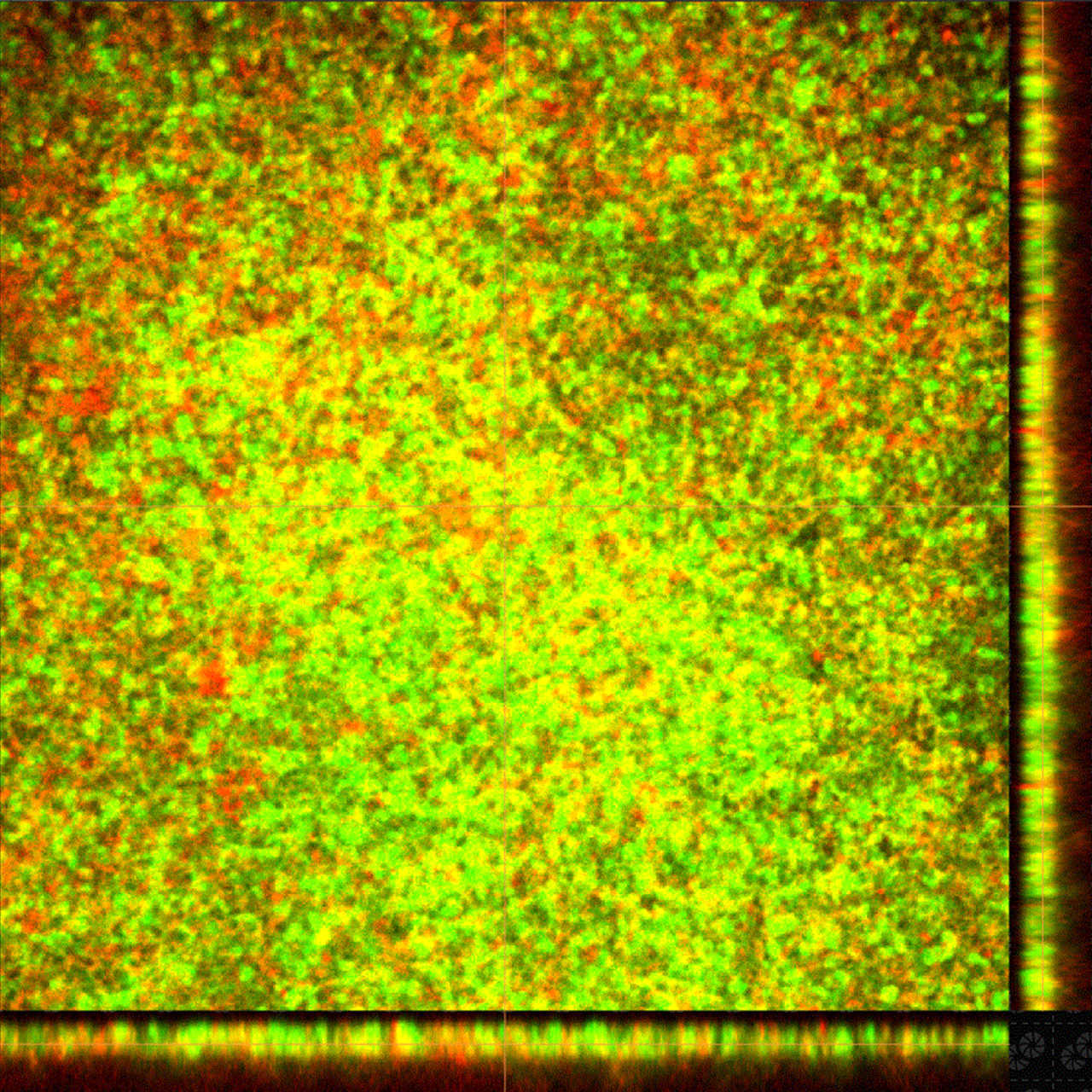


**Figure S15.** *C. auris* biofilm images treated with NucGreen and TB. The green fluorescence represents *C.auris* in the biofilm, and the red fluorescence represents polysaccharides in the biofilm targeted by TB. A 488 nm laser and a 515–550 nm emission filter were employed for the green channel, and a 561 nm laser and a 570–620 nm emission filter were used for the red channel.


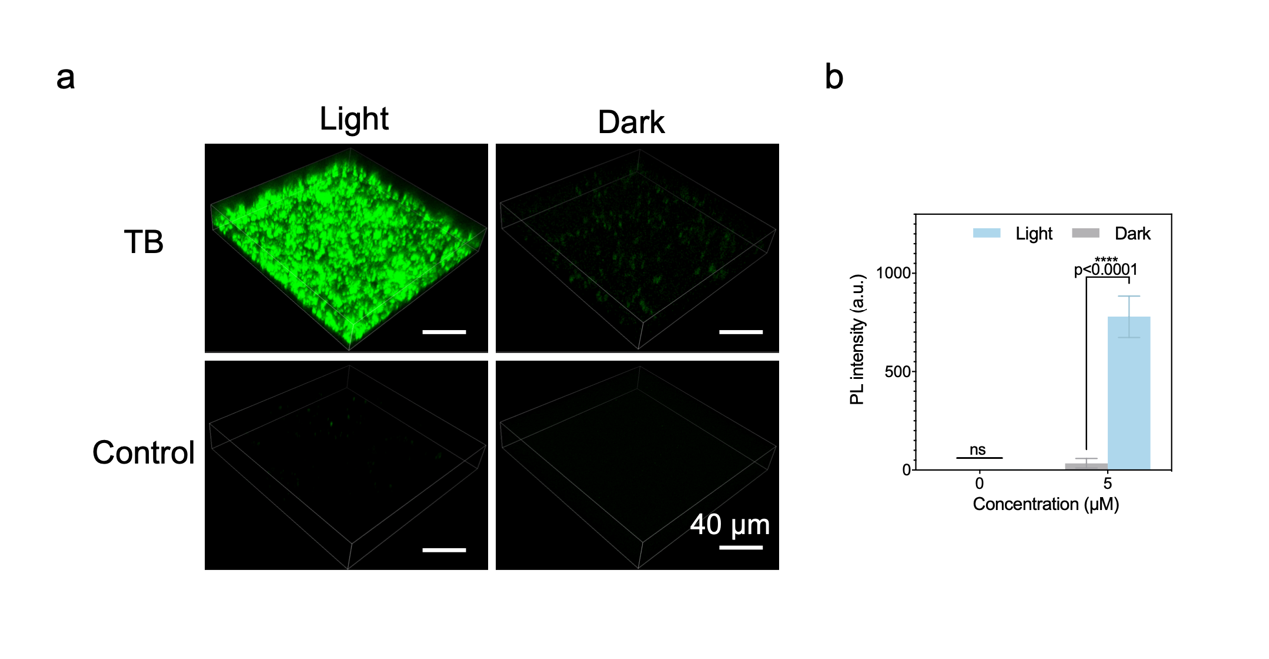


**Figure S16.** Evaluation of extracellular reactive oxygen species (ROS) production in biofilms. CLSM 3D images of biofilms (a) and quantification of the corresponding fluorescence intensity (b) according to COMSTAT 2.0 for five random observation points of *C. auris* biofilms. The green channel uses a 488 nm laser and a 515–550 nm emission filter.


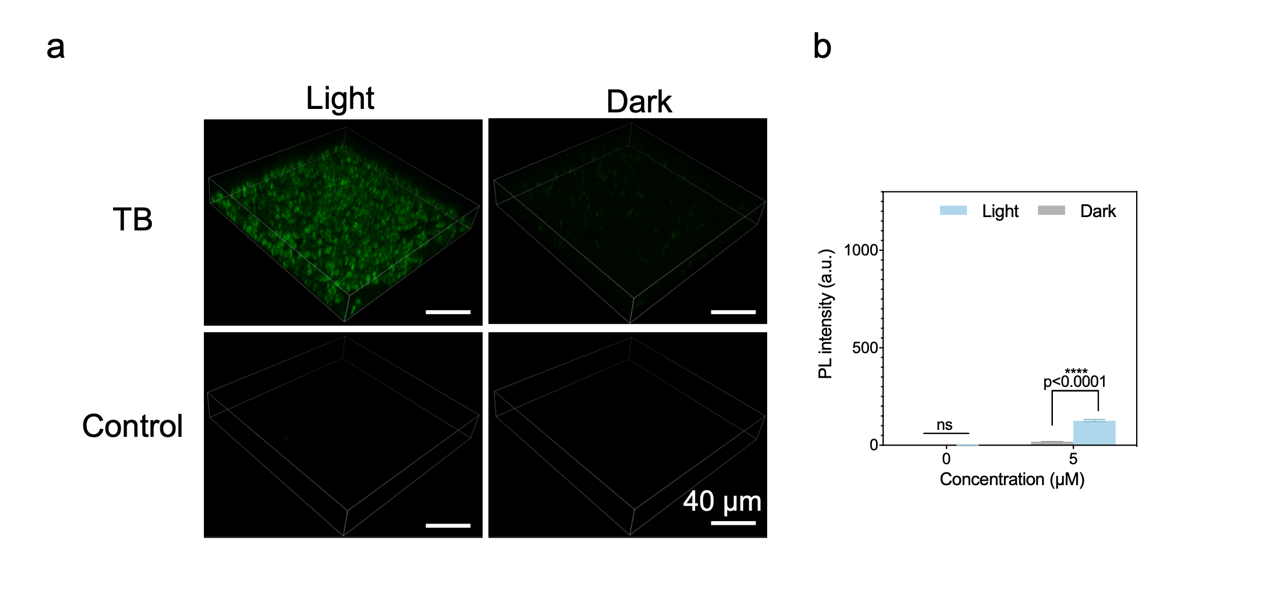


**Figure S17.** Evaluation of intracellular reactive oxygen species (ROS) production in biofilms. CLSM 3D images of biofilms (a) and quantification of the corresponding fluorescence intensity (b) according to COMSTAT 2.0 for five random observation points of *C. auris* biofilms. The green channel uses a 488 nm laser and a 515–550 nm emission filter.


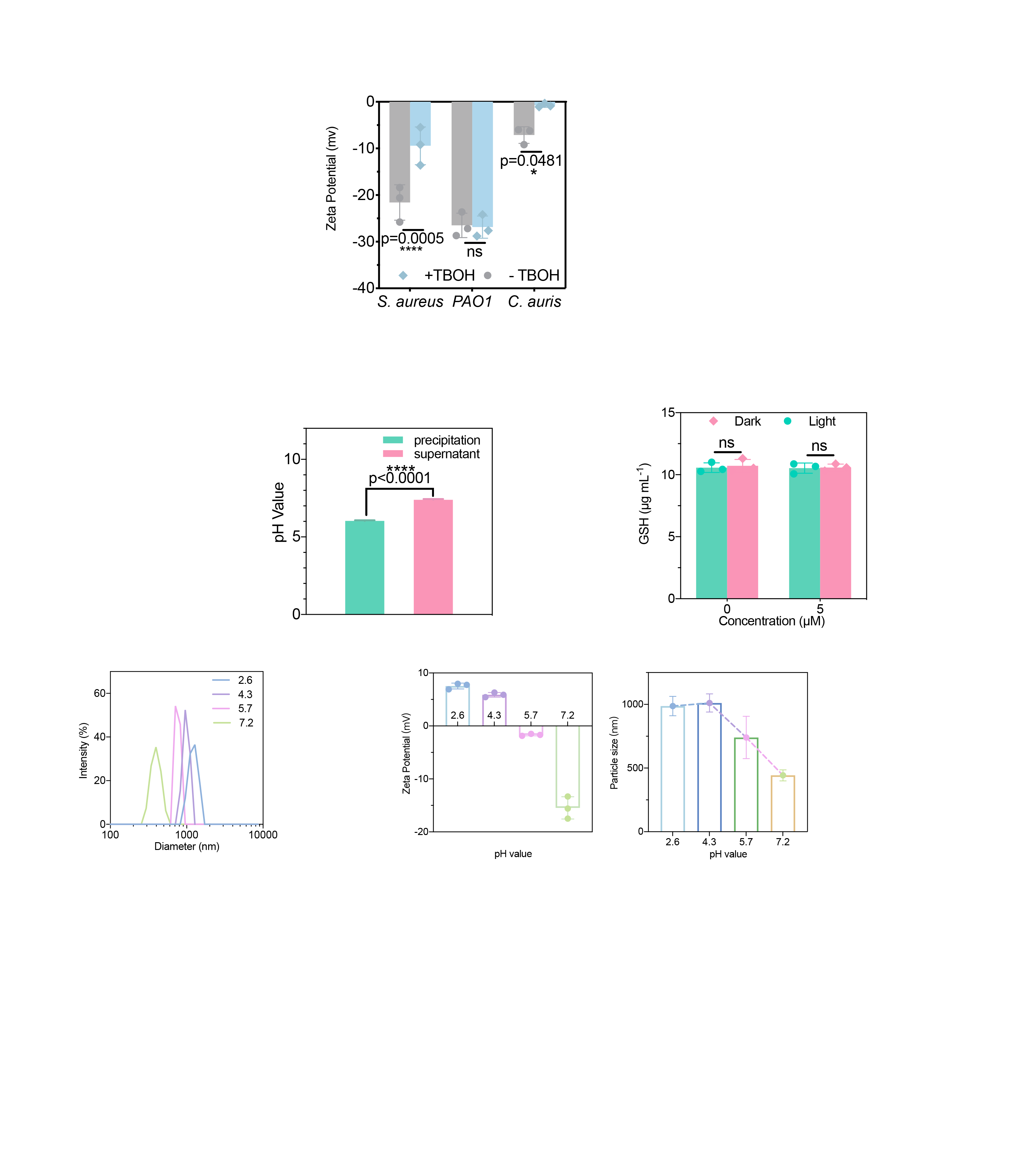


**Figure S18.** The pH value of the precipitation biofilm region and the supernatant biofilm region.


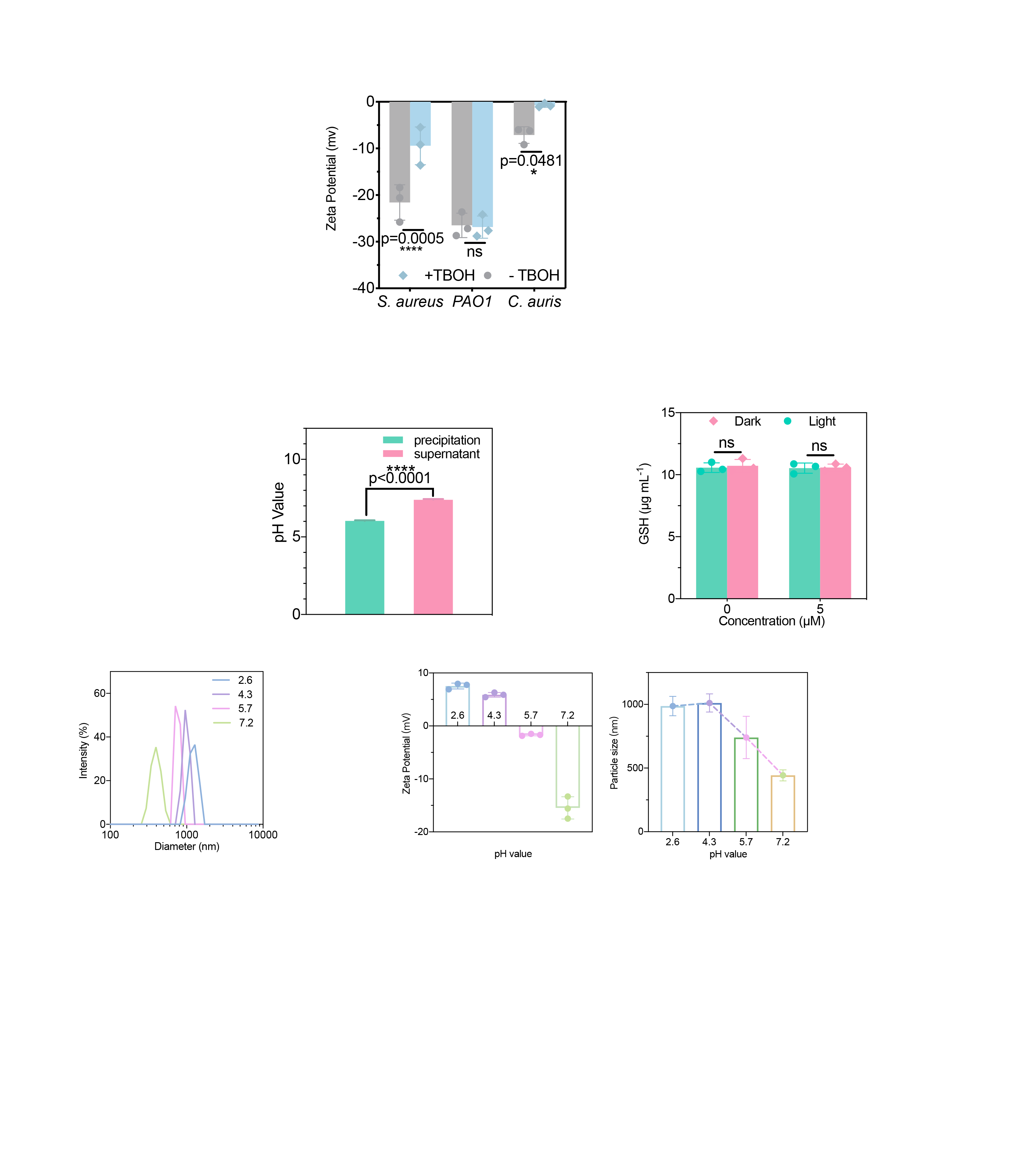


**Figure S19.** The concentration of GSH in biofilm after different treatments.


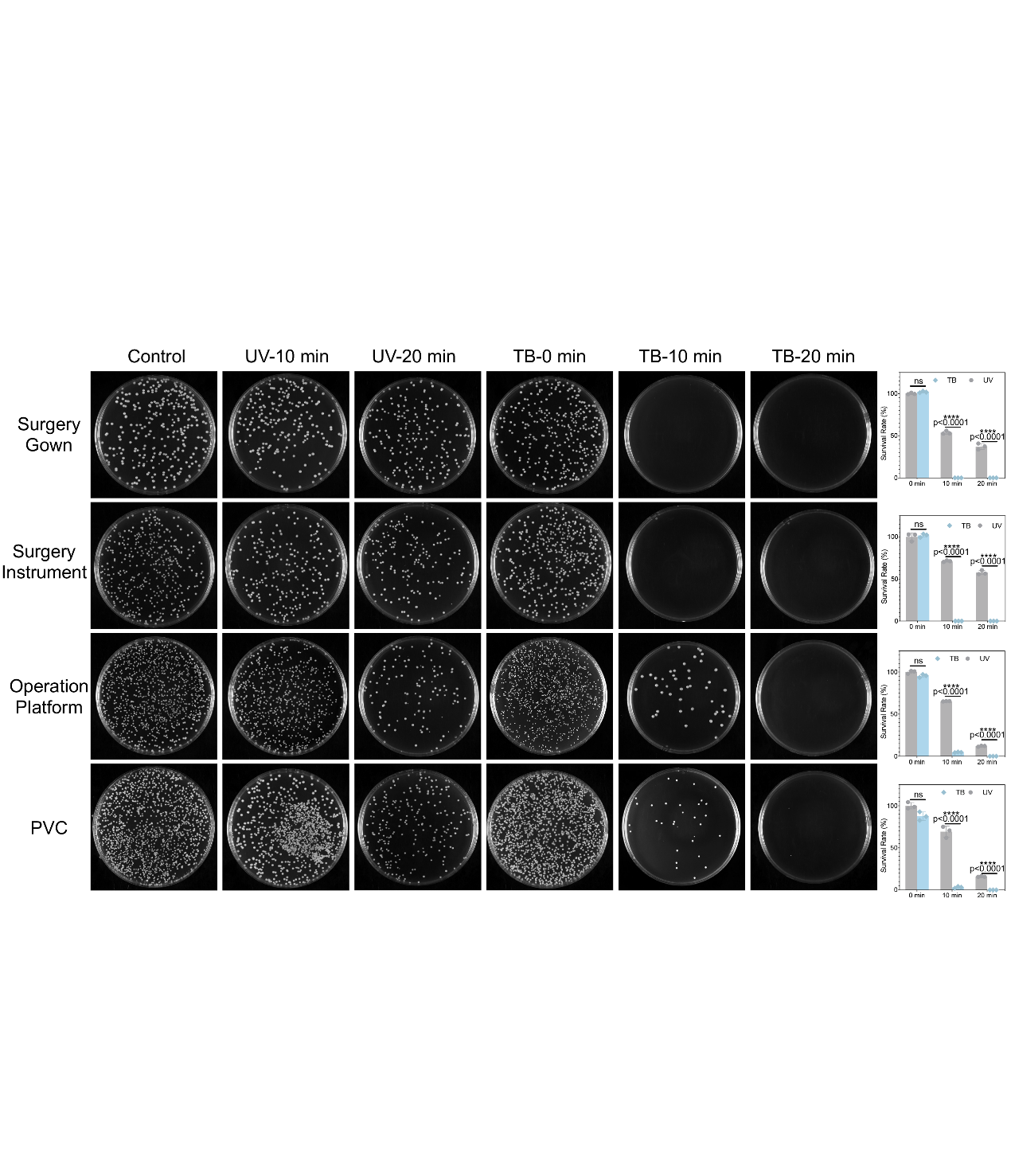


**Figure S20.** PDI effects on different high-touch surfaces. Representative agar images of *C. auris* were treated with 10-min and 20-min irradiation under UV and 0-min, 10-min, and 20-min irradiation under white light with TB.


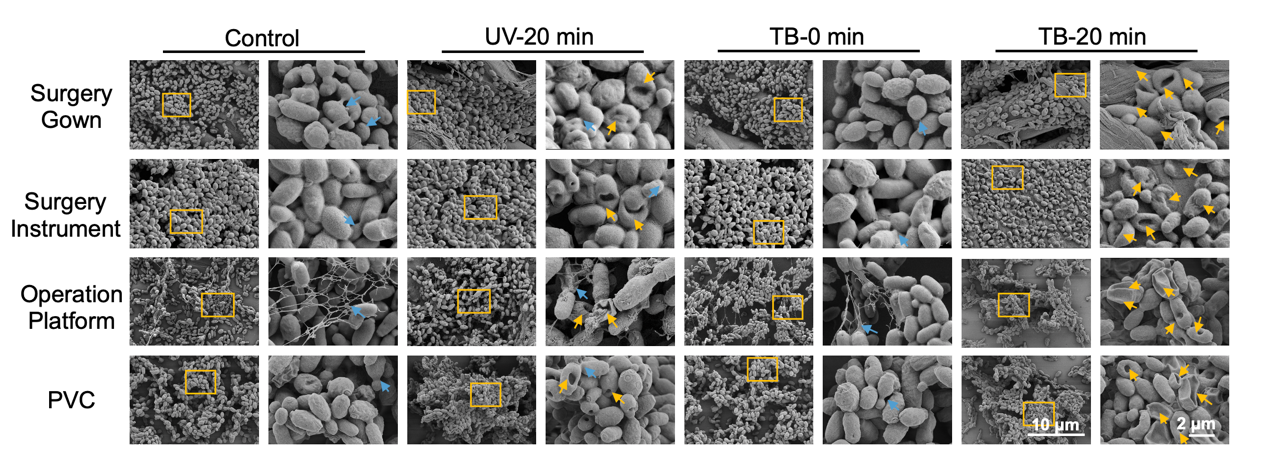


**Figure S21.** The morphology of the biofilm on different high-touch surfaces. Representative FESEM images of *C. auris* biofilms on TB-functionalized materials (surgery grown, surgical instruments, operation platform, and PVC) treated with 10 μM TB in the dark or irradiated with white light (80 mW cm^-2^) or UV (200 μW cm^-2^) for 20 min (scale bar: 10 μm and 2 μm).


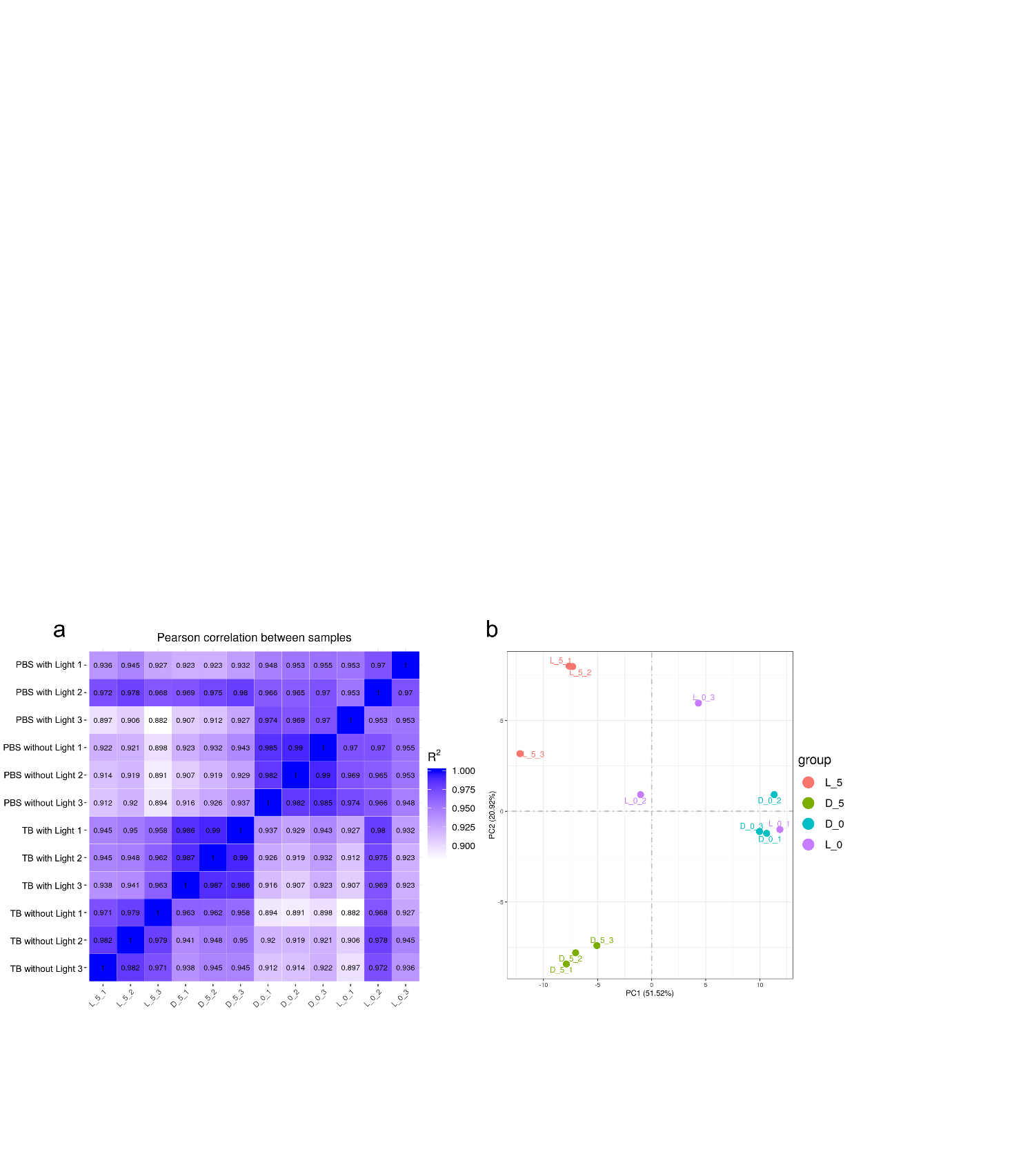


**Figure S22.** Quantitative analysis of samples. a) The heat map shows the correlation analysis between the different samples. Both horizontal and vertical axes represented each sample. Different colors represented different correlation coefficients. b) The principal component analysis (PCA) of tested samples. L_0:PBS with Light; D_0: PBS without Light D_5: TB without Light; L_5: TB with Light.


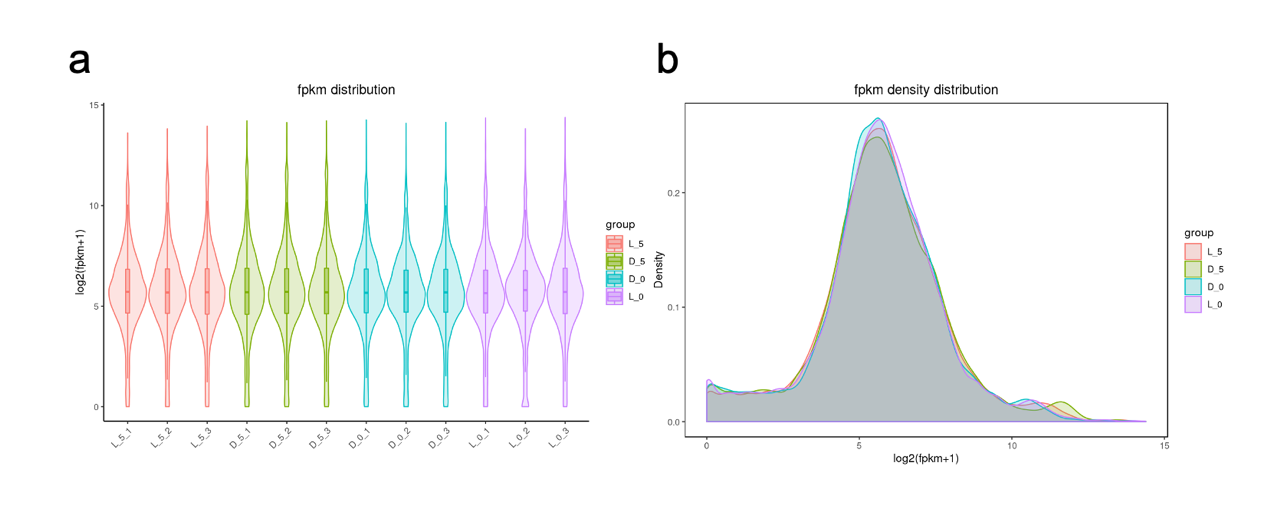


**Figure S23.** Gene expression levels under different experimental conditions. L_0:PBS with Light; D_0: PBS without Light D_5: TB without Light; L_5: TB with Light.


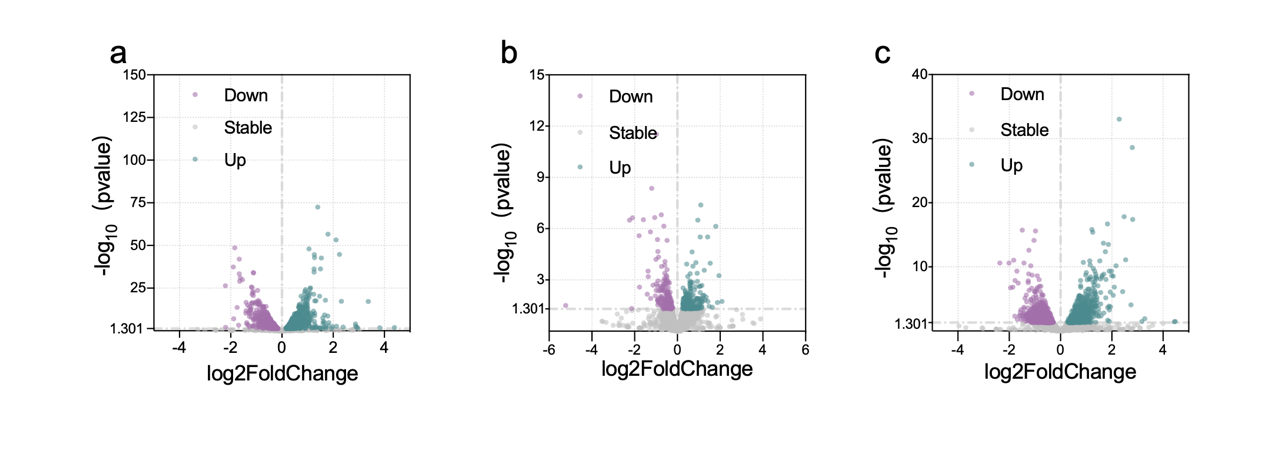


**Figure S24.** The volcano plots of TB with light vs TB without light (a), PBS with light vs PBS without light (b), and TB with light vs PBS without light (c). Green dots represent up-regulated genes, whereas purple dots present down-regulated genes (|log2foldChange|>0, -log10pvalue>1.301).


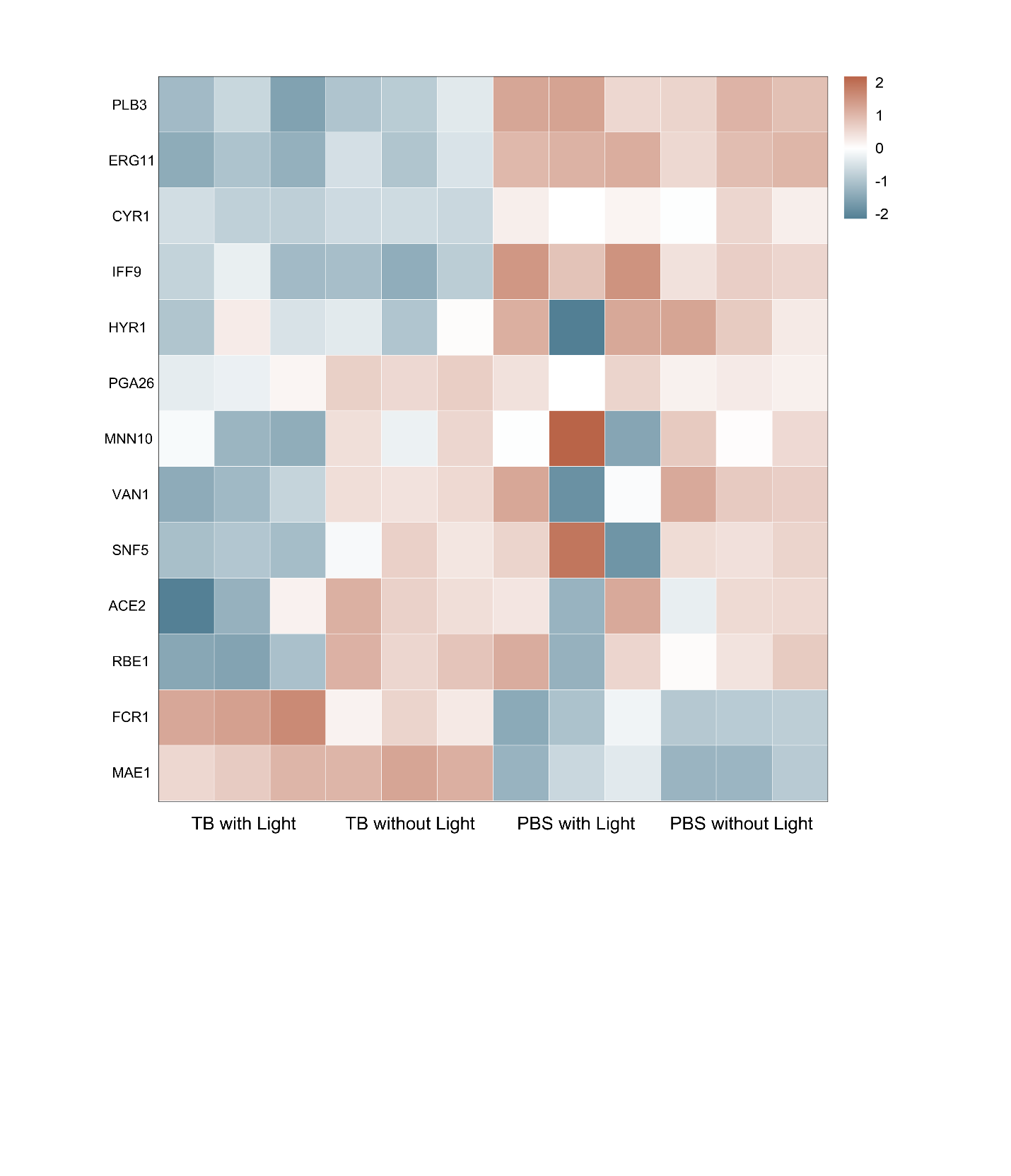


**Figure S25.** Clustering heatmap of DEGs in pathways related to biofilm regulation and basic metabolism.


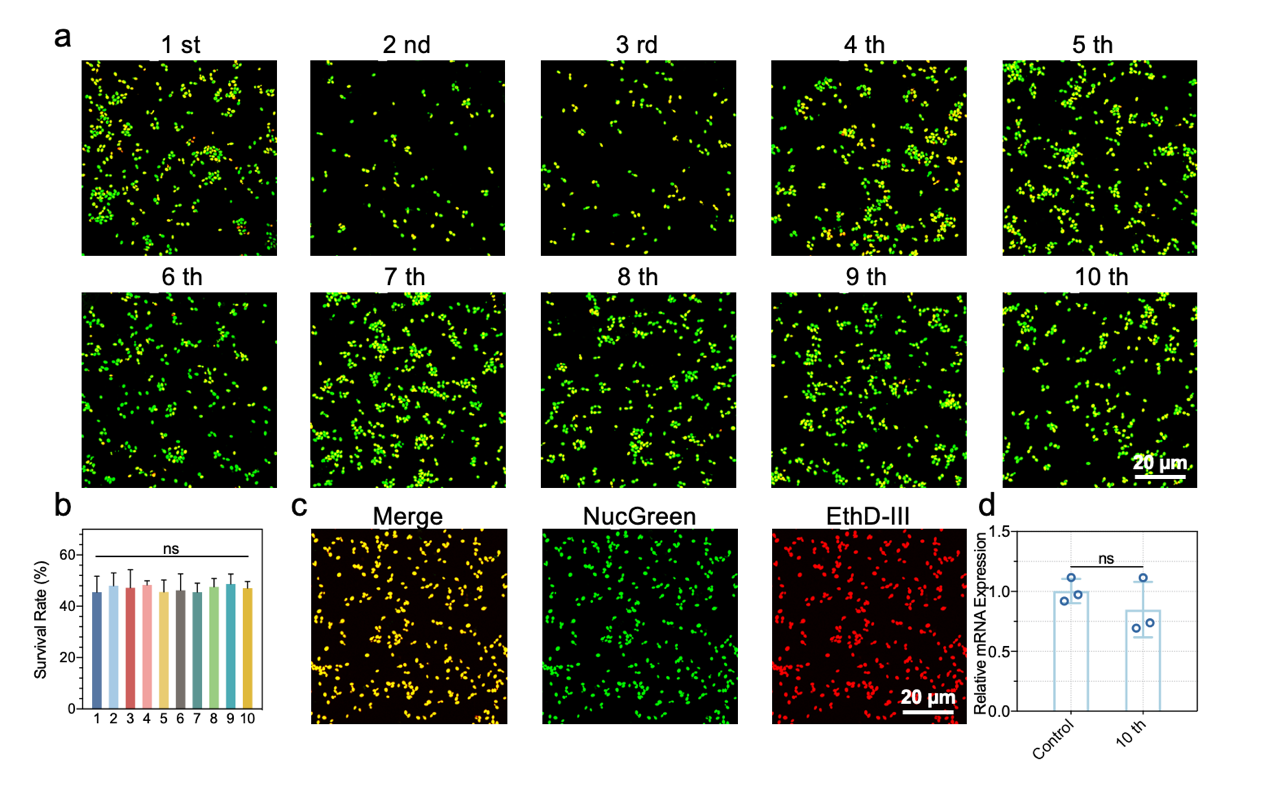


**Figure S26.** a-b) Respective CLSM images (a) and quantification (b) of PDI of *C. auris* after ten consecutive cycles of treatment with 1 μM TB for 15 min and irradiation with white light for 20 min (80 mW cm^-2^) followed by staining with a Live & Dead^TM^ activity/cytotoxicity analysis kit (UElandy). (scale bar: 20 μm). c) Photodynamic inactivation of *C. auris* after ten consecutive cycles of treatment with 5 μM TB for 15 min and irradiation with white light for 20 min (80 mW cm^-2^), followed by staining with a Live & Dead^TM^ activity/cytotoxicity analysis kit. The green channel utilized a 488 nm laser and a 515–550 nm emission filter, while the red channel employed a 561 nm laser and a 570–620 nm emission filter (scale bar: 20 μm). d) mRNA expression of *ERG11* before and after *C. auris* was treated with ten PDI cycles. Untreated fungi were used as a control. Data are shown as mean ± SD.

**
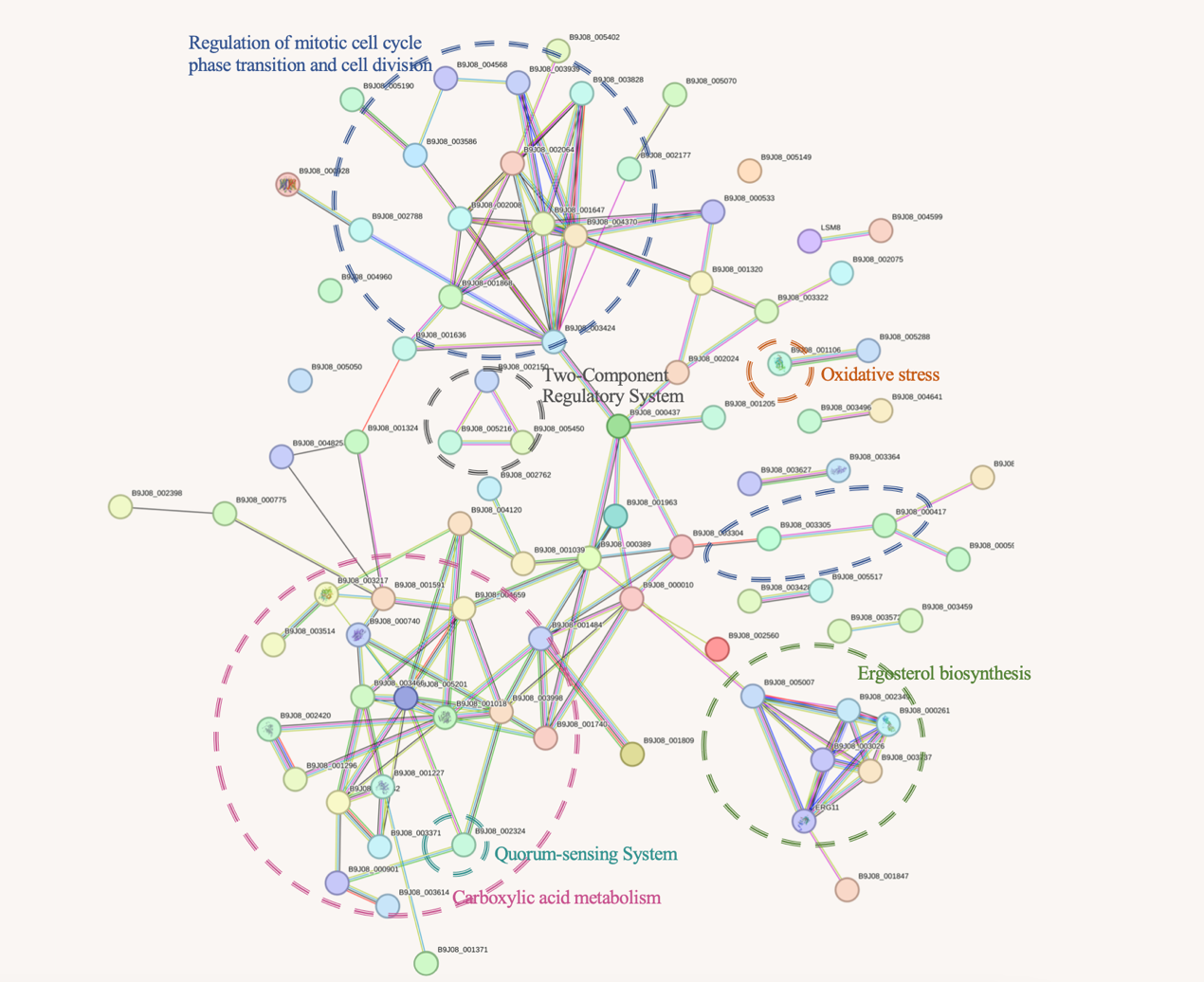
**

**Figure S27.** Protein-protein interaction network (PPI) diagram. Threshold settings: p-value or padj < 0.05; |Log2 (FoldChange)| > 2; Minimum required interaction score in PPI analysis is medium confidence (0.400).


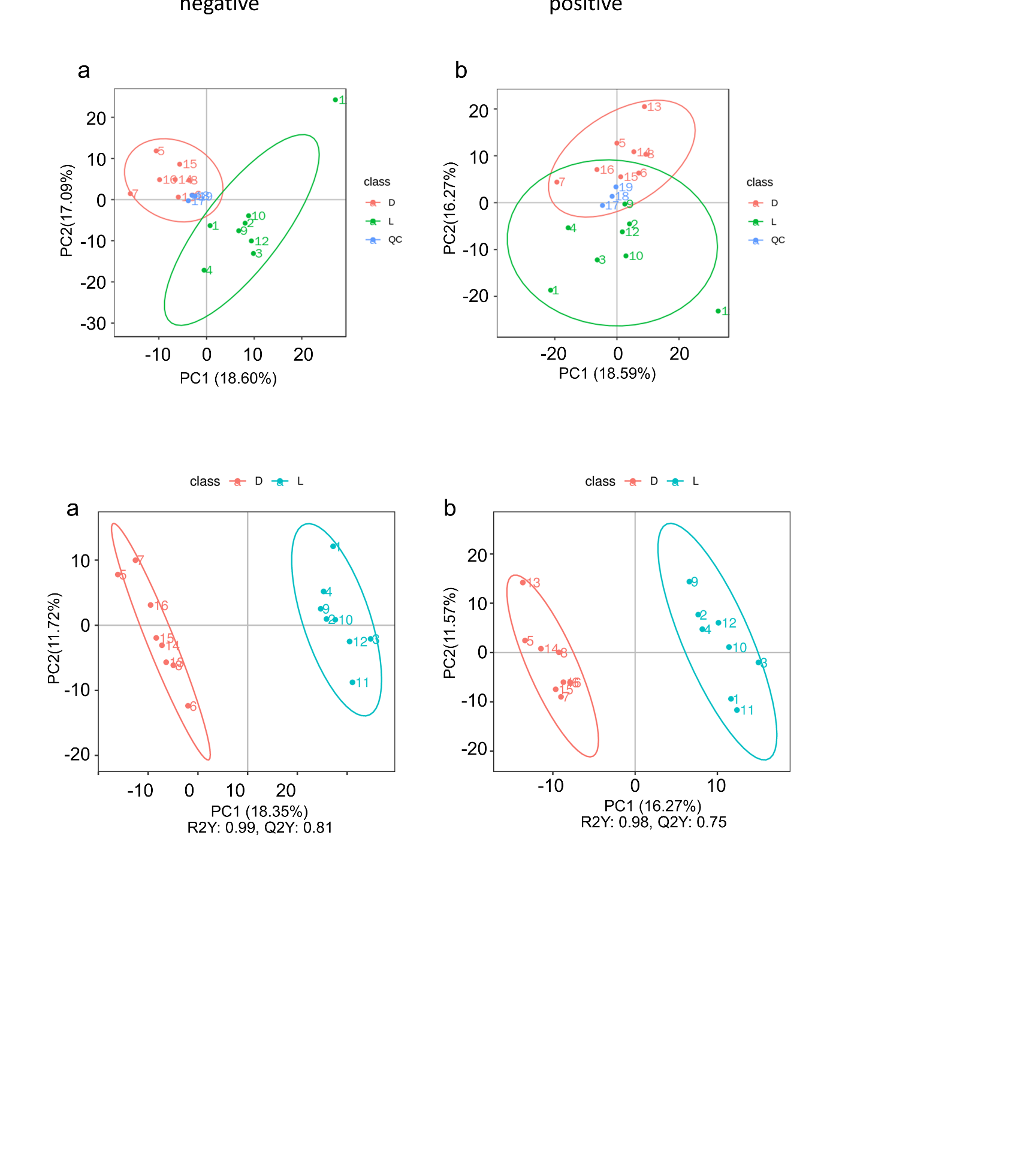


**Figure S28.** Total sample PCA analysis graph. The degree of clustering of QC samples in the map positively correlates with individual method stability and data quality of negative ions (a) and positive ions (b). QC: quality control Antibodies used in this study. D: PBS without Light; L: TB with Light.


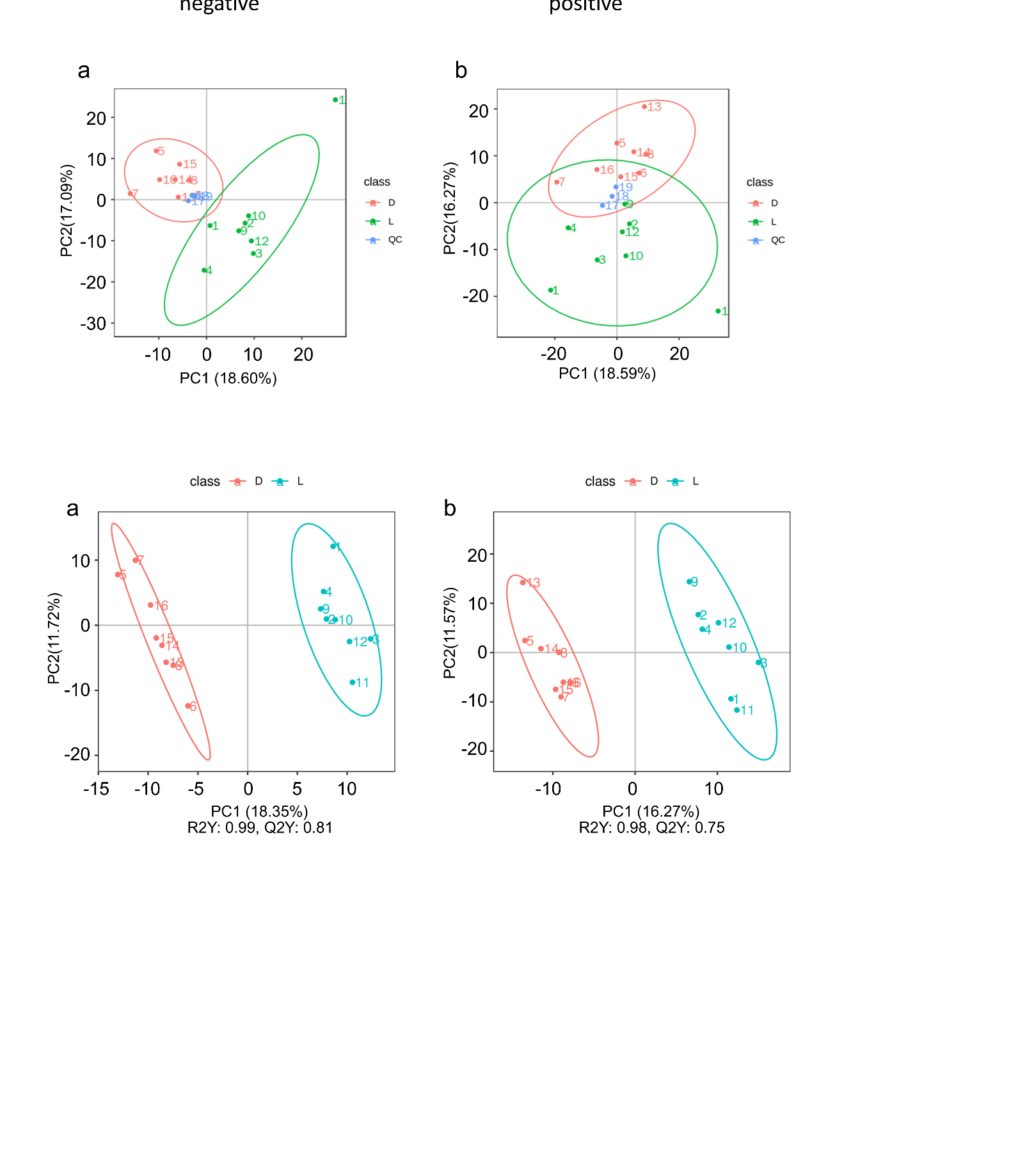


**Figure S29.** The OPLS-DA score plot for the fungi samples from the TB with light group versus the PBS without light group in the negative (a) and positive (b) ion modes, respectively. D: PBS without Light; L: TB with Light.


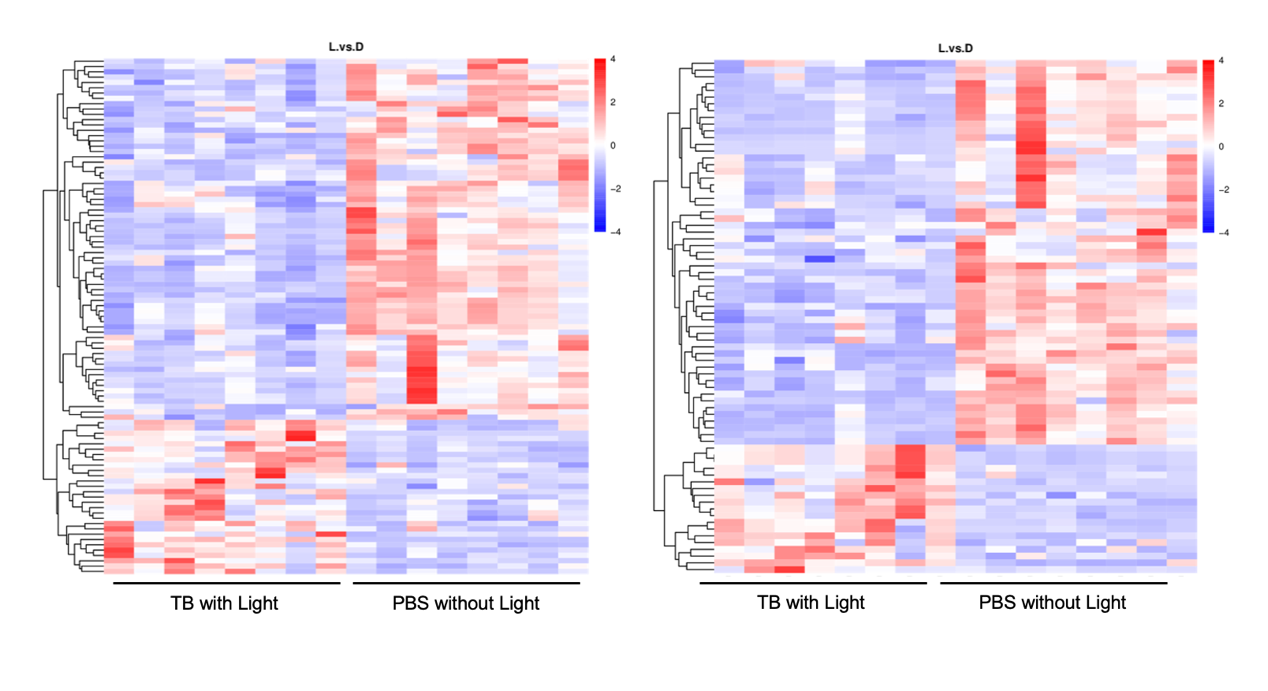


**Figure S30.** The hierarchical clustering heat map of DAMs for the biofilm samples from the TB with light group versus the PBS without light group in the negative ion modes (left Figure) and positive ion modes (right Figure), respectively. D: PBS without Light; L: TB with Light.


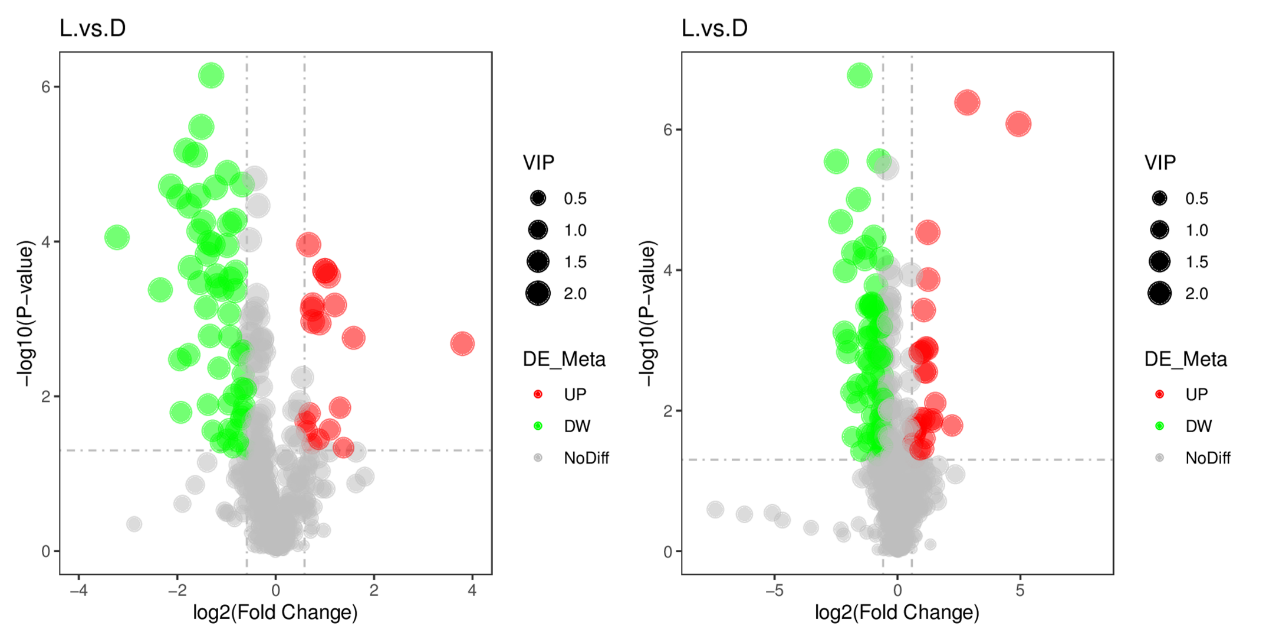


**Figure S31.** The volcano plots of DAMs for the biofilm samples from the TB with light group versus the PBS without light group in the negative ion modes (left Figure) and positive ion modes (right Figure), respectively. D: PBS without Light; L: TB with Light.


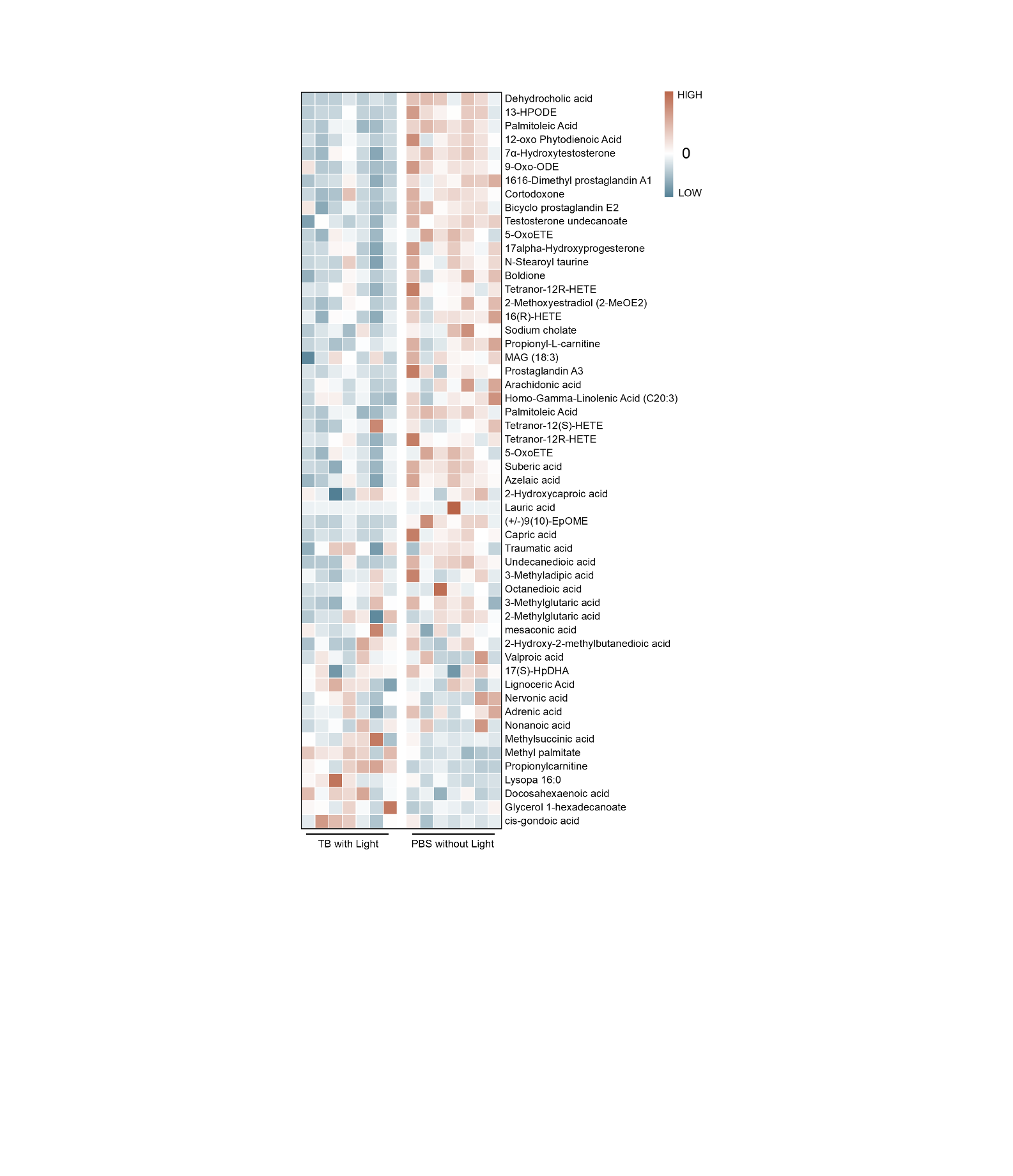


**Figure S32.** Clustering heatmap of lipids and lipid-like molecules metabolites DAMs.


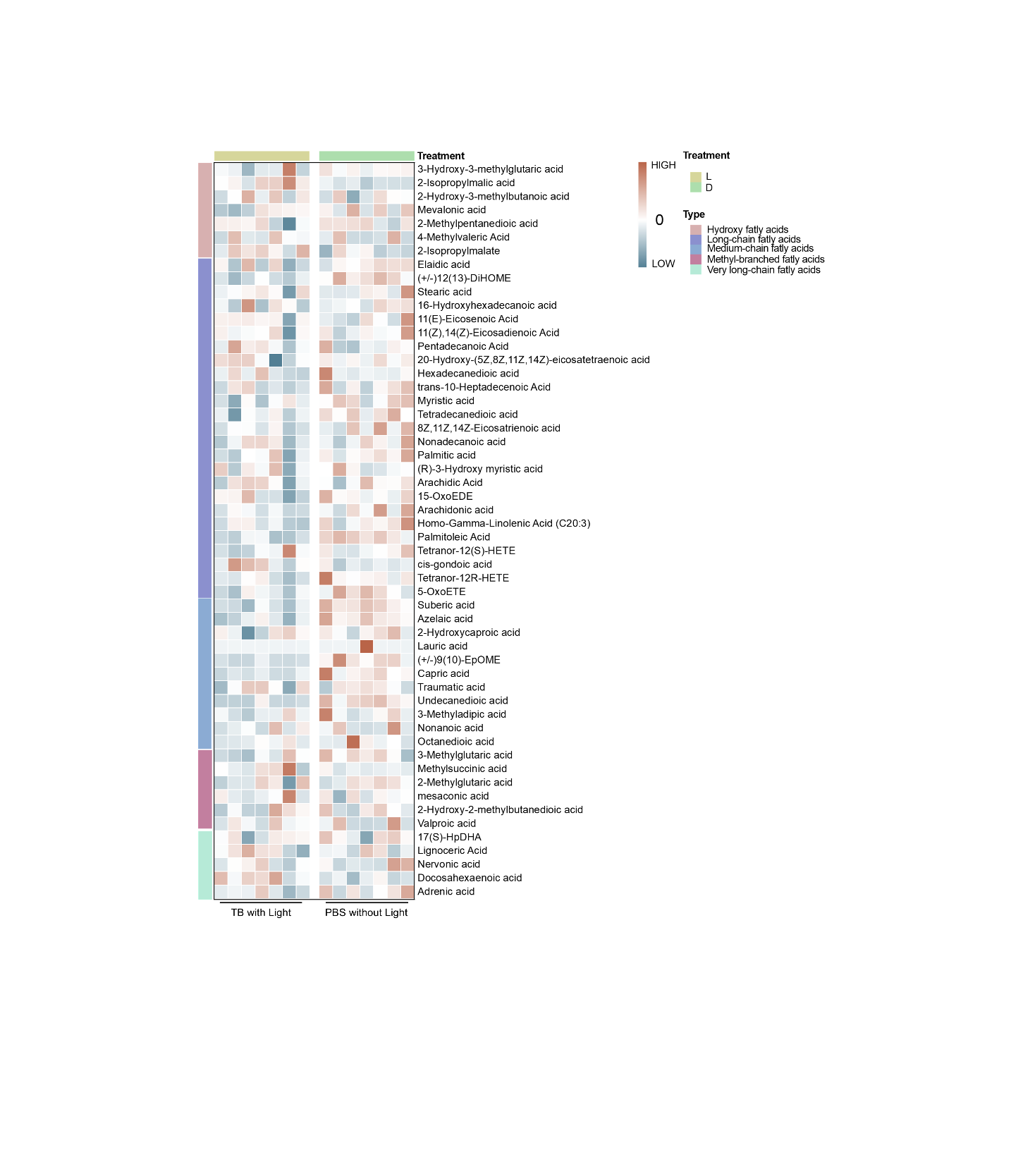


**Figure S33.** Clustering heatmap of the whole fatty acids and conjugates.


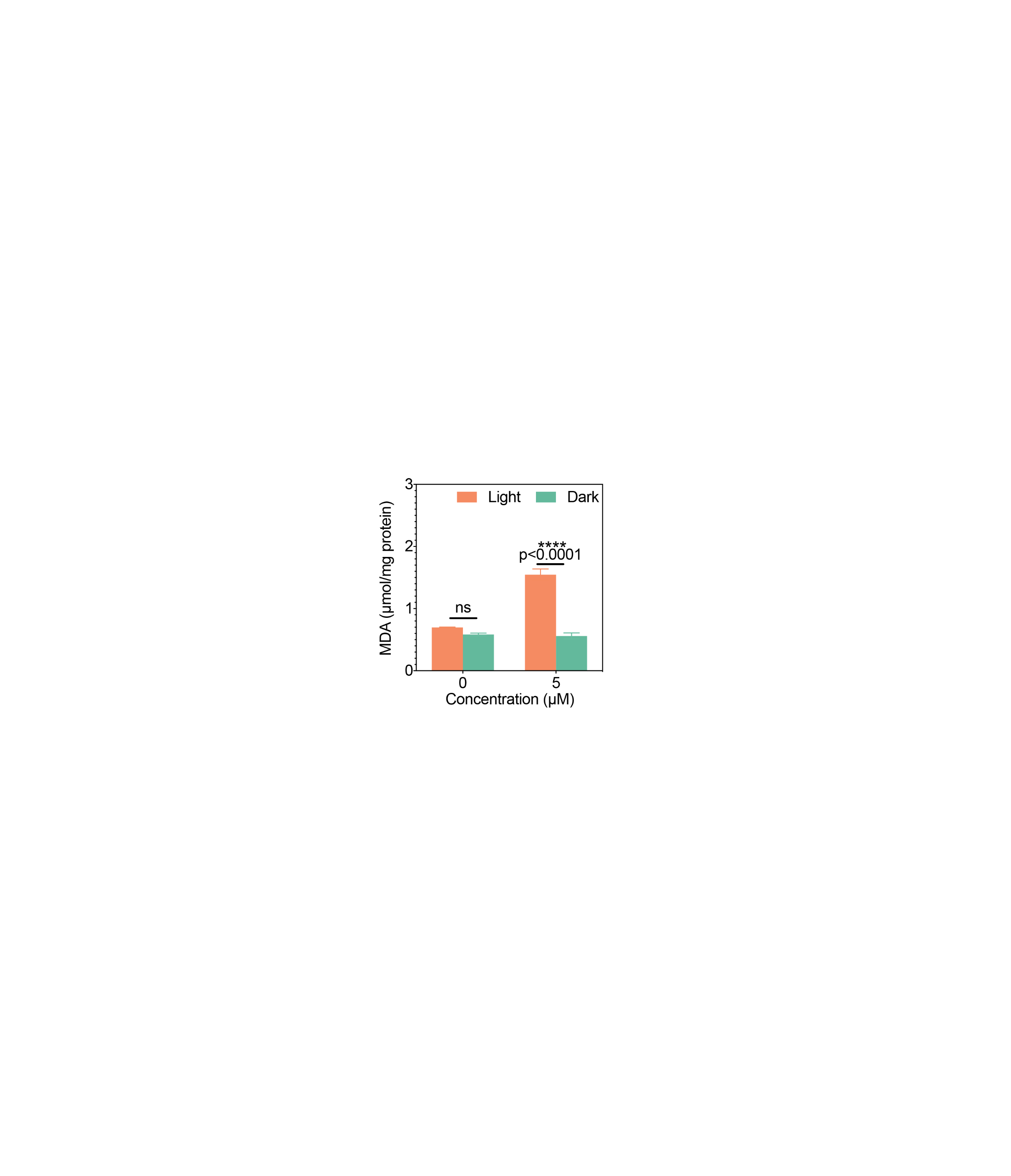


**Figure S34.** The concentration of MDA in mature biofilm treated with TB and PBS.


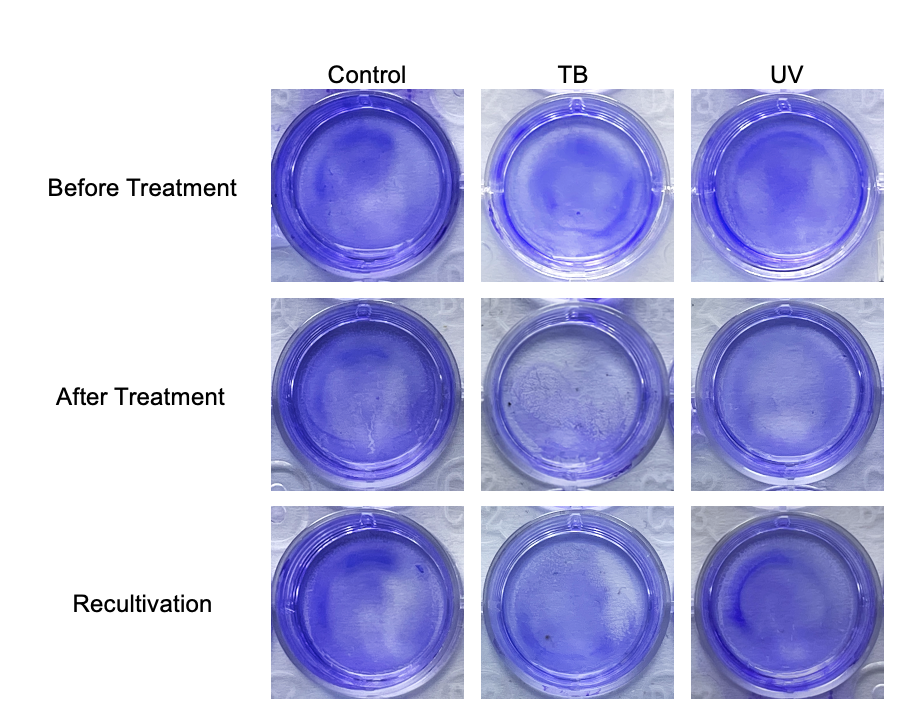


**Figure S35.** Images of mature biofilm before and after treated with TB and UV.


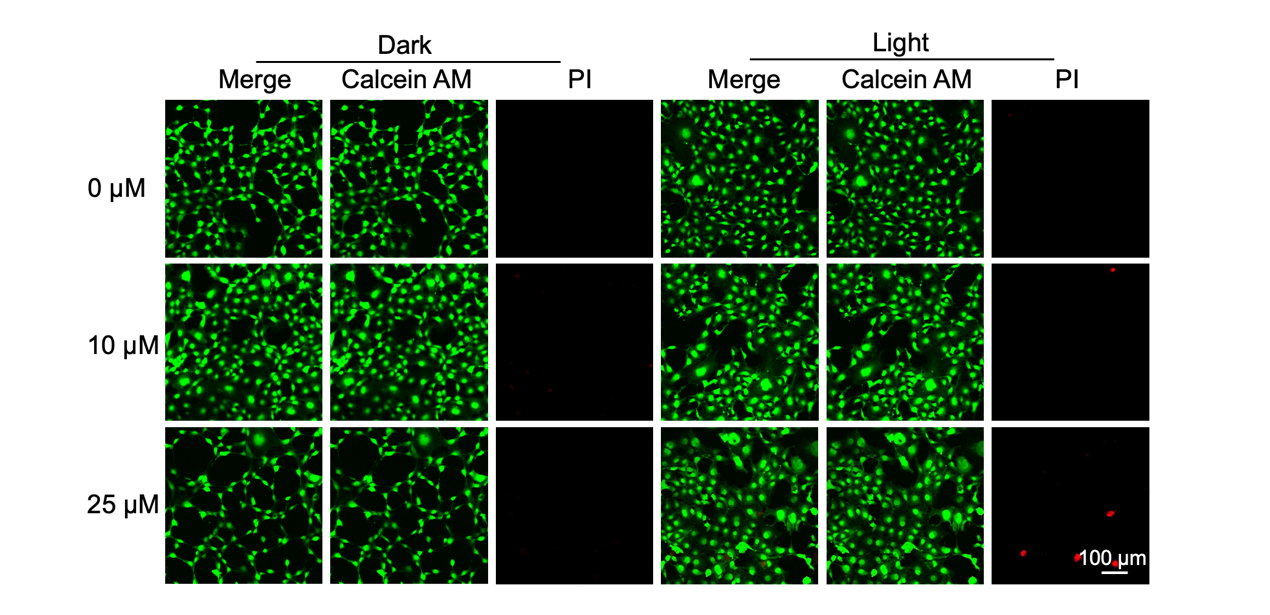


**Figure S36.** Fluorescence images of COS-7 cells pretreated with TB with or without white light irradiation. The cells were then stained with a Live & Dead^TM^ Animal Cell Viability/Cytotoxicity Assay Kit (Calcein AM, PI) and observed by CLSM. The green channel used a 488 nm laser and a 515–550 nm emission filter, and the red channel used a 561 nm laser and a 570–620 nm emission filter.


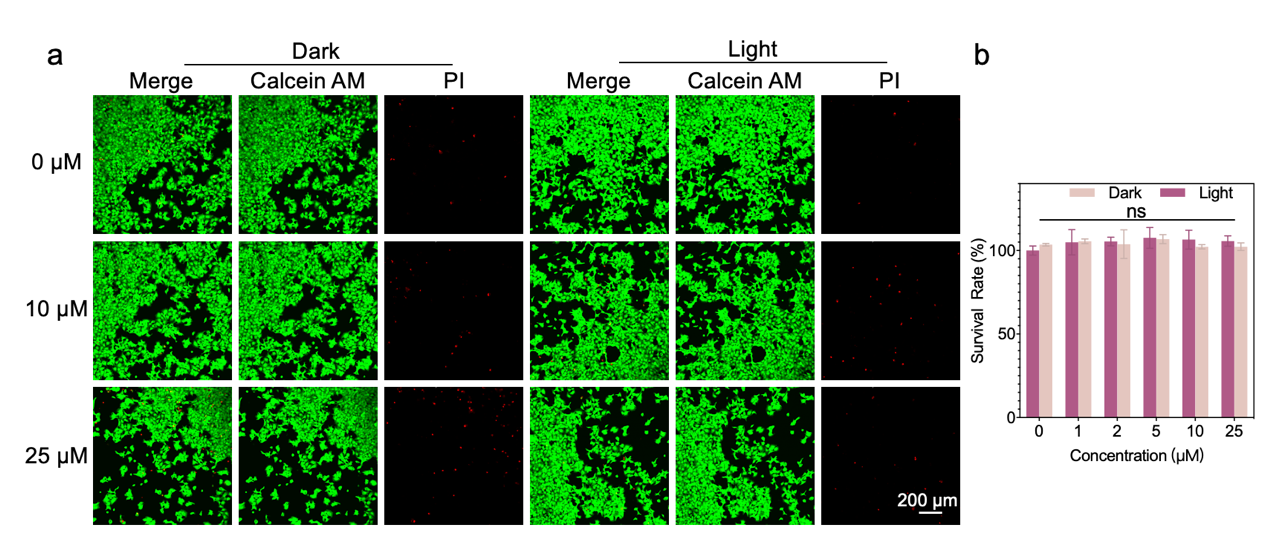


**Figure S37.** a) Live/Dead staining of HaCat cells. b) The survival Rate of HaCat cells. This result was obtained through the CCK-8 experiment.


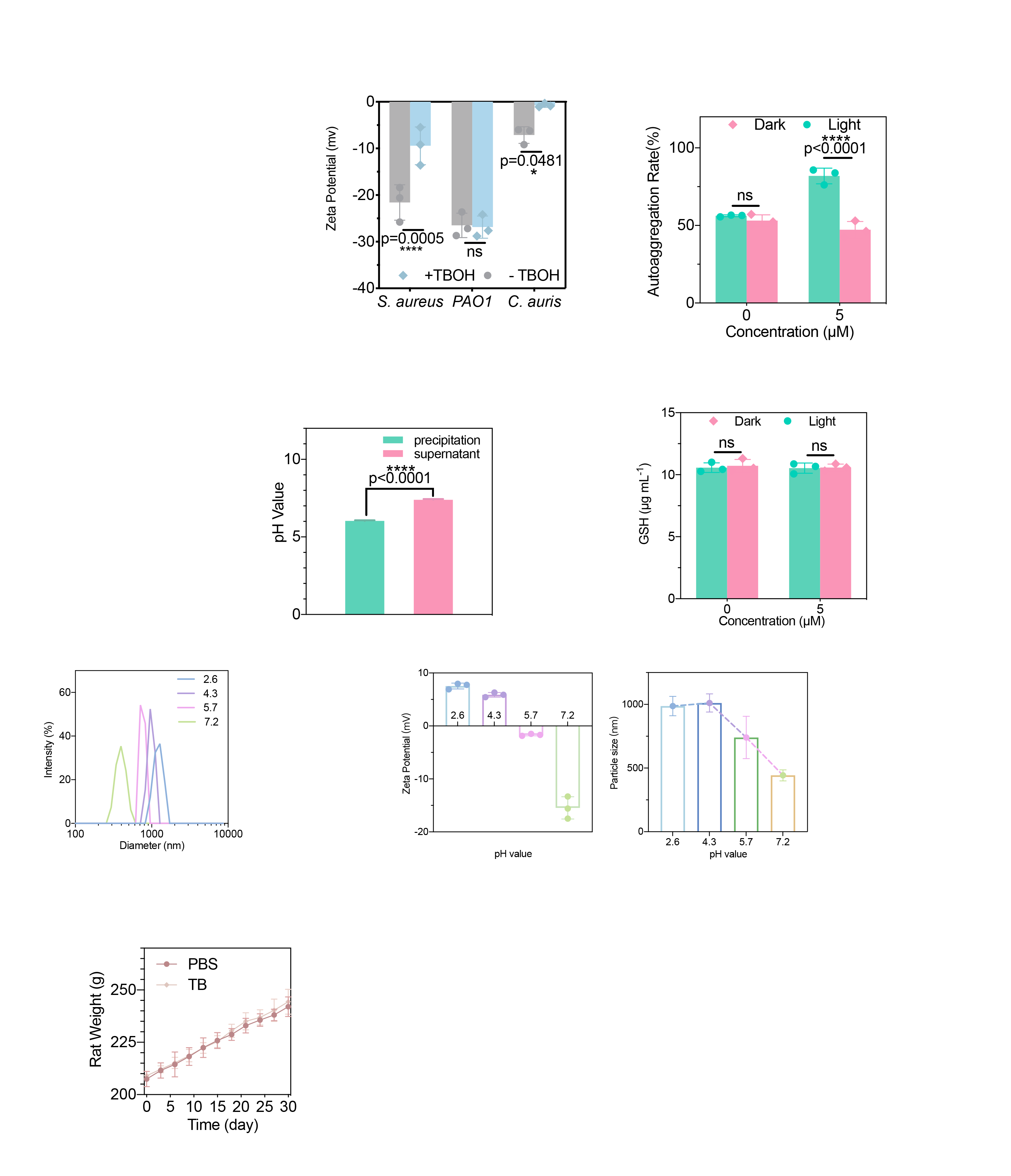


**Figure S38.** Weight change of long-term toxicity test rats in 30 days.


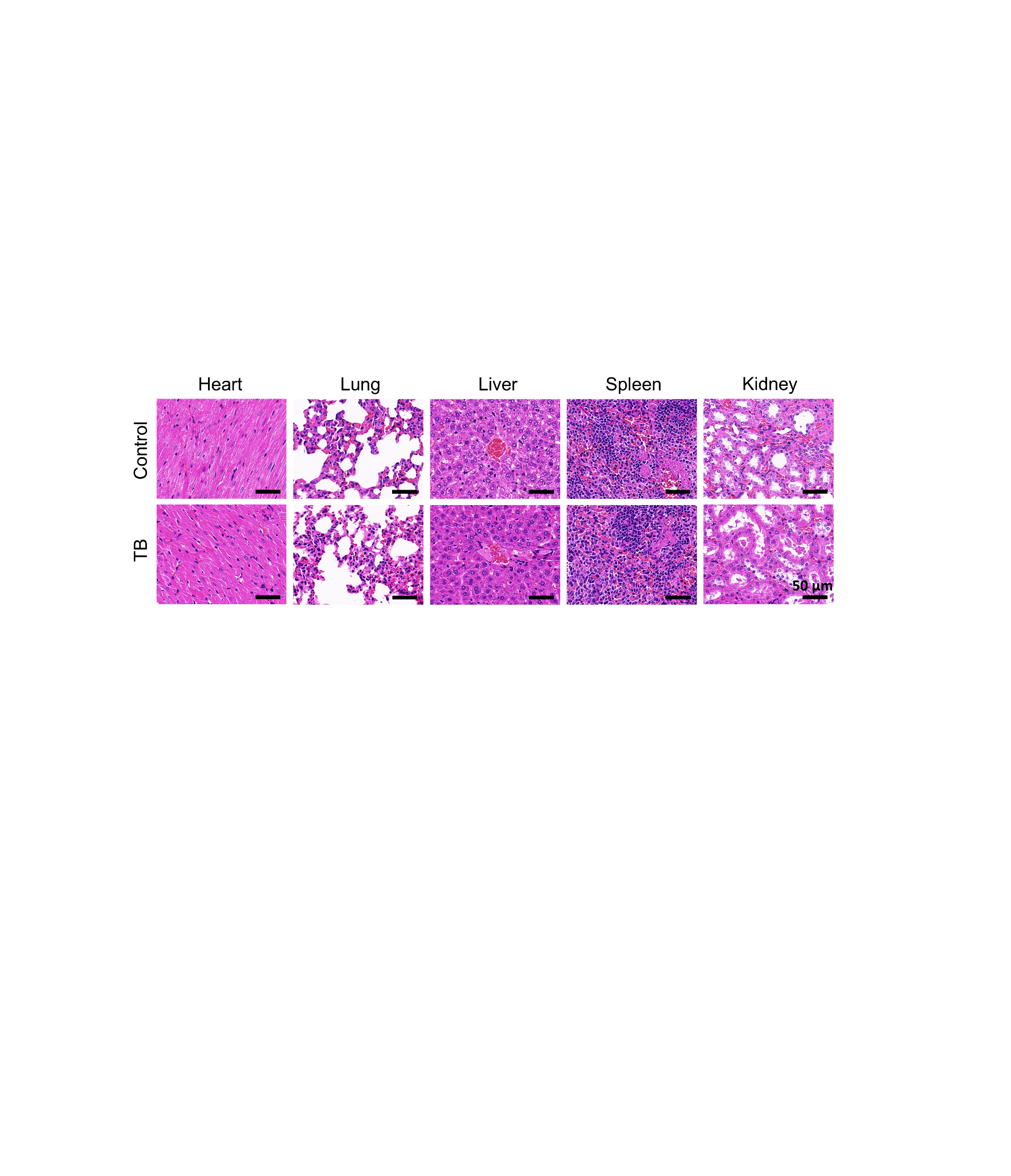


**Figure S39.** Images of different HE-stained vital organ tissues of healthy rats 30 days after intranasal treatment of 30 μL of TB solution (30 μM in PBS) or 30 μL of PBS. Scale bars: 50 μm.


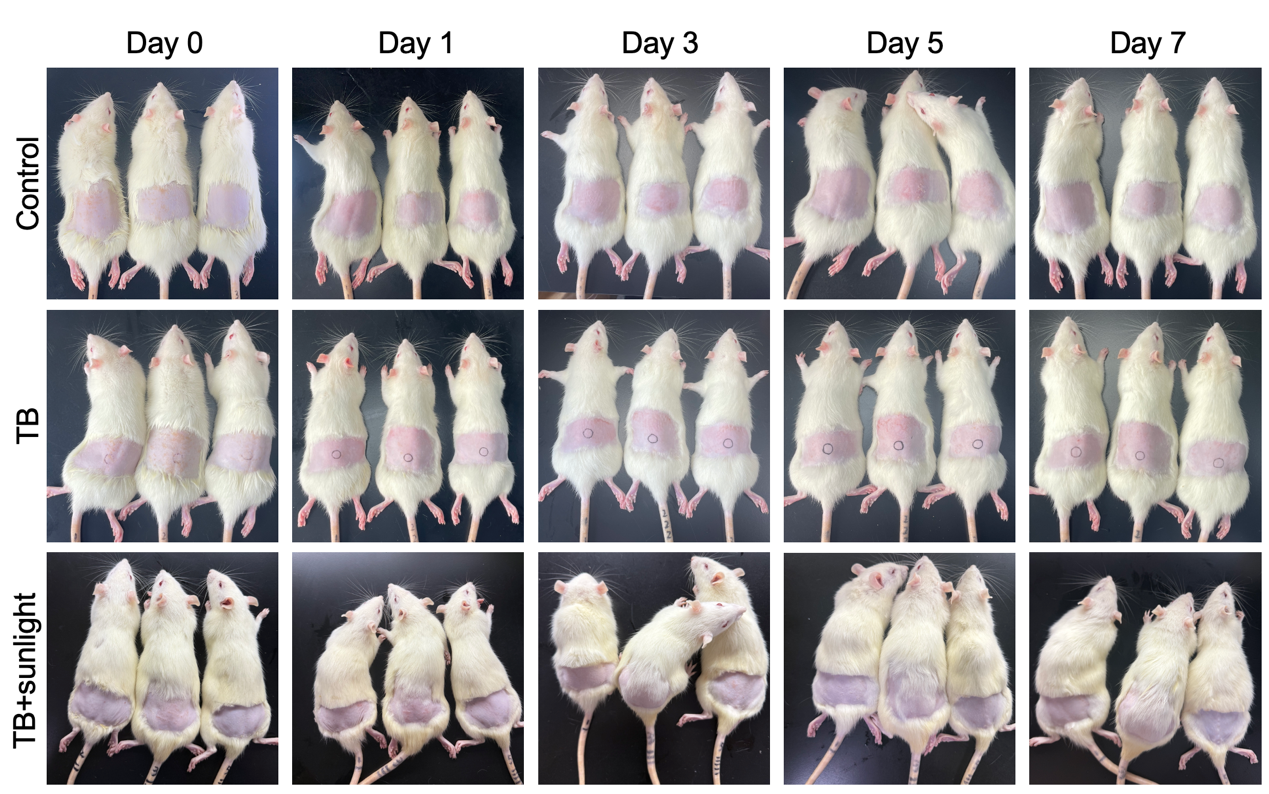


**Figure S40.** Images of rats’ skins after daily smearing of PBS, 30 μM TB with white light treatment ( 20 min and 80 mW cm^-2^), 30 μM TB with sunlight on the rats’ skin for different days.

**Supplemental Tables**

**Table S1.** Primers used in qRT-PCR for detecting *ACT1*, *PGA26*, *MNN10*, *RBE1*, *ERG11*, *PLB3* and *VAN1* levels.

| **primers** | **Fp (5’ to 3’)** | **Rp (5’ to 3’)** |
| --- | --- | --- |
| *ACT1* | CCTCTCAGTCGTCCGCTAT | CTTCATGGAAGATGGGGCTA |
| *PGA26* | CCAAGTGTGAGGAGGTTTGC | GGAGGTGGTGGTGTGGTAAG |
| *MNN10* | CCTTCTTCACCCTGATGCCT | CCATTCCTGTGGTCCTTTCCA |
| *RBE1* | GCCAGTACACGATTTGCGAG | CGTTGGCACCGAAGAACTTG |
| *ERG11* | TGCCCATCGTCTACAACCTT | GCACCCAGTGAAACACCAAG |
| *PLB3* | AGCCTGTGAACGATGAGTGT | CGGGTAAGCGAAGAAGACCT |
| *VAN1* | TGGTTTCCGACTGTTCTCCTG | AAGTTCGTCTGCCAATGTGC |

**Table S2.** Detailed descriptions of GO bubble diagram.

| **Ontology** | **Term** | **Description** |
| --- | --- | --- |
| Biological process | GO:0032940 | secretion by cell |
|  | GO:0046903 | secretion |
|  | GO:0006733 | oxidoreduction coenzyme metabolic process |
| Cellular component | GO:0051179 | localization |
|  | GO:0016020 | membrane |
|  | GO:0099023 | tethering complex |
|  | GO:0012505 | endomembrane system |
|  | GO:0005783 | endoplasmic reticulum |
|  | GO:0044425 | membrane part |
|  | GO:0044432 | endoplasmic reticulum part |
|  | GO:0071944 | cell periphery |
|  | GO:0098796 | membrane protein complex |
|  | GO:0005789 | endoplasmic reticulum membrane |
|  | GO:0042175 | nuclear outer membrane-endoplasmic reticulum membrane network |
|  | GO:0098827 | endoplasmic reticulum subcompartment |
|  | GO:0031984 | organelle subcompartment |
|  | GO:0031410 | cytoplasmic vesicle |
|  | GO:0031982 | vesicle |
|  | GO:0044433 | cytoplasmic vesicle part |
|  | GO:0097708 | intracellular vesicle |
|  | GO:0005794 | Golgi apparatus |
|  | GO:0044431 | Golgi apparatus part |
| Molecular function | GO:0008092 | cytoskeletal protein binding |
|  | GO:0016773 | phosphotransferase activity, alcohol group as acceptor |
|  | GO:0005543 | phospholipid binding |

**Table S3.** Detailed descriptions of the PPI diagram.

| Functional enrichments | STRING protein | Query sequence | Gene Name |
| --- | --- | --- | --- |
| Two-component regulatory system | B9J08_002150 | 40026778 | *SLN1* |
|  | B9J08_005216 | 40030443 | *SSK2* |
|  | B9J08_005450 | 40030677 | *SSK1* |
| Ergosterol biosynthesis | B9J08_005007 | 40030235 | *ERG7* |
|  | B9J08_002349 | 40027039 | *ERG5* |
|  | B9J08_000261 | 40025409 | *ERG1* |
|  | B9J08_003026 | 40028243 | *ERG24* |
|  | B9J08_003737 | 40028956 | *ERG3* |
|  | ERG 11 | 40026303 | *ERG 11* |
| Carboxylic acid metabolism | B9J08_003217 | 40028438 | *HOM3* |
|  | B9J08_001591 | 40026866 | *GAD1* |
|  | B9J08_004659 | 40029938 | *UGA2* |
|  | B9J08_001484 | 40026267 | *THIKB* |
|  | B9J08_003514 | 40028732 | *MET17* |
|  | B9J08_000740 | 40025888 | *GDHA* |
|  | B9J08_003998 | 40029170 | *ACS1* |
|  | B9J08_001740 | 40027547 | *POX1* |
|  | B9J08_003466 | 40028685 | *MDH1* |
|  | B9J08_005201 | 40030429 | *PYC* |
|  | B9J08_001018 | 40026185 | *ACC1* |
|  | B9J08_002420 | 40027110 | *FAS2* |
|  | B9J08_001296 | 40026456 | *FAS1* |
|  | B9J08_001227 | 40026526 | *TDH1* |
|  | B9J08_003632 | 40028850 | *PYK1* |
|  | B9J08_003371 | 40028592 | *GPM1* |
|  | B9J08_002324 | 40027013 | *ARO80* |
|  | B9J08_000901 | 40026066 | *CDC14* |
|  | B9J08_003614 | 40028832 | *ADH2* |
| Regulation of mitotic cell cycle phase transition and cell division | B9J08_005070 | 40030300 | *DSE1* |
|  | B9J08_002064 | 40027401 | *NUF2* |
|  | B9J08_002177 | 40026752 | *AMN1* |
|  | B9J08_003586 | 40028804 | *SGS1* |
|  | B9J08_002008 | 40027345 | *ASE1* |
|  | B9J08_001647 | 40026810 | *CDH1* |
|  | B9J08_003424 | 40028644 | *CDC5* |
|  | B9J08_004370 | 40029578 | *CDC20* |
|  | B9J08_001868 | 40027675 | *CDC14* |
|  | B9J08_001636 | 40026821 | *mob1-1* |
|  | B9J08_003305 | 40028526 | *CDC25* |
|  | B9J08_000417 | 40025564 | *CDC24* |
| Oxidative stress | B9J08_001106 | 40026647 | *PRX1* |
| Quorum-sensing system | B9J08_002324 | 0027013 | *ARO80* |

**Table S4.** Hematologic analysis of healthy rats 3 days after intranasal treatment of 30 μL of TB solution (30 μM in PBS) or 30 μL of PBS.

| **Tests** | **Control** | **TB** | **Reference Range** |
| --- | --- | --- | --- |
| WBC (10^9/L) | 5.3 ± 2.23 | 8.4 ± 3.40 | 0.8–10.6 |
| Lymph# (10^9/L) | 3.93 ± 1.72 | 6.4 ± 2.98 | 0.6–8.9 |
| Mon# (10^9/L) | 0.17 ± 0.06 | 0.23 ± 0.06 | 0.04–1.4 |
| Gran# (10^9/L) | 1.2 ± 0.46 | 1.77 ± 0.38 | 0.23–3.6 |
| Lymph (%) | 72.9 ± 2.31 | 75.07 ± 5.71 | 40–92 |
| Mon (%) | 3.37 ± 0.60 | 2.93 ± 0.51 | 0.9–18 |
| Gran (%) | 23.73 ± 1.86 | 22 ± 5.26 | 6.5–50 |
| RBC (10^12/L) | 5.88 ± 1.63 | 7.55 ± 0.30 | 6.5–11.5 |
| HGB (g/L) | 113.33 ± 34.96 | 138.33 ± 9.29 | 110–165 |
| HCT (%) | 35.7 ± 10.46 | 45.53 ± 2.80 | 35–55 |
| MCV (fL) | 60.5 ± 1.87 | 60.33 ± 2.15 | 41–55 |
| MCH (pg) | 19.07 ± 1.16 | 18.3 ± 1.82 | 13–18 |
| MCHC (g/L) | 315.67 ± 8.62 | 304.33 ± 31.88 | 300–360 |
| RDW (%) | 10.6 ± 0.87 | 10.9 ± 0.8 | 12–19 |
| PLT (10^9/L) | 831.67 ± 542.00 | 1040 ± 587.33 | 400–1600 |
| MPV (fL) | 6.7 ± 0.17 | 6.7 ± 0.76 | 4.0–6.2 |
| PDW | 18.47 ± 1.00 | 16.9 ± 0.96 | 12.0–17.5 |
| PCT (%) | 0.12 ± 0.04 | 0.292 | 0.100–0.780 |

**Table S5.** Antibodies used in this study.

| **Antibodies** | **Company** | **Description** | **Cat** | **Dilution** |
| --- | --- | --- | --- | --- |
| IL-6 | Servicebio | Rabbit polyclonal antibody | GB11117 | 1:500 |
| TNF-α | Bioss | Rabbit polyclonal antibody | BS-10802R | 1:500 |
| Secondary antibody for TNF-α | Jackson | Alexa Fluor® 488-conjugated goat anti-rabbit IgG | 111-545-003 | 1:200 |

**Supplementary Auxiliary Files**

**Movie S1.** The movement path of *C. auris* on confocal dish. *C. auris* was treated with PBS in the dark.

**Movie S2.** The movement path of *C. auris* on confocal dish. *C. auris* was treated with TB spray in the dark.

**Movie S3.** The movement path of *C. auris* on confocal dish. *C. auris* was treated with PBS and white light (80 mW cm^-2^).

**Movie S4.** The movement path of *C. auris* on confocal dish. *C. auris* was treated with TB spray and white light (80 mW cm^-2^).

**Movie S5.** Different layers of *C. auris* biofilm images treated with NucGreen and TB. The green fluorescence represents *C.auris* in the biofilm, and the red fluorescence represents polysaccharides in the biofilm targeted by TB. A 488 nm laser and a 515–550 nm emission filter were employed for the green channel, and a 561 nm laser and a 570–620 nm emission filter were used for the red channel.
